# Supramolecular Benzophenone-Based Photoinitiator for Spatially-Resolved Polymerization

**DOI:** 10.1021/acsami.5c03506

**Published:** 2025-05-12

**Authors:** Alex S. Loch, Ibram Mikhail, Simona Bianco, Dipankar Ghosh, Ravi R. Sonani, Victor Chechik, Massimo Vassalli, Edward H. Egelman, Andrew J. Smith, Dave J. Adams

**Affiliations:** † School of Chemistry, 3526 University of Glasgow, Glasgow G12 8QQ, U.K.; ‡ Department of Biochemistry and Molecular Genetics, 214841 University of Virginia, Charlottesville, Virginia 22903, United States; § Department of Chemistry, 8748 University of York, York YO10 5DD, U.K.; ∥ Centre for the Cellular Microenvironment, University of Glasgow, Glasgow G12 8LT, U.K.; ⊥ Diamond House, Harwell Science and Innovation Campus, 120796 Diamond Light Source Ltd., Didcot, Oxfordshire OX11 0DE, U.K.

**Keywords:** materials chemistry, soft materials, supramolecular gel noodles, micelle, benzophenone, photoinitiator, photopolymerization

## Abstract

Benzophenone-based materials remain widely used as photoinitiators for ultraviolet light-induced free radical polymerizations. Traditionally, polymerization is spatially controlled using top-down techniques such as photomasks, which produce well-defined polymeric films. In contrast, we present an alternative method for controlling polymerization by employing supramolecular materials to localize the photoinitiator. This approach uses benzophenone-functionalized dipeptides that are specifically tuned to enable supramolecular gel noodle formation, which act as structural templates. We show that polymerization of acrylate monomers around the gel noodles can increase the Young’s modulus by up to 2 orders of magnitude and produce mechanically robust structures that can be handled. The self-assembly of the supramolecular photoinitiators is also explored using viscosity and SAXS measurements, providing an understanding of why only **4BPAcFF** successfully forms gel noodles. Our method offers a simple yet effective technique for localizing polymerization, enabling fine-tuning of mechanical properties and the fabrication of intricate designs such as hollow-core structures.

## Graphical abstract

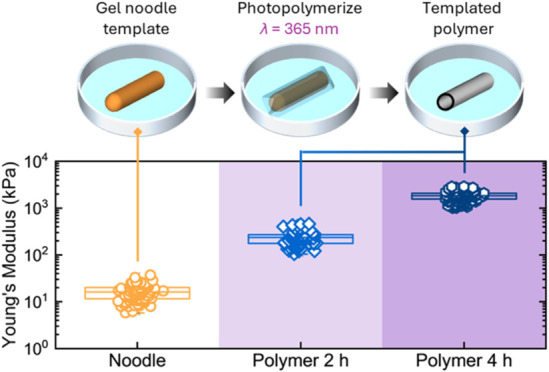

## Introduction

Benzophenone is commonly used as a photoinitiator in processes such as ultraviolet-curing (UV-curing) of coatings, inks, and adhesives.^1,2^ Benzophenone absorbs UV light and undergoes a transition to an excited state, leading to the formation of free radicals.^3^ These radicals can be used to initiate polymerization reactions in acrylates and methacrylates, resulting in rapid curing when exposed to UV light.^4^ As a photoinitiator, benzophenone is particularly useful because it can be activated by a broad range of UV wavelengths and can be combined with co-initiators, such as amines, to enhance its efficiency.^5,6^ Its versatility and strong absorption make it ideal for applications requiring quick, controlled polymerization, such as in three-dimensional (3D) printing, dental materials, and protective coatings.^6–8^

In many cases, benzophenone and related photoinitiators are dissolved or uniformly dispersed throughout the monomers to be polymerized.^6,9^ This leads to well-defined and uniform polymeric films. To spatially resolve photopolymerization, a top-down approach is typically used, employing a computer-controlled light source or photomask to ensure that polymerization occurs only in unmasked areas.^6^ The photomask ensures that only selected areas undergo polymerization, enabling the creation of fine, detailed structures such as microcircuits or 3D patterns; however, this approach can be limited by cost and setup complexity.^10–12^

Another method to achieve spatial resolution is to isolate the benzophenone in specific regions. There are examples where benzophenone has been immobilized on a surface to ensure that the photoinitiation and polymerization occurs from this interface.^13–19^ These surfaces can be flat or can be curved such as from textile fibers. The benzophenone is typically covalently bound to the surface in some way. As an extension to this, examples of initiators that allow gradients to be prepared based on density have also been described.^20–22^

As an alternative approach, here we describe the use of surfactant-like aggregates with a benzophenone core as a means of localizing the photoactive groups (Figure 1). We use dipeptides functionalized at the *N*-terminus with benzophenone. These molecules self-assemble in water to give micellar aggregates. Judicious design enables the formation of worm-like micellar aggregates with a suitable viscosity such that gel noodles can be prepared on cross-linking under extrusion. By incorporating the benzophenone component directly into the gel noodle, our system expands the potential of gel noodles in biomedical applications, including targeted drug delivery and cell manipulation. For instance, spatially controlled radical generation without widespread damage has attracted increasing interest in cancer treatments and cell tagging.^23,24^ These gel noodles can also be used as photoinitiators for polymerizations, spatially locking the initiation sites and leading to localized polymerization from the surface of the gel noodle. The gel noodle can be removed after polymerization, allowing hollow-core structures to be formed from the templates. This work offers a new method for spatially controlling photopolymerization and opens new possibilities for fabricating microfluidic devices that are compatible with cell culturing.^25^

**Figure 1:**
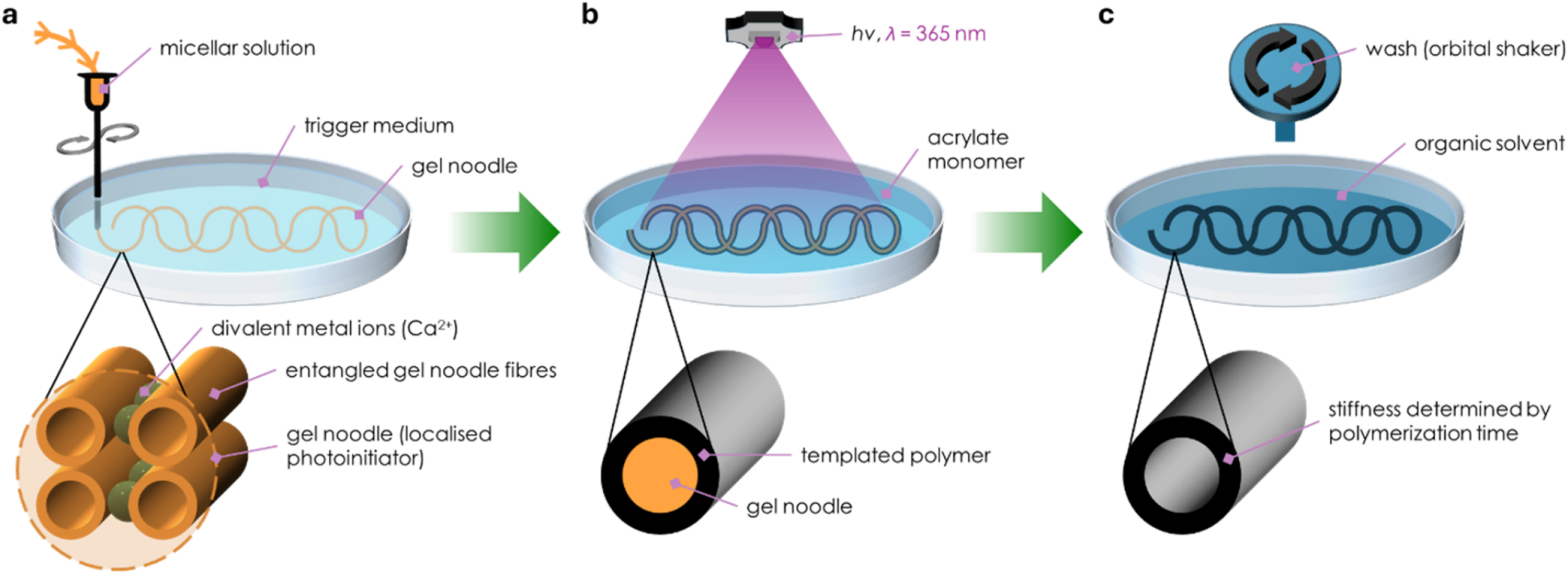
Localized photoinitiators for spatially resolved polymerization. Schematic depicting the process of (a) forming the supramolecular gel noodle template by extruding the photoinitiator-gelator into the trigger medium, (b) polymerizing around the gel noodle template, and (c) removing the excess monomer and gel noodle to give the desired structure.

## Results and Discussion

Dipeptides functionalized at the *N*-terminus with large aromatic groups are known to self-assemble in water.^26–30^ In many cases, micellar structures are formed at high pH. When the molecule is very hydrophobic, it is typical for worm-like micelles or nanotubes to be formed;^31^ less hydrophobic molecules typically self-assemble into ill-defined spherical structures.^32^ A range of different aromatic groups have been added to the *N*-terminus of dipeptides including Fmoc (fluorenylmethoxycarbonyl), naphthalene, phenanthrene, anthraquinone, and benzophenone.^33–35^ In many cases, these aromatic groups are essentially nonfunctional and act simply to provide hydrophobicity and π-interactions to direct self-assembly. However, this does not have to be the case and indeed a benzophenone-functionalized dipeptide has been shown to be photoactive.^35^

Inspired by this, we synthesized three benzophenone-diphenylalanine conjugates: **3BPAcFF**, **3BPFF** and **4BPAcFF** (Figure 2a). We varied the position at which the diphenylalanine is attached to the benzophenone as well as the linker between the benzophenone and dipeptide (see Supporting Information for synthetic pathway and characterization). In all cases, the *C*-terminus is free, meaning that we can use pH to control the self-assembly. To induce self-assembly, an aqueous dispersion at high pH (10.0 ± 0.1, Figure S1) was prepared from each conjugate at a concentration of 5, 10, or 20 mg mL^–1^. At this pH, the carboxylic acid is deprotonated, leading to the formation of micellar aggregates (Figure 1b insert).

**Figure 2:**
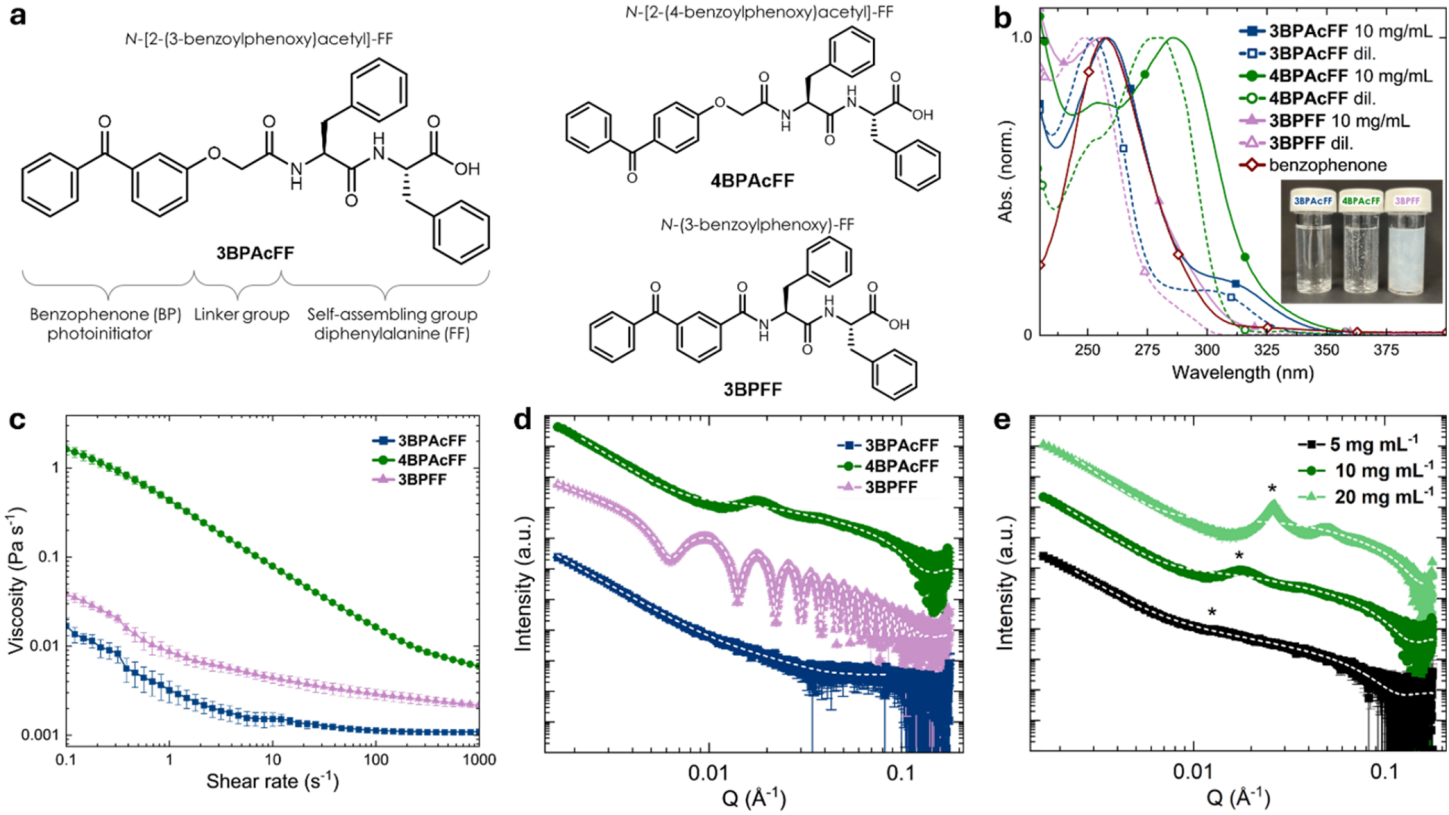
Functionalized dipeptides and their self-assembly in water at pH 10.0 ± 0.1. (a) The three materials investigated in this study with each major component labeled for **3BPAcFF**. Each material encompasses the benzophenone (BP) moiety as a photoinitiator, a diphenylalanine (FF) moiety for self-assembly, while **3BPAcFF** and **4BPAcFF** both have a linker group between the two moieties. (b) UV–vis spectra of the dilute gelators (dil.; <0.01 mg mL^–1^) compared to 10 mg mL^–1^. Inset: photograph of the gelator solutions (pH 10.0, 10 mg mL^–1^). (c) Viscosity data (10 mg mL^–1^). (d) SAXS data obtained at 10 mg mL^–1^ and model fits (white dashed line) comparing systems. (e) SAXS data for **4BPAcFF** at different concentrations including fits (white dashed line).

As their purpose was to be used as photoinitiations, we first recorded their absorption at both optically dilute (<0.01 mg mL^–1^) and micellar concentrations (10 mg mL^–1^), which were used to form the gel noodles (discussed later, Figure 2b). Each dipeptide showed a slight bathochromic shift in peak absorption in the concentrated solution compared to the molecularly dissolved solution, as expected when in the self-assembled state. Nevertheless, the benzophenone component remained evident, allowing us to use UV light to initiate polymerization at micellar concentrations.

The micellar structures formed at a concentration of 10 mg mL^–1^ were probed using viscosity (Figure 2c), small-angle X-ray scattering (SAXS, Figure 2d; fitting given in Tables S1 and S2), and cryo-electron microscopy (cryo-EM, Figure S2). The viscosity of **3BPAcFF** was very low, indicating a small presence of persistent structures in this material. The SAXS data best fits to a combined power law and cylinder model, with the cylinder component needed to account for the slight bump in the data at 0.03 Å^–1^. This further implies a small concentration of wormlike structures present in solution that do not interact by entangling or forming bundles, which accounts for the low viscosity. In comparison, more viscous solutions were formed by **3BPFF**, and shear-thinning behavior was also observed, suggestive of the presence of more wormlike micelles that align under flow. Indeed, the SAXS data are best fitted using a hollow cylinder model, with a radius of 36.8 nm and a thickness of 4.2 nm, representing long fibrillar micelles. These structures were confirmed by the cryo-EM results (Figure S2a), which show significantly larger tube-like structures compared to **3BPAcFF** (Figure S2b). **4BPAcFF** formed more viscous solutions, also accompanied by shear-thinning behavior. In line with the viscosity data, the SAXS data for **4BPAcFF** are best fitted to a combined power law and cylinder model with a significant cylinder component arising from the micellar structures. On top of this, there is a peak at a Q value of 0.02 Å^–1^. Like related systems,^36^ this peak represents the correlation length of the system, ξ, as these systems are behaving as polyelectrolytes. The peak position shows a concentration dependence (Figure 2e) and follows a scaling of ξ ∼ c^1/2^ as expected for wormlike micelles behaving as a polyelectrolyte chain (Figure S3).^37,38^ The cryo-EM images of **4BPAcFF** (Figure S2c,d) show thin fibers of approximately 5 nm in diameter that tend to bundle, leading to the increased viscosity of the sample and accounting for the cylindrical component observed in the SAXS data. As each of the gelator solutions exhibited non-Newtonian, shear-thinning behavior, the data were fitted using standard Carreau–Yasuda and Cross models (Figure S4). **3PBFF** and **4BPAcFF** were best described by the Carreau–Yasuda model, with both residuals obeying normality. For **3BPAcFF**, the residual from the Cross model did not pass Shapiro–Wilk or Anderson–Darling normality tests; however, the residuals appeared approximately normal, the visual fit was good, and the resulting rheological parameters were reasonable given that the sample showed a very low viscosity. Together, this confirmed that altering the chemical structure of the linker group between the benzophenone and the self-assembling moiety led to distinct micellar architectures, which in turn, drove the observed differences in viscosity.

We confirmed that each of the gelators could produce self-supporting bulk gels when triggered with calcium chloride (Figure S5). However, our intention here was to prepare localized photoinitiators. To prepare these, we used a technique developed by Stupp’s group, forming so-called supramolecular gel noodles.^39^ To form gel noodles, a viscous solution is required which is extruded into a cross-linking bath, herein an aqueous calcium chloride solution (0.5 M). We have previously described how this method can be manipulated to form long (multimeter) gel noodles with controlled diameters.^40^ Using this approach, unsurprisingly considering the viscosity data described in Figure 2c, only **4BPAcFF** was found to form well-defined gel noodles when extruded into a bath containing the calcium salt cross-linker at a minimum concentration of 10 mg mL^–1^ (Figure 3a). **3BPAcFF** was found to give gel noodles that were extremely fragile such that they were unable to be lifted from the cross-linking bath. **3BPFF** did not produce gel noodles, but produced randomly sized precipitates that did not interlock and were also unable to be removed (Figure S6).

**Figure 3:**
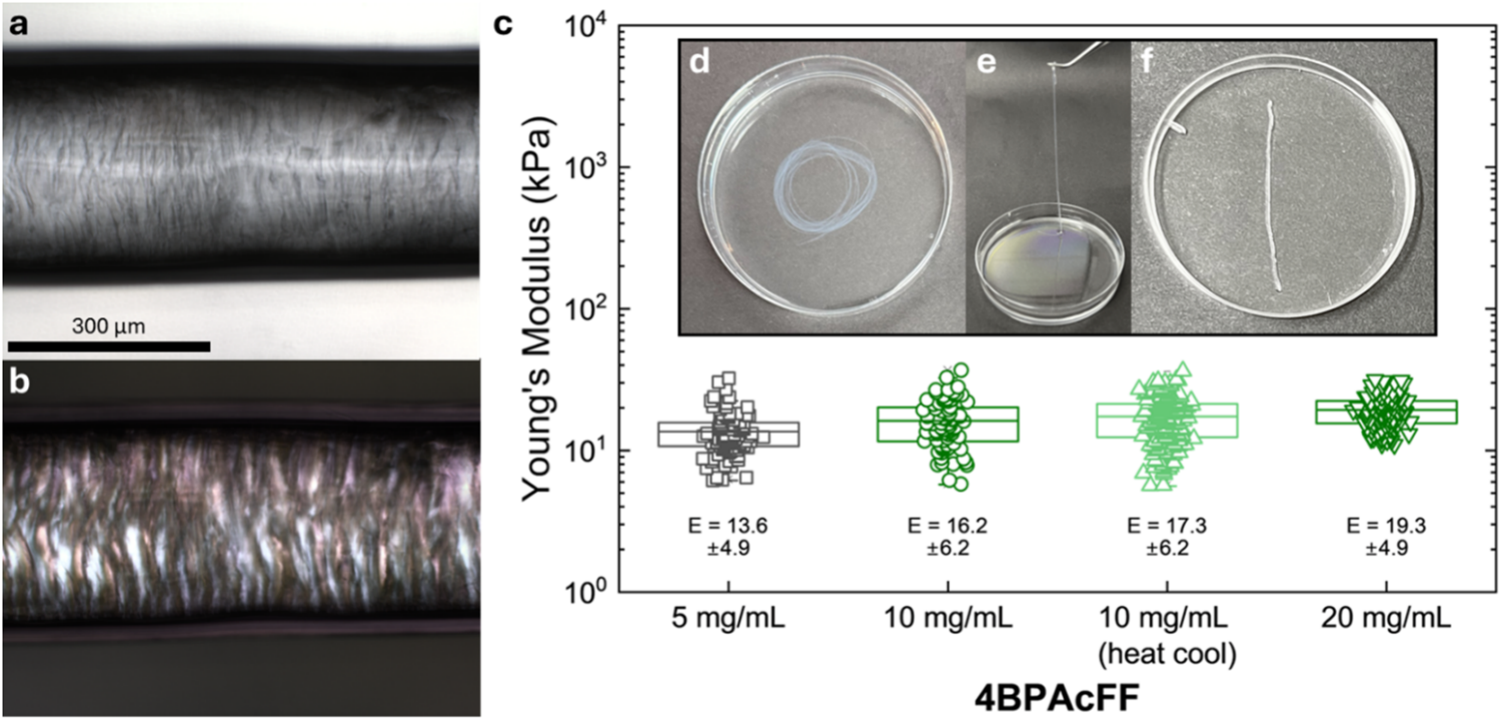
**4BPAcFF** supramolecular gel noodles. (a) A section of the gel noodle viewed under the microscope and (b) when using cross-polarized light. (c) Statistical bar plot of nanoindentation data for **4BPAcFF** with the average and standard deviation given adjacent. Each point on the graph represents the local Young’s modulus measured at a specific position on the gel noodle. Photographs of the gel noodles (d) as prepared, (e) lifting a section of the gel noodle, and (f) an exemplar piece of gel noodle used for nanoindentation measurements. The Petri dish is 90 mm in diameter.

The gel noodles prepared from **4BPAcFF** were typically 500 μm in diameter (as determined by the extrusion needle, Figure 3). Considering the formation process, to assess if there was a general alignment along the gel noodle backbone, the gel noodles were first viewed under a microscope with polarized light (Figure 3a,b). At 10 mg mL^–1^, the gel noodles showed some birefringent behavior, indicating a degree of alignment that is mimicked by the increased viscosity of **4BPAcFF**. However, increasing the concentration of the pregelation solution to up 50 mg mL^–1^ did not result in any significant changes in the microscope imaging (Figure S7).

The mechanical properties of the **4BPAcFF** gel noodles were then measured. To do this, we evaluated the Young’s modulus (E) of the gel noodles by nanoindentation.^41^ This allows us to quantify the local mechanical properties at micron length scales. The Young’s modulus is improved slightly as the concentration of the **4BPAcFF** increased (Figure 3c, see Table S3 for *p*-values). We attempted to strengthen the gel noodles by subjecting the high pH solutions to heat–cool cycles at 70 °C pregelation; this has been shown to be effective for other bulk gel systems.^42^ However, in this case, this provided minimal benefit to the gel noodles in terms of their useability and mechanical strength. We therefore focused on **4BPAcFF** at 10 mg mL^–1^, the lowest concentration that can be easily manipulated by hand. Additionally, gel noodles formed from **4BPAcFF** were found to be stable, with no changes in appearance or mechanical properties after aging for 7 days. Soaking the gel noodles in either the trigger solution (aqueous calcium chloride, 0.5 M) or in water (to prevent salt precipitation) had no visible effect (Figure S8), and the Young’s modulus remained unchanged (Figure S9).

Next, we investigated the use of **4BPAcFF** as a photoinitiator. We selected a commercial 365 nm LED as our irradiation source, and although the absorption of the gelator and micellar solutions was relatively low at this wavelength, it was still sufficient to induce polymerization.^35^ A mixture of **4BPAcFF**, a cross-linkable acrylate monomer [pentaerythritol tetraacrylate (PETA); in a 1:4 ratio, respectively], and DMSO-*d*_6_ solvent was irradiated in an NMR tube. Visually, it was clear that the polymerization proceeded after 5 min of irradiation, with a change in appearance from transparent to translucent (Figure S10). Both the gelator and PETA signals broadened significantly (indicating successful polymerization), and the ratio of terminal alkene groups was reduced by approximately 20-fold after 30 min of irradiation. Additionally, we did not see any dimerization of the benzophenone component or significant degradation after irradiation in DMSO-*d*_6_, or after irradiating a piece of gel noodle for 1 h (and freeze-drying to allow for NMR, Figure S11). Electron paramagnetic resonance spectroscopy was used to confirm radical generation from the benzophenone component (Figure S12). Radical adducts were observed in irradiated reaction mixtures containing spin trap 5,5-dimethyl-1-pyrroline *N*-oxide (DMPO). The hyperfine values of the adducts (PETA: *a*_N_ = 14.1 G, *a*_H_ = 21.2 G and 2-hydroxyethyl acrylate (HEA, discussed later): *a*_N_ = 14.5 G, *a*_H_ = 21.5 G) are consistent with trapping of carbon-centered radicals.^43^ Selective broadening of the high field line suggests that the trapped radicals are macromolecules, i.e., polymer chain radicals. This effect is observed for both monomers but is significantly more pronounced for the cross-linkable PETA sample. Finally, we compared the photopolymerization rates between PETA and another water-soluble acrylate monomer, HEA, using **4BPAcFF** as a photoinitiator at 1 wt %. We attempted to use gel permeation chromatography to describe the polymer materials; however, neither were soluble in tetrahydrofuran or *N*,*N*-dimethylformaldehyde (instrument-compatible solvents), even at concentrations less than 1 mg mL^–1^. As an alternative, we used Fourier-Transform infrared spectroscopy (FT-IR) to monitor the reduction of the double bond signal between 791–834 cm^–1^, following a previously described method (Figure S13).^44^ Both acrylate signals from PETA and HEA decreased rapidly, taking approximately 30 and 70 s, respectively, to reach over 90% conversion. These results were comparable with other photoinitiators (average 80% in 20 s) based on benzophenone, ketocoumarin, and thioxanthone moieties.^44–47^ With this confirmation that the benzophenone component of **4BPAcFF** can still act as an efficient photoinitiator, we then sought to combine the two techniques.

Localized polymerization was achieved using two distinct methods (see Figure S14 for schematic). First, the gel noodles were removed from the trigger medium and manually cut and arranged into the desired shape (here, a rod). The gel noodle template was then encased in the PETA monomer (solvent free), and we found that its low polarity and hydrophobicity did not dissolve the template. To ensure a self-supporting polymer was formed, we irradiated the template/monomer mixture for either 2 or 4 h. The excess monomer, gel noodle template, and loose polymer aggregates were washed away with solvent (see Photopolymerization) leaving the templated polymer (Figure 4a). Again, we viewed the newly formed structures under the microscope and when using polarized light. There was a distinct change in appearance from translucent to opaque, and on average, the gel noodles shrank to approximately 300 μm after solvent washing. The average thickness of the structures increased with irradiation time, reaching approximately 300, 360, 410, 450, 500, and 500 μm after 2 to 6 h, respectively, at which point it appeared to plateau. There was no general alignment visible in the cross-polarized light, however, the polymer structure was clearly localized to only where the gel noodle was positioned. Most interestingly, the Young’s Modulus increased significantly from ≈16 to ≈240 kPa after 2 h, which continued to increase to ≈1840 kPa after 4 h. This increase in stiffness can be ascribed to the cross-linked coating gradually forming a shell around the template. Scanning electron microscopy (SEM) showed that the surface of the 2 h polymer structure was very rough and somewhat fibrous (Figure S15a), which is consistent with the uncontrolled nature of the photopolymerization on the surface of the gel noodle. However, in the 4 h polymer structure, the surface roughness was reduced, and the overall structure appeared to have a higher cross-linking density. This filling and smoothing of the structure explain the increase in Young’s modulus, as the polymers became increasingly stiffer with increasing photopolymerization time (Figure S15b).

**Figure 4:**
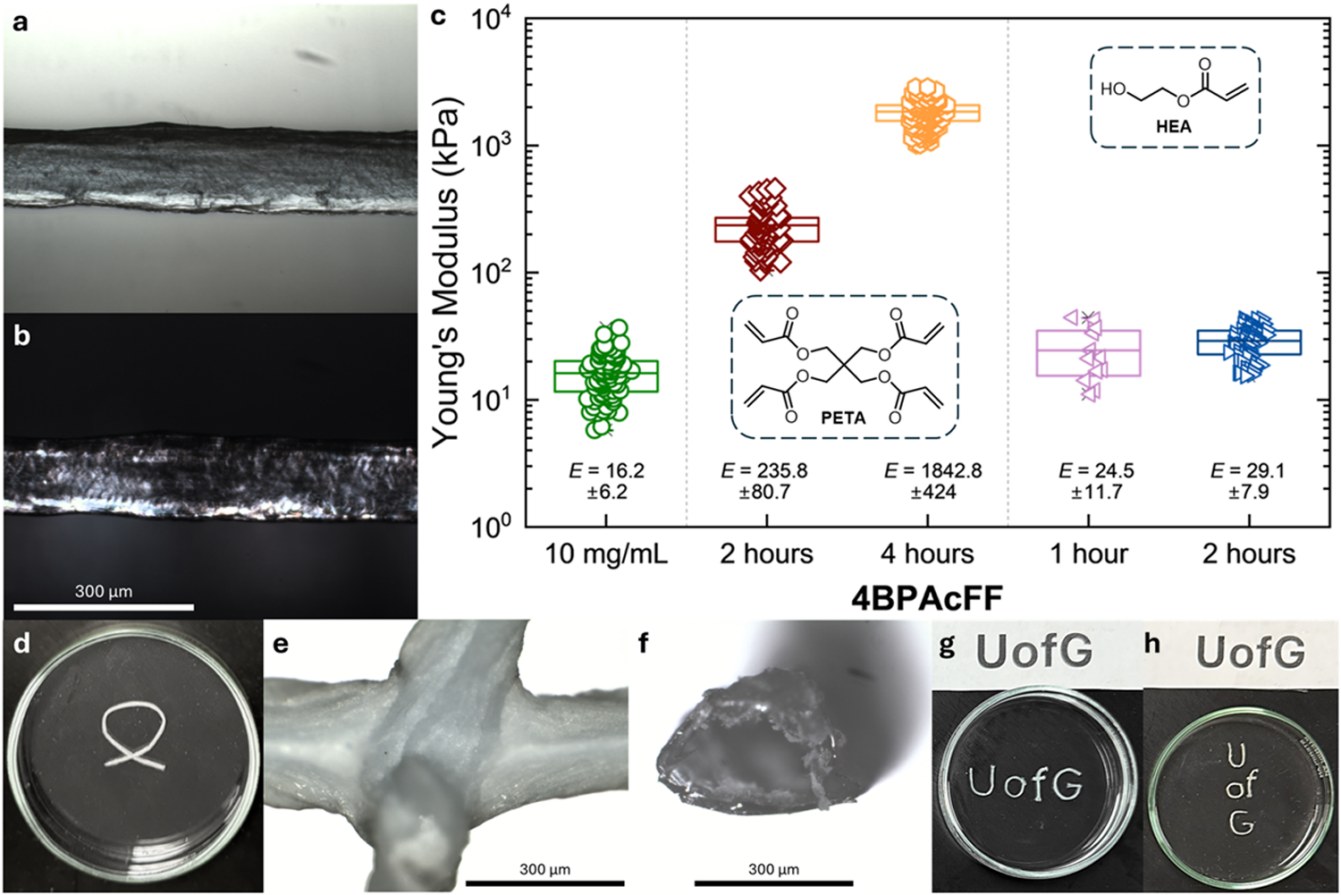
Supramolecular gel noodle templating for localized polymerization. (a) A section of the polymer viewed under the microscope and (b) using cross-polarized light. (c) Statistical bar plot of nanoindentation data for **4BPAcFF**, compared to the two polymers (PETA or HEA) at different irradiation times with the average and standard deviation given adjacent. Each point on the graph represents the local Young’s modulus measured at a specific position on the gel noodle. Inset: the monomer used for the photopolymerization. (d) Photographs of a polymerized bow-tie shape. Microscope images of (e) the intersection between two polymer tracks and (f) a cross-section from the end, which appears hollow. Photographs of the template ‘UofG’ (g) immediately after polymerization and (h) after solvent washing and rearrangement, with the stencil text above.

For the second method, it was found that gel noodles could still be formed when the trigger solution was mixed with a water-soluble acrylate monomer, 2-hydroxyethyl acrylate (HEA). This enabled formation of the gel noodle template in a single step, eliminating the need for manual templating afterward, and could potentially be compatible with applications that can produce even more complex structures using 3D, gel-based, or rapid liquid printing. In a 1:4 ratio (monomer to calcium chloride, by volume) the gel noodles were still strong enough to be manually lifted from the solution, although they had to be polymerized quickly, as the gel noodles would dissolve into randomly sized pieces and could no longer be lifted after approximately 1 h. Additionally, the polymerization speed needed to be increased to account for their solubility in the mixture solution. Polymerization was achieved by irradiating the gel noodle with 365 nm LEDs positioned above and below the sample, at a closer distance to increase the flux to approximately 120 mW cm^–2^. The newly templated polymers were softer and more flexible, compared to the PETA polymers which were brittle and more rigid. The gel noodles also typically swelled during the polymerization process, producing less defined structures that did not fully mimic the original gel noodle template (Figure S16). We again viewed the structures under the microscope using cross-polarized light, which only showed some birefringent behavior; however, there was still a visible change from translucent to opaque. After solvent washing, the gel noodles shrunk to around 200 μm, but unlike the PETA structures, these could be reswollen to over 500 μm when sat in water (Figure S16e,f), without losing their generalized ‘rod-templated’ shape. The mechanical strength of the gel noodles was also investigated by measuring the Young’s modulus by nanoindentation. This again increased from the original gel noodle to ≈25 kPa (from ≈16 kPa) after 1 h of irradiation and continued to increase slightly to ≈29 kPa after 2 h, although this was within the experimental error. These results were consistent with the use of the HEA monoacrylate as the monomer, compared to the cross-linkable PETA, which produced stiffer structures (combined nanoindentation data given in Figure S17). The SEM images of the HEA-based structures differed considerably from those of the PETA-based polymers (Figure S15c,d). Both the 1 and 2 h structures appeared similarly smooth and uniform, aligning with the minimal increase in the Young’s modulus observed between samples, and the fact that they were prepared in a mixture of trigger solution and monomer. Photopolymerization occurs uniformly throughout the gel noodle template, as the monomer is already dispersed within the structure. Altogether, this demonstrates the ability to strengthen the functionalized gel noodles through selective polymerization, enabling the tuning of mechanical and physical properties.

Finally, we showcased how the gel noodles can sculpt more intricate shapes that localize the subsequent photopolymerization. To accommodate heating effects from prolonged irradiation, the polymerization conditions with the PETA monomer were slightly adjusted (see Photopolymerization). The first structure was a bow-tie shape featuring an intersection, where two pieces of gel noodle overlapped. After solvent washing, the newly formed polymer was strong enough to be manually handled, and when viewed under the microscope (Figure 4e), it revealed the fusing of the intersection into a single piece. A cross-section of the polymer at the edge of the structure (Figure 4f) appeared hollow, with only an outer shell of polymer remaining in place of the gel noodle template. The approximate diameter of the shell was 300 μm, which matched the diameter of the gel noodle template in the top-down view seen in Figure 4a and was consistent with the observed shrinkage after solvent washing (from ≈500 μm). Finally, we demonstrated how a shape could be maintained by arranging the gel noodles into a desired structure. The letters ‘UofG’ were manually arranged using a text template and retained their shape immediately after irradiation (Figure 4g). Following solvent washing, the letters could be handled and rearranged while maintaining their intended form (Figure 4h).

## Conclusions

In conclusion, functionalized supramolecular gel noodles can be used as templates for spatially resolved photopolymerization. We have shown that new photoinitiators can be produced and tailored to retain the self-assembly gelation properties of micellar dipeptides. Furthermore, by modifying the linker group between the benzophenone-based photoinitiator component and the dipeptide component, the viscosity can be sufficiently increased to enable supramolecular gel noodle formation. The gel noodles are robust enough to be manually handled for pattern templating, and their mechanical properties remain unaffected after annealing their micellar solutions or aging up to 7 days. Photopolymerization of a cross-linkable acrylate monomer around the shell of the gel noodle can yield structures that are rigid and exhibit significantly increased mechanical strength. Alternatively, monoacrylate monomers can be used to produce softer yet still self-supporting structures that do not dissolve in common organic solvents. Finally, we have also shown that our system of templating can be used to create more structurally complex materials. By tailoring the polymerization time to suit the monomer, we can produce shapes containing details that are difficult to mimic without complex instrumentation or experimental setups, such as hollow tubes and sections that intersect.

## Materials and Methods

### Materials and General Experimental

The reagents used, the synthetic route to each of the gleators, their corresponding characterization data, including comparison of the synthetic materials’ thermal properties (Figure S18) and FI-TR spectra (Figure S19), and the instruments and general experimental procedures are given in the Supporting Information.

### Bulk Gel and Gel Noodle Formation

The required amount of each gelator was weighted into a vial with a stirrer bar. The required amount of DI water was added, followed by 1 equiv of 0.1 M sodium hydroxide to give a final concentration of 5, 10, or 20 mg mL^–1^. For example, to make 10 mL of **4BPAcFF** at 10 mg mL^–1^: 100 mg of **4BPAcFF** was weighed into a 14 mL glass vial (Sanco, T103/V3) with a magnetic stirrer bar (8 mm × 1.5 mm). This was first diluted with 8.184 mL of deionized water, followed by 1.816 mL of 0.1 M aqueous sodium hydroxide. Vials were sealed and stirred at 1000 rpm for a minimum of 16 h. The solutions were pH adjusted to 10.0 ± 0.1 via the addition of small aliquots (≈2 μL) of either aqueous hydrochloric acid (1 M) or sodium hydroxide (1 M). During the pH adjustment, vials were stirred after addition, and the more viscous samples (mainly **4BPAcFF**) were additionally mixed using a vortex mixer in 20 s bursts. For the bulk gels, 1.0 mL of the solution was transferred into a 7 mL Sterilin vial (Thermo Fisher, 129A). Over this, a 1.0 mL aqueous solution of calcium chloride is carefully added at a molar ratio of 1:2 between the gelator and the calcium chloride. The vial was left undisturbed to gel overnight (minimum of 16 h) before testing. For the gel noodles, pH adjusted gelator solutions were taken up into a 10 mL plastic syringe (BD Plastipak, 305959) connected to a 50 mm × 4 mm piece of connecting tubing attached to a 6 mm, 18-gauge flat cut needle. The gelator solution was extruded into a Petri dish filled with trigger solution (either aqueous calcium chloride 0.5 M or aqueous calcium chloride 0.5 M and 2-hydroxyethyl acrylate, 4:1 by volume) using a syringe pump set to express 3 mL min^–1^. The Petri dish was spun on a spin coater (Ossila) at 100 rpm and the gelator extruded near the outer edge of the dish. For the complex shapes a gelator solution of 20 mg mL^–1^ was used, along with a higher flow rate on the syringe pump of 6 mL min^–1^. All gelator solutions were pH adjusted (10.0 ± 0.1) prior to gel noodle formation and used for a maximum of 1 week.

### Nanoindentation

Nanoindentation measurements were performed using a Chiaro nanoindentation device (Optics11, NL) following a previously described protocol at room temperature (approximately 22 °C).^41^ A probe of stiffness (*k*) 0.48 N·m^–1^ was used for the measurements, except for the PETA polymerized for 4 h, for which the soft probe was found to be unsuitable and a stiffer probe of *k* = 4.18 N·m^–1^ was used. The radius (*R*) of the spherical tip attached to the cantilever was 3 μm for both probes. The nanoindenter was mounted on top of an inverted Zeiss Axiovert 200 M microscope. Approximately 3 cm of a gel noodle or polymerized material was cut with scissors and placed in a glass Petri dish. A metal washer was kept on the top of the sample to prevent movement. Deionized water was added to fully immerse the sample and prevent drying. For the 2-hydroxyethyl acrylate (HEA) materials, the resulting polymer would swell significantly upon immersing in water, thus, measurements were carried out under the same conditions but were immersed in ethanol. The Petri dish was placed on the microscope stage, keeping the sample along the *x* direction. At least two matrix scans were performed on each sample, and each matrix scan consisted of 25 indentations. The spacing between subsequent indentations was 6 μm. For data analysis, the forward segment of the collected force–displacement (*F*–*z*) curves were analyzed using a custom open-source software.^48^ The contact point was identified by a goodness of fit algorithm^48^ to convert *F*–*z* curves into force–indentation (*F*–δ) curves. The *F*–δ curves were analyzed up to a maximum indentation of δ = 1 μm = 0.1*R*, which is indeed much smaller than the thickness of the gel noodle, typically in the range of 100 μm. This regime justifies fitting the data with the Hertz model for the indentation of a rigid sphere over a half plane and ignoring any contribution of the bottom rigid substrate. The average force–indentation curves can be viewed in Figure SX.

### SAXS

Small-angle X-ray scattering (SAXS) measurements were performed at the Diamond Light Source, Didcot, U.K., at the I22 beamline under experiment number SM35286–1. The beamline operated at an energy of 12.4 keV and the detector distance was set to 9.750 m, allowing a final *Q* range of 0.0016 to 0.177 Å^–1^. Gelator solution samples were prepared as described previously (at pH 10.0 ± 0.1) and transferred into borosilicate glass capillaries (1.55 mm internal diameter) using a 1 mL syringe fitted with an 80 mm 21G needle. For all samples, 10 × 100 ms frames were collected and averaged. The raw data was processed using the Dawn Science software (version 2.27),^48^ according to a standard I22 pipeline.^49^ As part of the processing, the backgrounds were subtracted from the raw two-dimensional (2D) SAXS data and a full azimuthal integration was performed to reduce the data to an *I* vs *q* plot. The plots were then fitted to structural models in the SasView software (version 5.0.4). For the data presented in Figure 2e, fitting the *Q* peaks was only possible using a structure factor relating to hard spheres. As this was not representative of the micellar system presented in this work, the data was instead fit to a cylinder model and the peaks were used to investigate the correlation length of the system.

### Cryo-EM

Vitrification of the gelator samples for cryo-EM was conducted by taking 3 μL gelator suspensions and applying them on glow discharged lacey carbon grids. Excessive sample was blotted away by blotting from both sides of the grid, leaving a thin film of sample on the grid, which was plunge frozen in liquid ethane using a Vitrobot Mark IV (Thermo Fisher Scientific). The **4BPAcFF** gelator solution (10 mg mL^–1^; pH 10.0 ± 0.1) was required to be diluted 8× due to its high viscosity to ensure it formed imageable ice. Cryo-EM images were collected on a 200 keV electron microscope (Glacios, Thermo Fisher Scientific) with pixel size of ≈1.2 Å.

### Photopolymerization

For the PETA monomer photopolymerizations, a piece of a gel noodle was cut to the required size and placed in a dry Petri dish. The gel noodle was immersed in deionized water for approximately 1 min, then the water decanted off. This process was repeated three times to remove any excess trigger solution before the gel noodle was transferred to a clean Petri dish and manually arranged into the desired shape using tweezers. The gel noodle was then immersed in the PETA monomer (no solvent), positioned under a commercial 365 nm LED (LedEngin, 829–0841) mounted to a heat sink to give a power density of approximately 100 mW cm^–2^, and irradiated for the specified time (as indicated or 6 h for the complex shapes). For the HEA monomer photopolymerizations, as the gel noodle was formed in the trigger medium:monomer mixture, the gel noodle was cut and arranged into the desired shape within the solution. The excess gel noodle was removed and the samples placed under the LED that was brought closer to give a power density of approximately 120 mW cm^–2^ and irradiated for the specified time. For all samples after irradiation, the excess solution/monomer was removed by gently flowing acetone over the sample (with a wash bottle) until only the polymer was evident in the Petri dish. This was then immersed in acetone, covered with another Petri dish, and left to wash overnight on an orbital shaker (Ika, VXR basic Vibrax, 100 rpm). Finally, the solution was decanted off to give the polymer, which could be removed by detaching from the Petri dish using tweezers.

### Photopolymerization Rates

Fourier-Transform infrared spectroscopy (FT-IR) was used to monitor the photopolymerizaiton. A stock solution (**4BPAcFF**, 1 wt %; monomer, 200 mg; tetrahydrofuran, 2.0 mL) was dropped onto a transparent KBr salt IR window. A second window was used to encase the liquid and the excess wiped away. The double bond conversion (DC %) was compared by taking the ratio of peak area of the peak between 791–834 cm^–1^ at the specified times during 365 nm LED irradiation (≈100 mW cm^–1^) following a previously reported method.^44^

### EPR

EPR spectra were recorded at X-band (9.4 GHz) on a JEOL JES-X320 spectrometer in glass capillaries (0.8 mm ID). Typical acquisition parameters: microwave power 1 mW, modulation frequency 100 kHz, modulation amplitude 1 G, time constant 0.03 s. Spin trap 5,5-dimethyl-1-pyrroline-*N*-oxide (DMPO) was added to reaction mixtures immediately prior to spectra acquisition to a 100 mM final concentration. For experiments with irradiation, the 365 nm LED was placed at the bottom of the EPR cavity ≈4 cm below the center of the cavity.

## Supplementary Material



## Notes

General experimental and materials used throughout; the synthetic pathway to the gelators (Scheme S1); photographs of the **4BPAcFF** micellar solution (Figure S1); SEM results for the gelator solutions (Figure S2); graph of Q* against concentration (Figure S3); viscosity data fits (Figure S4); strain and frequency sweeps of the bulk gels (Figure S5); photographs of the gel noodles at different concentrations (Figure S6); microscope images of **4BPAcFF** at different concentrations (Figure S7); photographs and microscope images of aged **4BPAcFF** gel noodles (Figure S8); nanoindentation data for aged **4BPAcFF** gel noodles (Figure S9); NMR of a **4BPAcFF** and PETA mixture during irradiation (Figure S10); NMR of a **4BPAcFF** gel noodle mixture after irradiation (Figure S11); EPR spectra for **4BPAcFF** with irradiation (Figure S12); polymerization kinetics taken from FT-IR (Figures S13); schematic of the polymerization process (Figure S14); SEM images of the PETA and HEA polymer structures (Figure S15); photographs and microscope images of the HEA-based structures (Figure S16); force–indentation curves for **4BPAcFF** (Figure S17); thermal properties of the gelators (Figure S18); FT-IR of the gelators and intermediates (Figure S19); fitting parameters for SAXS (Tables S1 and S2); two-sample *t*-tests of the **4BPAcFF** gel noodles (Table S3); NMR spectra (Figures S20–43) (PDF)
