# Supplementary material for: Supramolecular Benzophenone-Based Photoinitiator for Spatially-Resolved Polymerization
Source: ACS Appl Mater Interfaces. 2025 May 12;17(20):30297–305. doi: 10.1021/acsami.5c03506 (PMC12100643; doi:10.1021/acsami.5c03506)
Supplement: Supplementary file 1 [file am5c03506_si_001.pdf]

Supporting Information

A Supramolecular Benzophenone-Based Photoinitiator for Spatially-Resolved Polymerization

Alex S. Loch,[1] Ibram Mikhail,[1] Simona Bianco,[1] Dipankar Ghosh,[1] Ravi R. Sonani,[2] Victor Chechik,[3] Massimo Vassalli,[4] Edward H. Egelman,[2] Andrew J. Smith,[5] and Dave J. Adams[1,]*

[1] School of Chemistry, University of Glasgow, Glasgow G12 8QQ, UK

[2] Department of Biochemistry and Molecular Genetics, University of Virginia, Charlottesville, VA 22903, USA

[3] Department of Chemistry, University of York, York YO10 5DD, UK.

[4] Centre for the Cellular Microenvironment, University of Glasgow, Glasgow G12 8LT, UK

[5] Diamond Light Source Ltd., Diamond House, Harwell Science and Innovation Campus, Didcot, Oxfordshire OX11 0DE, UK

* Email: dave.adams@glasgow.ac.uk

General Experimental

Thin layer chromatography (TLC) was performed using aluminum backed silica gel 60 F_{254} plates. Column chromatography was performed using Merck silica gel, 60 Å 230–400 mesh. Where solvent mixtures are used, the proportions are given by volume. ^{1}H and ^{13}C NMR spectra were recorded on a Bruker Avance^{III} 400 MHz spectrometer. Chemical shifts (δ) are reported in parts per million (ppm) to the residual solvent peak ($CDCl_3$: 7.26 ppm for ^{1}H and 77.0 ppm for ^{13}C; DMSO-d_6: 2.50 ppm for ^{1}H and 39.52 ppm for ^{13}C). Coupling constants (*J*) are given to the nearest 0.1 Hertz (Hz). Peak multiplicities are reported as singlet (s), doublet (d), triplet (t), multiplet (m), and/or broad (b). Peak assignments are reported as: H_{Ar} = aromatic H, C_{Ar} = aromatic C. High resolution accurate mass measurements (HRMS) were performed on an Agilent 6546 LC/QTOF (quadrupole-time of flight) instrument with an Agilent ESI or APCI source. Elemental analysis was performed on an Exeter CE-440 Elemental Analyser. Infrared absorption spectra were recorded on an Agilent Cary 630 FTIR spectrometer as neat samples using an ATR attachment. Differential scanning calorimetry (DSC) was performed on a Netzsch DSC 214 Polyma. Thermal gravimetric analysis (TGA) was carried out on a Netzsch TG 209 Tarsus and are corrected for the crucible. Melting points (mp) were measured in a glass capillary using a Stuart SMP50 automatic melting point apparatus and are uncorrected. Absorption spectra were measured on an Agilent Cary 60 UV–vis spectrophotometer either dissolved in UV-grade organic solvent or deionized water (Avidity Pico10T2). Microscope images were taken on a Nikon Eclipse LV100ND with a 5× magnification and polarized light at 90° using an Infinity 2.1 digital microscope camera. Bulk gel rheological experiments were carried out on an Anton Paar Physica MCR 101 rheometer. The measurements were taken with a cup and vane geometry (T10-4 V-8.8/97.5-SN42404) at a measuring distance of 2.1 mm. The measurements were taken at a constant temperature of 25 °C. Strain sweeps were performed over 0.01% – 1000% strain at a frequency of 10 rad s^{-1}. Frequency sweeps were taken at 0.5% strain while increasing the frequency from 1 rad s^{-1} – 100 rad s^{-1}. Viscosity experiments were carried out an Anton Parr Physica MCR 302e rheometer. The measurements were taken with a cone plate (50 mm) at a gap height of 0.101 mm and the temperature-controlled bottom plate kept at 25 °C. Scanning electron microscopy (SEM) samples were placed on a conductive

carbon adhesive and gold-coated in a Polaron SC7640 sputter coater before being examined in a TESCAN CLARA.

Materials

(3-Hydroxyphenyl)(phenyl)methanone (F217772), *tert*-butyl 2-bromoacetate (F043685), isobutyl chloroformate (F046752), trifluoroacetic acid (F001271), calcium chloride (F044727), and sodium hydroxide (F545150) were purchased from Fluorochem. 3-Benzoylbenzoic acid (B25519.06), 4-methylmorpholine (127155000), and lithium hydroxide (379181000) were purchased from Thermo Fisher Scientific. (4-Hydroxyphenyl)(phenyl)methanone (A15096) and potassium carbonate (A11662) were purchased from Alfa Aesar. Pentaerythritol tetraacrylate (PETA, 408263) and 2-hydroxethyl acrylate (HEA, 292818) were purchased from Merck. All reagents were used as received.

Synthesis

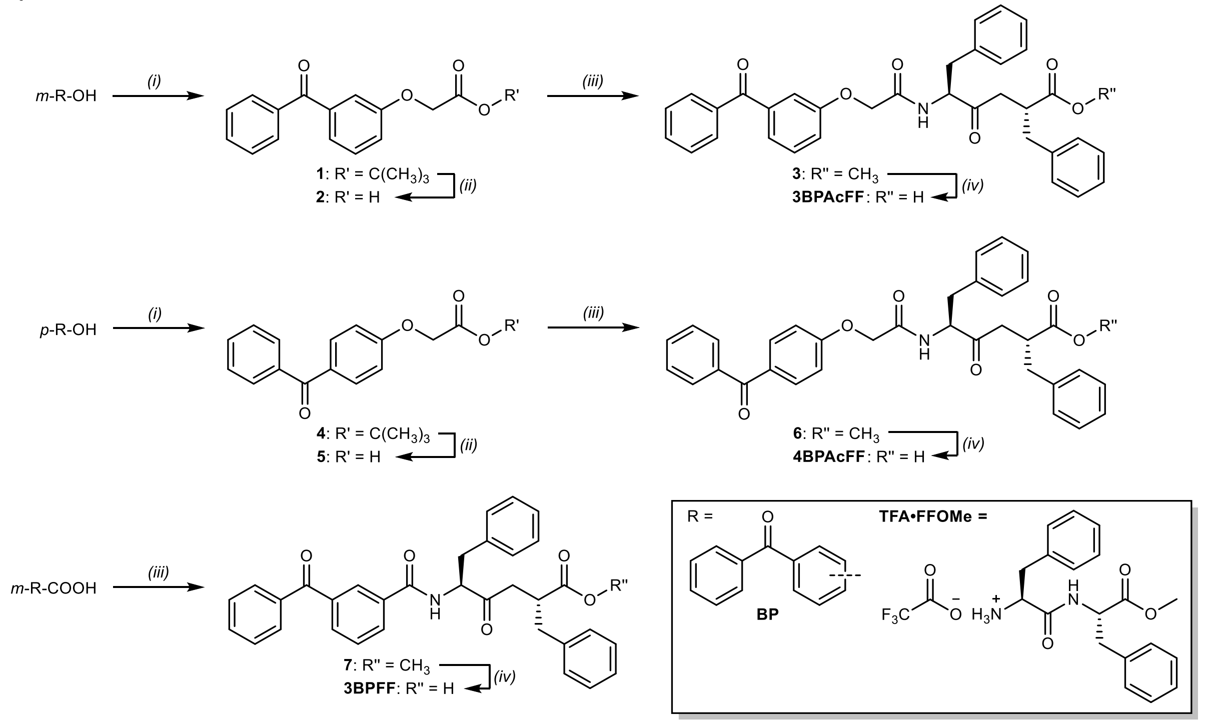

Scheme S1. Synthetic pathway to each of the gelators. Conditions and reagents: *(i) tert*-butyl 2-bromoacetate, potassium carbonate, acetone, reflux, O/N; *(ii)* trifluoroacetic acid, chloroform, O/N; *(iii)* isobutyl chloroformate, 4-methylmorpholine, chloroform, 0 °C, 1 h, then **TFA•FFOMe**, 4-methylmorpholine, 0 °C to RT, O/N; *(iv)* lithium hydroxide, water, tetrahydrofuran, O/N.

tert-Butyl 2-(3-benzoylphenoxy)acetate – **1**

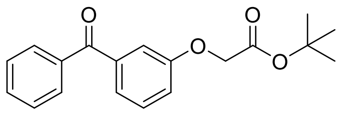

1

A mixture of (3-hydroxyphenyl)(phenyl)methanone (5.0 g, 25.2 mmol), *tert*-butyl 2-bromoacetate (9.80 g, 50.2 mmol), and potassium carbonate (10.0 g, 72.4 mmol) in acetone (200 mL) was refluxed overnight. The solution was allowed to cool to room temperature, filtered, and the solid washed with acetone (100 mL). The filtrate was collected, and the solvent removed to give a colored oil. The oil was diluted in diethyl ether (100 mL) and hydrochloric acid (1M aq., 100 mL). The layers were separated, and the organic layer was washed with water (2 × 100 mL), brine (100 mL), dried over anhydrous sodium sulfate, filtered on a silica plug that was washed with dichloromethane (*c.a.* 100 mL), and the solvent removed to give a colorless oil. The crude oil was purified by column chromatography over silica using dichloromethane as eluent to afford **1** as a colourless oil after removal of the solvent (6.61 g, 84%). IR (oil) ν/cm^{-1}: 1749 (C=O). UV–vis: λ_{max}(acetonitrile)/nm: 216 (log ε/dm^3 mol^{-1} cm^{-1},

4.36), 252 (4.13), 302 (3.35). ^{1}H NMR (400 MHz, $CDCl_3$) δ: 7.81–7.77 (2H, m, $\underline{H}_{Ar}$), 7.61–7.56 (1H, m, $\underline{H}_{Ar}$), 7.47 (2H, t, J 7.5, $\underline{H}_{Ar}$), 7.42–7.36 (2H, m, $\underline{H}_{Ar}$), 7.32 (1H, d, J 3.1, $\underline{H}_{Ar}$), 7.18–7.13 (1H, m, $\underline{H}_{Ar}$), 4.56 (2H, s, $C\underline{H}_2$), 1.48 (9H, s, $C\underline{H}_3$). ^{13}C NMR (101 MHz, $CDCl_3$) δ: 196.3 ($\underline{C}$=O_{BP}), 167.8 (CH_2-$\underline{C}$=O), 158.0 (O-$\underline{C}_{Ar}$), 139.0 ($\underline{C}_{Ar}$), 137.6 ($\underline{C}_{Ar}$), 132.6 ($\underline{C}H_{Ar}$), 130.1 ($\underline{C}H_{Ar}$), 129.5 ($\underline{C}H_{Ar}$), 128.4 ($\underline{C}H_{Ar}$), 123.8 ($\underline{C}H_{Ar}$), 119.7 ($\underline{C}H_{Ar}$), 115.2 ($\underline{C}H_{Ar}$), 82.7 ($\underline{C}$-CH_3), 65.9 ($\underline{C}H_2$), 28.2 ($\underline{C}H_3$). HRMS (ESI/QTOF) *m/z*: $[M+Na]^+$ calcd for $C_{19}H_{20}NaO_4$ 335.1254 (100%), 336.1288 (21%); found 335.1253 (100%), 336.1286 (22%).

2-(3-Benzoylphenoxy)acetic acid – **2**

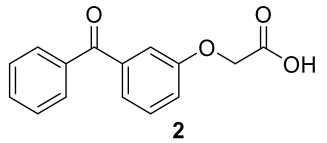

2

A mixture of **1** (6.26 g, 20.0 mmol) and trifluoroacetic acid (30.0 mL, 392 mmol) in chloroform (100 mL) was stirred overnight. The solvent and excess trifluoroacetic acid were removed to give a colored oil. The oil was diluted in acetonitrile (50 mL) and the solvent removed to give **2** as a colorless oil that solidified overtime (5.05 g, 98%); mp: 67 °C. IR (solid) ν/cm^{-1}: 1726 (C=O). λ_{max}(acetonitrile)/nm: 215 (log ε/dm^3 mol^{-1} cm^{-1}, 4.28), 252 (4.16), 301 (3.37). ^{1}H NMR (400 MHz, $CDCl_3$) δ: 7.81–7.78 (2H, m, $\underline{H}_{Ar}$), 7.62–7.57 (1H, m, $\underline{H}_{Ar}$), 7.52–7.46 (2H, m, $\underline{H}_{Ar}$), 7.44–7.40 (2H, m, $\underline{H}_{Ar}$), 7.38–7.37 (1H, m, $\underline{H}_{Ar}$), 7.21–7.16 (1H, m, $\underline{H}_{Ar}$), 4.75 (2H, s, $C\underline{H}_2$). ^{13}C NMR (101 MHz, $CDCl_3$) δ: 196.3 ($\underline{C}$=O_{BP}), 173.0 ($\underline{C}$OOH), 157.6 (O-$\underline{C}_{Ar}$), 139.2 ($\underline{C}_{Ar}$), 137.5 ($\underline{C}_{Ar}$), 132.8 ($\underline{C}H_{Ar}$), 130.2 ($\underline{C}H_{Ar}$), 129.7 ($\underline{C}H_{Ar}$), 128.5 ($\underline{C}H_{Ar}$), 124.3 ($\underline{C}H_{Ar}$), 119.6 ($\underline{C}H_{Ar}$), 115.4 ($\underline{C}H_{Ar}$), 65.0 ($\underline{C}H_2$). HRMS (APCI/QTOF) *m/z*: $[M+H]^+$ calcd for $C_{15}H_{13}O_4$ 257.0808 (100%), 258.0842 (17%); found 257.0814 (100%), 258.0847 (17%).

Methyl [2-(3-benzoylphenoxy)acetyl]-*L*-phenylalanyl-*L*-phenylalaninate – **3**

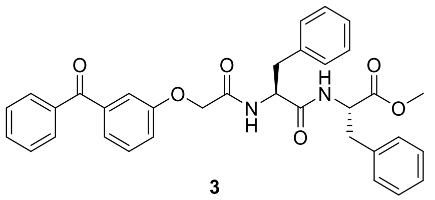

3

A solution of **2** (5.33 g, 20.8 mmol) in chloroform (100 mL) was cooled in an ice/brine bath. 4-Methylmorpholine (2.20 mL, 20.0 mmol) and isobutyl chloroformate (2.60 mL, 20.0 mmol) were added and the solution was stirred for 10 min. A separate solution of **TFA·FFOMe** (8.24 g, 18.7 mmol) and 4-methylmorpholine (2.20 mL, 20.0 mmol) in chloroform (100 mL) was added slowly and the resulting mixture was left to stir overnight and warm to room temperature. The solution was diluted with hydrochloric acid (1M aq., 100 mL) and the layers separated. The organic layer was washed with water (2 × 100 mL), dried over anhydrous sodium sulfate, filtered, and the solvent removed to give a colorless oil. The oil was vigorously stirred in diethyl ether (150 mL) overnight in which a white amorphous solid formed. The solid was collected at the filter, washed with diethyl ether (100 mL), and recrystallized from ethyl acetate to give **3** as a white solid (7.98 g, 76%); mp: 150 °C. IR (solid) ν/cm^{-1}: 1735 (C=O). λ_{max}(acetonitrile)/nm: 209sh (log ε/dm^3 mol^{-1} cm^{-1}, 4.56), 253 (4.15), 300 (3.31). ^{1}H NMR (400 MHz, DMSO-d_6) δ: 8.58 (1H, d, J 7.5, $N\underline{H}_{FOMe}$), 8.14 (1H, d, J 8.5, $N\underline{H}_{FFOMe}$), 7.74–7.65 (3H, m, $\underline{H}_{Ar}$), 7.56 (2H, t, J 7.5, $\underline{H}_{Ar}$), 7.44 (1H, t, J 7.9, H_{Ar}), 7.32–7.10 (13H, m, $\underline{H}_{Ar}$ and $\underline{H}_{ArF}$), 4.63 (1H, td, J 9.1, 4.4, $C\underline{H}_{FFOMe}$), 4.54–4.45 (3H, m, $C\underline{H}_{FOMe}$ and O-$C\underline{H}_2$), 3.58 (3H, s, $C\underline{H}_3$), 3.07–2.92 (3H, m, $C\underline{H}_{2FOMe}$ and 1/2$C\underline{H}_{2FFOMe}$), 2.83 (1H, dd, J 13.8, 9.5, 1/2$C\underline{H}_{2FFOMe}$). ^{13}C NMR (101 MHz, DMSO-d_6) δ: 195.3 ($\underline{C}$=O_{BP}), 171.7 ($\underline{C}$=O_{FOMe}), 170.9 ($\underline{C}$=O_{FFOMe}), 167.0 (CH_2-$\underline{C}$=O), 157.5 ($\underline{C}_{Ar}$-O), 138.3 ($\underline{C}_{Ar}$), 137.4 ($\underline{C}_{ArF}$), 137.0 ($\underline{C}_{ArF}$), 136.9 ($\underline{C}_{Ar}$), 132.7 ($\underline{C}H_{Ar}$), 129.7 ($\underline{C}H_{Ar}$), 129.6 ($\underline{C}H_{Ar}$), 129.2 ($\underline{C}H_{ArF}$), 129.0 ($\underline{C}H_{ArF}$), 128.5 ($\underline{C}H_{Ar}$), 128.2 ($\underline{C}H_{ArF}$), 128.0 ($\underline{C}H_{ArF}$), 126.5 ($\underline{C}H_{Ar}$), 126.2 ($\underline{C}H_{Ar}$), 122.6 ($\underline{C}H_{Ar}$), 118.7 ($\underline{C}H_{Ar}$), 115.6 ($\underline{C}H_{Ar}$), 66.7 (O-$\underline{C}H_2$), 53.6 ($\underline{C}H_{FOMe}$), 53.1 ($\underline{C}H_{FFOMe}$), 51.8 ($\underline{C}H_3$), 37.4 ($\underline{C}H_{2FFOMe}$), 36.6 ($\underline{C}H_{2FOMe}$). HRMS (APCI/QTOF) *m/z*: $[M+H]^+$ calcd for $C_{34}H_{33}N_2O_6$ 565.2333 (100%), 566.2366 (38%); found 565.2343 (100%), 566.2370 (36%).

[2-(3-Benzoylphenoxy)acetyl]-*L*-phenylalanyl-*L*-phenylalanine – **3BPAcFF**

To a stirred solution of **3** (9.87 g, 17.5 mmol) in tetrahydrofuran (100 mL) was slowly added a separate solution of lithium hydroxide (1.70 g, 71.0 mmol) in water (100 mL) that was left to stir overnight. The solution was slowly poured into vigorously stirred hydrochloric acid (1M aq., 400 mL) in which a white solid precipitated. The solid was collected at the filter, washed with water (2 × 100 mL), and was recrystallized from acetonitrile to give **3BPAcFF** as a white solid (8.59 g, 89%); mp: 175 °C; mp (DSC): 172 °C (onset), 182 °C (peak – first heating cycle, scan rate 10 °C min^{-1}). T_d: (5% weight loss) 259 °C. T_g: (DSC) 59 °C (second heating cycle, scan rate 10 °C min^{-1}). Anal. Calcd for $C_{33}H_{30}N_2O_6$: C, 72.0; H, 5.5; N, 5.1; Found: C, 72.4; H, 5.5; N, 5.1. IR (solid) ν/cm^{-1}: 1732 (C=O). λ_{max}(acetonitrile)/nm: 209sh (log ε/dm^3 mol^{-1} cm^{-1}, 4.74), 253 (4.16), 298 (3.34). ^{1}H NMR (400 MHz, DMSO-d$_6$) δ: 12.80 (1H, s, COO$\underline{H}$), 8.42 (1H, d, *J* 8.1, N$\underline{H}_{FOH}$), 8.12 (1H, d, *J* 8.6, N$\underline{H}_{FFOH}$), 7.74–7.65 (3H, m, $\underline{H}_{Ar}$), 7.56 (2H, t, *J* 7.8, $\underline{H}_{Ar}$), 7.44 (1H, t, *J* 7.9, $\underline{H}_{Ar}$), 7.32–7.08 (13H, m, C$\underline{H}_{Ar}$ and C$\underline{H}_{ArF}$), 4.63 (1H, td, *J* 9.0, 4.1, C$\underline{H}_{FFOH}$), 4.53–4.43 (3H, m, O-C$\underline{H}_2$ and C$\underline{H}_{FOH}$), 3.08 (1H, dd, *J* 13.9, 5.2, 1/2C$\underline{H}_{2FOH}$), 3.01 (1H, dd, *J* 13.8, 4.2, 1/2C$\underline{H}_{2FFOH}$), 2.93 (1H, dd, *J* 13.9, 8.7, 1/2C$\underline{H}_{2FOH}$), 2.83 (1H, dd, *J* 13.8, 9.6, 1/2C$\underline{H}_{2FFOH}$). ^{13}C NMR (101 MHz, DMSO-d$_6$) δ: 195.3 ($\underline{C}$=O$_{BP}$), 172.7 ($\underline{C}$OOH), 170.8 ($\underline{C}$=O$_{FFOH}$), 167.0 (CH$_2$-$\underline{C}$=O), 157.5 ($\underline{C}_{Ar}$-O), 138.3 ($\underline{C}_{Ar}$), 137.5 ($\underline{C}_{ArF}$), 137.4 ($\underline{C}_{ArF}$), 136.9 ($\underline{C}_{Ar}$), 132.7 ($\underline{C}$H$_{Ar}$), 129.7 ($\underline{C}$H$_{Ar}$), 129.6 ($\underline{C}$H$_{Ar}$), 129.2 ($\underline{C}$H$_{ArF}$), 129.1 ($\underline{C}$H$_{ArF}$), 128.6 ($\underline{C}$H$_{Ar}$), 128.2 ($\underline{C}$H$_{Ar}$), 127.9 ($\underline{C}$H$_{Ar}$), 126.4 ($\underline{C}$H$_{Ar}$), 126.2 ($\underline{C}$H$_{Ar}$), 122.6 ($\underline{C}$H$_{Ar}$), 118.7 ($\underline{C}$H$_{Ar}$), 115.6 ($\underline{C}$H$_{Ar}$), 66.7 (O-$\underline{C}$H$_2$), 53.5 ($\underline{C}$H$_{FOH}$), 53.2 ($\underline{C}$H$_{FFOH}$), 37.4 ($\underline{C}$H$_{2FFOH}$), 36.6 ($\underline{C}$H$_{2FOH}$). HRMS (APCI/QTOF) *m/z*: [M+H]$^+$ calcd for $C_{33}H_{31}N_2O_6$ 551.2177 (100%), 551.2209 (37%); found 551.2184 (100%), 552.2217 (38%).

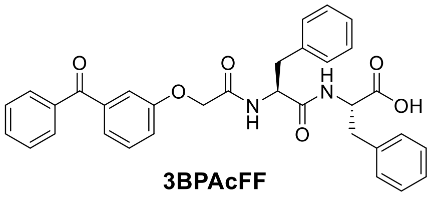

3BPAcFF

tert-Butyl 2-(4-benzoylphenoxy)acetate – **4**

A mixture of (4-hydroxyphenyl)(phenyl)methanone (5.30 g, 26.7 mmol), *tert*-butyl 2-bromoacetate (7.20 mL, 51.7 mmol), and potassium carbonate (10.0 g, 72.4 mmol) in acetone (100 mL) was refluxed overnight. The solution was allowed to cool to room temperature, filtered, and the solid washed with acetone (50 mL). The filtrate was collected, and the solvent removed to give a colored oil. The oil was diluted with chloroform (100 mL) and hydrochloric acid (1M aq., 100 mL). The layers were separated, and the organic layer washed with water (2 x 100 mL), dried over anhydrous sodium sulfate, filtered on a silica plug that was washed with dichloromethane (*c.a.* 100 mL), and the solvent removed to give a colorless oil that solidified overnight. The solid was sonicated in *n*-hexane (30 mL) and collected at the filter to give **2** as a white solid (7.35 g, 88%); mp: 96 °C. IR (solid) ν/cm^{-1}: 1752 (C=O). λ_{max}(acetonitrile)/nm: 216sh (log ε/dm^3 mol^{-1} cm^{-1}, 4.08), 252sh (4.00), 276sh (4.20), 282 (4.21), 289sh (4.16), 322sh (2.46). ^{1}H NMR (400 MHz, CDCl$_3$) δ: 7.83 (2H, 1/2AA'BB', $\underline{H}_{Ar}$), 7.77–7.74 (2H, m, $\underline{H}_{Ar}$), 7.60–7.54 (1H, m, $\underline{H}_{Ar}$), 7.49–7.45 (2H, m, $\underline{H}_{Ar}$), 6.95 (2H, 1/2AA'BB', $\underline{H}_{Ar}$), 4.60 (2H, s, C$\underline{H}_2$), 1.50 [9H, s, (C$\underline{H}_3$)$_3$]. ^{13}C NMR (101 MHz, CDCl$_3$) δ: 195.6 ($\underline{C}$=O$_{BP}$), 167.5 (CH$_2$-$\underline{C}$=O), 161.6 ($\underline{C}_{Ar}$-O), 138.3 ($\underline{C}_{Ar}$), 132.6 ($\underline{C}$H$_{Ar}$), 132.1 ($\underline{C}$H$_{Ar}$), 131.1 ($\underline{C}_{Ar}$), 129.9 ($\underline{C}$H$_{Ar}$), 128.3 ($\underline{C}$H$_{Ar}$), 114.3 ($\underline{C}$H$_{Ar}$), 82.9 [$\underline{C}$-(CH$_3$)$_3$], 65.7 ($\underline{C}$H$_2$), 28.2 [($\underline{C}$H$_3$)$_3$]. HRMS (APCI/QTOF) *m/z*: [M+H]$^+$ calcd for $C_{19}H_{21}O_4$ 313.1434 (100%), 314.1468 (21%); found 313.1441 (100%), 314.1470 (20%).

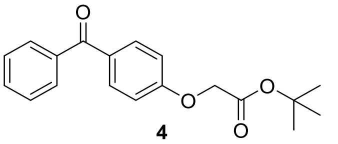

4

2-(4-Benzoylphenoxy)acetic acid – **5**

A mixture of **4** (7.35 g, 23.5 mmol) and trifluoroacetic acid (30.0 mL, 392 mmol) in chloroform (100 mL) was stirred overnight. The solvent and excess trifluoroacetic acid were removed to give a colored oil. The oil was sonicated in diethyl ether (100 mL) in which a white solid precipitated. The precipitate was collected at the filter and washed with diethyl ether (50 mL) to give **5** as a white solid (5.03 g, 84%); mp: 157 °C. IR (solid) ν/cm^{-1}: 1736 (C=O), 1711

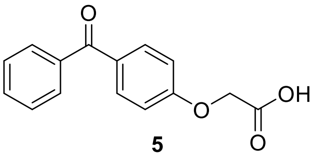

5

(C=O). λ_{max}(acetonitrile)/nm: 211sh (log ε/dm^3 mol^{-1} cm^{-1}, 4.14), 248sh (3.98), 273sh (4.18), 281 (4.19), 291sh (4.09), 330sh (2.44). ^{1}H NMR (400 MHz, DMSO-d$_6$) δ: 13.14 (1H, s, COO$\underline{H}$), 7.74 (2H, 1/2AA'BB', $\underline{H}_{Ar}$), 7.68–7.62 (3H, m, $\underline{H}_{Ar}$), 7.55 (2H, t, *J* 7.5, $\underline{H}_{Ar}$), 7.07 (2H, 1/2AA'BB', $\underline{H}_{Ar}$), 4.82 (2H, s, C$\underline{H}_2$). ^{13}C NMR (101 MHz, DMSO-d$_6$) δ: 193.4 ($\underline{C}$=O$_{BP}$), 169.7 ($\underline{C}$OOH), 161.4 (O-$\underline{C}_{Ar}$), 137.7 ($\underline{C}_{Ar}$), 132.1 ($\underline{C}$H$_{Ar}$), 132.0 ($\underline{C}$H$_{Ar}$), 129.8 ($\underline{C}_{Ar}$), 129.3 ($\underline{C}$H$_{Ar}$), 128.4 ($\underline{C}$H$_{Ar}$), 114.4 ($\underline{C}$H$_{Ar}$), 64.6 ($\underline{C}$H$_2$). HRMS (APCI/QTOF) *m/z*: [M−H]$^-$ calcd for $C_{15}H_{11}O_4$ 257.0808 (100%), 258.0842 (17%); found 257.0808 (100%), 258.0843 (19%).

Methyl [2-(4-benzoylphenoxy)acetyl]-*L*-phenylalanyl-*L*-phenylalaninate – **6**

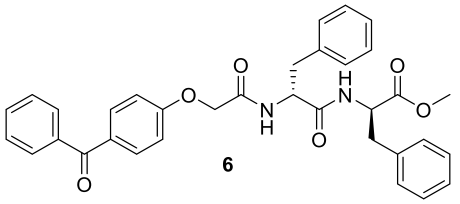

6

A solution of **5** (9.0 g, 35.1 mmol) in chloroform (100 mL) was cooled in an ice/brine bath. 4-Methylmorpholine (4.0 mL, 36.4 mmol) and isobutyl chloroformate (4.60 mL, 35.4 mmol) were added and the resulting mixture was stirred for 10 min. A separate solution of **TFA·FFOMe** (15.5 g, 35.1 mmol) and 4-methylmorpholine (4.0 mL, 36.4 mmol) in chloroform (100 mL) was added slowly and the resulting mixture was left to stir overnight and warm to room temperature. The solution was diluted with hydrochloric acid (1M aq., 100 mL) and the layers separated. The aqueous layer was extracted with chloroform (50 mL) and the combined organic extracts washed with water (2 × 100 mL), dried over anhydrous sodium sulfate, filtered, and the solvent removed to give a glassy solid. The solid was vigorously stirred in diethyl ether (300 mL) overnight to give a white amorphous solid. The solid was collected at the filter and washed with diethyl ether (100 mL) to give **6** as a white solid (16.4 g, 83%); mp: 121 °C. IR (solid) ν/cm^{-1}: 1736 (C=O). λ_{max}(acetonitrile)/nm: 213sh (log ε/dm^3 mol^{-1} cm^{-1}, 4.43), 250 (4.00), 273 (4.18), 281 (4.19), 291 (4.09), 322 (2.33). ^{1}H NMR (400 MHz, DMSO-d$_6$) δ: 8.61 (1H, d, *J* 7.7, N$\underline{H}_{FOMe}$), 8.25 (1H, d, J 8.8, N$\underline{H}_{FFOMe}$), 7.70–7.64 (5H, m, $\underline{H}_{Ar}$), 7.57 (2H, t, *J* 6.8, $\underline{H}_{Ar}$), 7.28–7.17 (9H, m, $\underline{H}_{ArF}$), 7.16–7.10 (1H, m, $\underline{H}_{ArF}$), 6.90 (2H, 1/2AA'BB', $\underline{H}_{Ar}$), 4.70–4.62 (1H, m, C$\underline{H}_{FFOMe}$), 4.58–4.49 (3H, m, C$\underline{H}_{FOMe}$ and O-C$\underline{H}_2$), 3.59 (3H, s, C$\underline{H}_3$), 3.09–2.92 (3H, m, C$\underline{H}_{2FOMe}$ and 1/2C$\underline{H}_{2FFOMe}$), 2.80 (1H, dd, J 13.9, 9.9, 1/2C$\underline{H}_{2FFOMe}$). ^{13}C NMR (101 MHz, DMSO-d$_6$) δ: 194.4 ($\underline{C}$=O$_{BP}$), 171.7 ($\underline{C}$=O$_{FOMe}$), 171.0 ($\underline{C}$=O$_{FFOMe}$), 166.7 (CH$_2$-$\underline{C}$=O), 161.4 ($\underline{C}_{Ar}$-O), 137.7 ($\underline{C}_{Ar}$), 137.5 ($\underline{C}_{ArF}$), 137.0 ($\underline{C}_{ArF}$), 132.1 ($\underline{C}$H$_{Ar}$), 132.0 ($\underline{C}$H$_{Ar}$), 129.8 ($\underline{C}_{Ar}$), 129.20 ($\underline{C}$H$_{Ar}$), 129.0 ($\underline{C}$H$_{Ar}$), 128.4 ($\underline{C}$H$_{Ar}$), 128.2 ($\underline{C}$H$_{ArF}$), 128.0 ($\underline{C}$H$_{ArF}$), 126.6 ($\underline{C}$H$_{ArF}$), 126.2 ($\underline{C}$H$_{ArF}$), 114.4 ($\underline{C}$H$_{Ar}$), 66.5 (O-$\underline{C}$H$_2$), 53.6 ($\underline{C}$H$_{FOMe}$), 53.1 ($\underline{C}$H$_{FFOMe}$), 51.8 ($\underline{C}$H$_3$), 37.5 ($\underline{C}$H$_{2FOMe}$), 36.6 ($\underline{C}$H$_{2FFOMe}$). HRMS (APCI/QTOF) *m/z*: [M+H]$^+$ calcd for $C_{34}H_{33}N_2O_6$ 565.2333 (100%), 566.2366 (38%); found 565.2349 (100%), 566.2377 (37%).

[2-(4-Benzoylphenoxy)acetyl]-*L*-phenylalanyl-*L*-phenylalanine – **4BPAcFF**

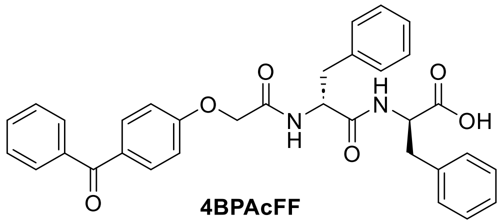

4BPAcFF

To a stirred solution of **6** (12.0 g, 21.2 mmol) in tetrahydrofuran (150 mL) was slowly added a separate solution of lithium hydroxide (2.10 g, 87.7 mmol) in water (150 mL) that was then left to stir overnight. The solution was slowly poured into vigorously stirred hydrochloric acid (1M aq., 500 mL), in which a white solid precipitated. The solid was collected at the filter and washed with water (2 × 200 mL). The solid was then sonicated in acetonitrile (200 mL), collected at the filter, and washed with acetonitrile (50 mL) to give **4BPAcFF** as a white solid (11.1 g, 95%); mp: 202 °C; mp (DSC): 190 °C (onset), 209 °C (peak – first heating cycle, scan rate 10 °C min^{-1}). T$_d$: (5% weight loss) 259 °C. T$_g$: (DSC) 69 °C (second heating cycle, scan rate 10 °C min^{-1}). Anal. Calcd for $C_{33}H_{30}N_2O_6$: C, 72.0; H, 5.5; N, 5.1; Found: C, 72.1; H, 5.5; N, 4.8. IR (solid) ν/cm^{-1}: 1724 (C=O). λ_{max}(acetonitrile)/nm: 250sh (log ε/dm^3 mol^{-1} cm^{-1}, 3.90), 273 (4.09), 281 (4.10), 291sh (4.00), 331 (2.19). ^{1}H NMR (400 MHz, DMSO-d$_6$) δ: 12.81 (1H, s, COO$\underline{H}$), 8.45 (1H, d, *J* 7.9, N$\underline{H}_{FOH}$), 8.24 (1H, d, *J* 8.7, N$\underline{H}_{FFOH}$), 7.69–7.63 (5H, m, $\underline{H}_{Ar}$), 7.56 (2H, t, *J* 7.5, $\underline{H}_{Ar}$), 7.28–7.16

(9H, m, $\underline{H}_{ArF}$), 7.14–7.09 (1H, m, $\underline{H}_{ArF}$), 6.89 (1H, 1/2AA'BB', $\underline{H}_{Ar}$), 4.66 (1H, td, *J* 10.0, 4.1, $C\underline{H}_{FFOH}$), 4.59–4.44 (3H, m, O-$C\underline{H}_2$ and $C\underline{H}_{FOH}$), 3.10 (1H, dd, *J* 13.9, 5.2, 1/2CH_{2FOH}), 3.04 (1H, dd, *J* 13.8, 4.1, 1/2CH_{2FFOH}), 2.94 (1H, dd, *J* 13.9, 8.8, 1/2CH_{2FOH}), 2.81 (1H, dd, *J* 13.8, 10.0, 1/2CH_{2FFOH}). ^{13}C NMR (101 MHz, DMSO-d_6) δ: 194.4 ($\underline{C}$=O_{BP}), 172.7 ($\underline{C}$=O_{FOH}), 170.9 ($\underline{C}$=O_{FFOH}), 166.7 (CH_2-$\underline{C}$=O), 161.4 ($\underline{C}_{Ar}$-O), 137.7 ($\underline{C}_{Ar}$), 137.6 ($\underline{C}_{ArF}$), 137.4 ($\underline{C}_{ArF}$), 132.1 ($\underline{C}H_{Ar}$), 132.0 ($\underline{C}H_{Ar}$), 129.8 ($\underline{C}_{Ar}$), 129.3, 129.2, 129.1, 128.4 ($\underline{C}H_{Ar}$), 128.2 ($\underline{C}H_{Ar}$), 128.0 ($\underline{C}H_{Ar}$), 126.4 ($\underline{C}H_{ArF}$), 126.2 ($\underline{C}H_{ArF}$), 114.4 ($\underline{C}H_{Ar}$), 66.5 (O-$\underline{C}H_2$), 53.5 ($\underline{C}H_{FFOH}$), 53.2 ($\underline{C}H_{FOH}$), 37.5 ($\underline{C}H_{2FFOH}$), 36.6 ($\underline{C}H_{2FOH}$). HRMS (ESI/QTOF) *m/z*: $[M\text{–}H]^-$ calcd for $C_{33}H_{29}N_2O_6$ 549.2031 (100%), 550.2064 (37%); found 549.2027 (100%), 550.2057 (36%).

Methyl (3-benzoylbenzoyl)-*L*-phenylalanyl-*L*-phenylalaninate – **7**

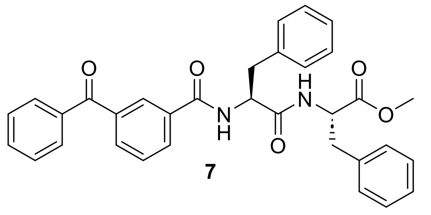

A solution of 3-benzoylbenzoic acid (5.35 g, 23.6 mmol) in chloroform (100 mL) was cooled in an ice/brine bath. 4-Methylmorpholine (2.60 mL, 23.6 mmol) and isobutyl chloroformate (3.07 mL, 23.6 mmol) were added and the solution was stirred for 10 min. A separate solution of **TFA·FFOMe** (10.3 g, 23.5 mmol) and 4-methylmorpholine (2.60 mL, 23.6 mmol) in chloroform (100 mL) was added slowly and the resulting mixture was left to stir overnight and warm to room temperature. The solution was diluted with hydrochloric acid (1M aq., 100 mL) and the layers separated. The organic layer was washed with water (2 × 100 mL), dried over anhydrous sodium sulfate, filtered, and the solvent removed to give a coloured solid. The solid was vigorously stirred in diethyl ether (200 mL) overnight, collected at the filter, and washed with diethyl ether (50 mL) to give the crude product. The crude solid was recrystallised from acetone, collected, and then recrystallised from methanol to give **7** as a white solid (7.17 g, 57 %); mp: 185 °C. IR (solid) ν/cm^{-1}: 1740 (C=O). λ_{max}(acetonitrile)/nm: 208sh (log ε/dm^3 mol^{-1} cm^{-1}, 4.60), 249 (4.28), 272 (3.66). ^{1}H NMR (400 MHz, DMSO-d_6) δ: 8.78 (1H, d, *J* 8.5, $N\underline{H}_{FOMe}$), 8.55 (1H, d, *J* 7.5, $N\underline{H}_{FFOMe}$), 8.10 (1H, t, *J* 1.8, $\underline{H}_{Ar}$), 8.04 (1H, dt, *J* 7.7, 1.4, $\underline{H}_{Ar}$), 7.86 (1H, dt, *J* 7.7, 1.2, $\underline{H}_{Ar}$), 7.77–7.69 (3H, m, $\underline{H}_{Ar}$), 7.66–7.56 (3H, m, $\underline{H}_{Ar}$), 7.33–7.29 (2H, m, $\underline{H}_{ArF}$), 7.34–7.12 (8H, m, $\underline{H}_{ArF}$), 4.76 (1H, ddd, *J* 11.0, 8.5, 4.0, $C\underline{H}_{FOMe}$), 4.51 (1H, td, *J* 8.4, 6.0, $C\underline{H}_{FFOMe}$), 3.59 (3H, s, $C\underline{H}_3$), 3.10–2.88 (4H, m, $C\underline{H}_{2FFOMe}$). ^{13}C NMR (101 MHz, DMSO-d_6) δ: 195.4 ($\underline{C}$=O_{BP}), 171.7 ($\underline{C}$=O_{FOMe}), 171.4 ($\underline{C}$=O), 165.4 ($\underline{C}$=O_{FFOMe}), 138.2 ($\underline{C}_{ArF}$), 137.05 ($\underline{C}_{Ar}$ or $\underline{C}_{ArF}$), 137.01 ($\underline{C}_{Ar}$ or $\underline{C}_{ArF}$), 136.7 ($\underline{C}_{ArF}$), 134.3 ($\underline{C}_{Ar}$), 132.9 ($\underline{C}H_{Ar}$), 132.1 ($\underline{C}H_{Ar}$), 131.3 ($\underline{C}H_{Ar}$), 129.7 ($\underline{C}H_{Ar}$), 129.12 ($\underline{C}H_{Ar}$ or $\underline{C}H_{ArF}$), 129.06 ($\underline{C}H_{Ar}$ or $\underline{C}H_{ArF}$), 128.7 ($\underline{C}H_{Ar}$ or $\underline{C}H_{ArF}$), 128.6 ($\underline{C}H_{Ar}$ or $\underline{C}H_{ArF}$), 128.4 ($\underline{C}H_{Ar}$), 128.2 ($\underline{C}H_{ArF}$), 128.0 ($\underline{C}H_{ArF}$), 126.5 ($\underline{C}H_{ArF}$), 126.2 ($\underline{C}H_{ArF}$), 54.5 ($\underline{C}H_{FOMe}$), 53.7 ($\underline{C}H_{FFOMe}$), 51.8 ($\underline{C}H_3$), 36.9 ($\underline{C}H_{2FOMe}$), 36.5 ($\underline{C}H_{2FFOMe}$). HRMS (APCI/QTOF) *m/z*: $[M+H]^+$ calcd for $C_{33}H_{31}N_2O_5$ 535.2227 (100%), 536.2260 (37%); found 535.2232 (100%), 536.2262 (36%).

(3-Benzoylbenzoyl)-*L*-phenylalanyl-*L*-phenylalanine – **3BPFF**

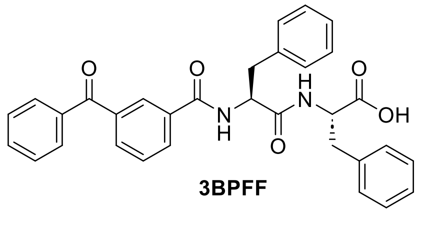

To a stirred solution of **7** (7.17 g, 13.4 mmol) in tetrahydrofuran (150 mL) was slowly added a separate solution of lithium hydroxide (1.40 g, 58.4 mmol) in water (150 mL) that was left to stir overnight. The solution was slowly poured into vigorously stirred hydrochloric acid (1M aq., 500 mL) in which a white solid precipitated. The solid was collected at the filter and washed with water (100 mL) and acetonitrile (200 mL). The solid was then recrystallized from acetonitrile to give **3BPFF** as a white solid (6.98 g, *ca.* 100%) that had identical characterization data to that previously reported[4]; mp: 217 °C; mp (DSC): 216 °C (onset – first heating cycle, scan rate 10 °C min^{-1}). T_d: (5% weight loss) 266 °C. T_g: (DSC) 75 °C (second heating cycle, scan rate 100 °C min^{-1}). IR (solid) ν/cm^{-1}: 1724 (C=O). λ_{max}(acetonitrile)/nm: 208sh (log ε/dm^3 mol^{-1} cm^{-1}, 4.44), 249 (4.12), 272 (3.53). Anal. Calcd for $C_{32}H_{28}N_2O_6$: C, 73.8; H, 5.4; N, 5.4; Found: C, 73.6; H, 5.5; N, 5.2. ^{1}H NMR (400 MHz,

DMSO-d_6) δ: 12.77 (1H, s, COO$\underline{H}$), 8.78 (1H, d, J 8.6, N$\underline{H}_{FFOH}$), 8.35 (1H, d, J 7.8, N$\underline{H}_{FOH}$), 8.10 (1H, t, J 1.8, $\underline{H}_{Ar}$), 8.04 (1H, dt, J 7.8, 1.4, $\underline{H}_{Ar}$), 7.86 (1H, dt, J 7.7, 1.4, $\underline{H}_{Ar}$), 7.77–7.69 (3H, m, $\underline{H}_{Ar}$), 7.66–7.56 (3H, m, $\underline{H}_{Ar}$), 7.31 (1H, d, J 6.7, $\underline{H}_{ArF}$), 7.26–7.11 (8H, m, $\underline{H}_{ArF}$), 4.75 (1H, ddd, J 11.8, 7.9, 3.4, C$\underline{H}_{FFOH}$), 4.48 (1H, td, J 8.2, 5.2, C$\underline{H}_{FOH}$), 3.13–3.05 (2H, m, 1/2C$\underline{H}_{2FFOH}$), 3.00–2.88 (2H, m, 1/2C$\underline{H}_{2FFOH}$). ^{13}C NMR (101 MHz, DMSO-d_6) δ: 196.4 ($\underline{C}$=O_{BP}), 172.7 ($\underline{C}$OOH), 171.3 ($\underline{C}$=O), 165.4 ($\underline{C}$=O_{FFOH}), 138.3 ($\underline{C}_{ArF}$), 137.4 ($\underline{C}_{ArF}$), 137.1 ($\underline{C}_{Ar}$), 136.7 ($\underline{C}_{Ar}$), 134.3 ($\underline{C}_{Ar}$), 132.9 ($\underline{C}H_{Ar}$), 132.2 ($\underline{C}H_{Ar}$), 131.3 ($\underline{C}H_{Ar}$), 129.7 (2C, $\underline{C}H_{Ar}$ and $\underline{C}H_{ArF}$), 129.1 ($\underline{C}H_{Ar}$), 128.7 ($\underline{C}H_{Ar}$ or $\underline{C}H_{ArF}$), 128.6 ($\underline{C}H_{Ar}$ or $\underline{C}H_{ArF}$), 128.4 ($\underline{C}H_{Ar}$), 128.1 ($\underline{C}H_{ArF}$), 128.0 ($\underline{C}H_{ArF}$), 126.4 ($\underline{C}H_{ArF}$), 126.2 ($\underline{C}H_{ArF}$), 54.6 ($\underline{C}H_{FFOH}$), 53.5 ($\underline{C}H_{FOH}$), 36.9 ($\underline{C}H_{2FFOH}$), 36.6 ($\underline{C}H_{2FOH}$). HRMS (ESI/QTOF) m/z: $[M-H]^-$ calcd for $C_{32}H_{27}N_2O_5$ 519.1925 (100%), 520.1958 (36%); found 519.1928 (100%), 520.1958 (35%).

Additional Data

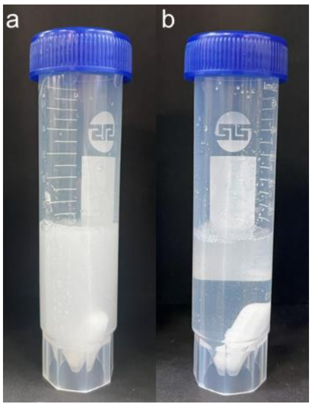

Figure S1. Photographs of **4BPAcFF** (10 mg mL^{-1}) (a) after being stirred overnight with one molar equivalent of 0.1 M sodium hydroxide and (b) after being adjusted to pH 10.0±0.1.

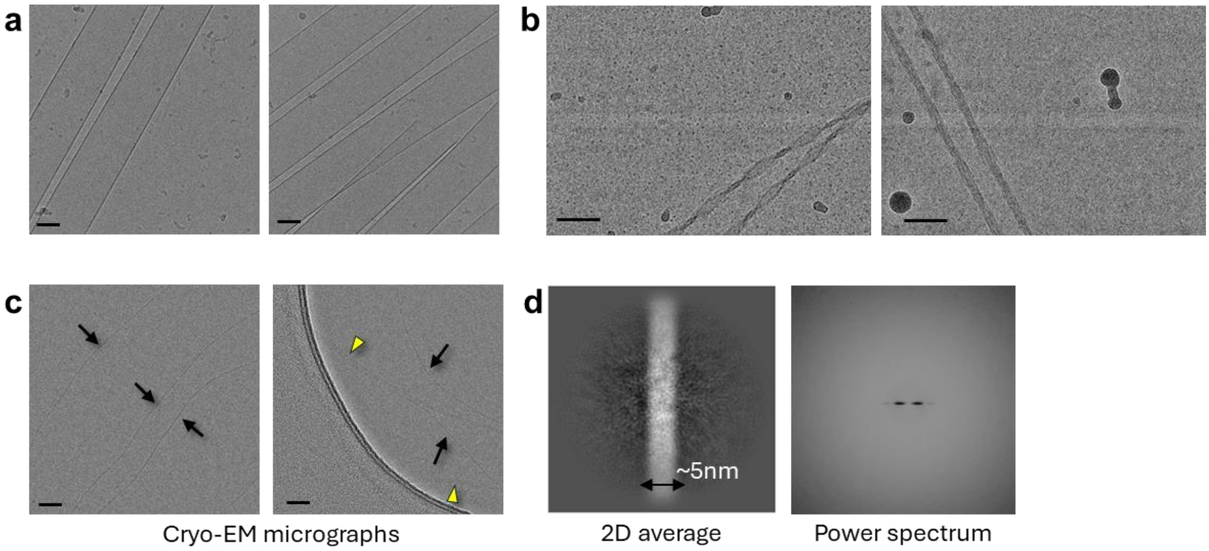

Figure S2. Representative cryo-EM micrographs of (a) **3BPFF**, (b) **3BPAcFF** and (c) **4BPAcFF**. (d) 2D average and power spectrum of **4BPAcFF** showing unordered packing of peptide in the fiber. Black arrows and yellow arrowheads indicate the individual **4BPAcFF** fibers and their bundles, respectively. Scale bar ≈50 nm.

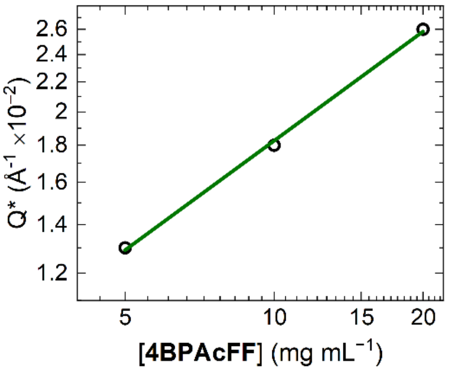

Figure S3. Graph of Q* (from Figure 2) against concentration with fit. The data show a scaling of ~ $c^{1/2}$.

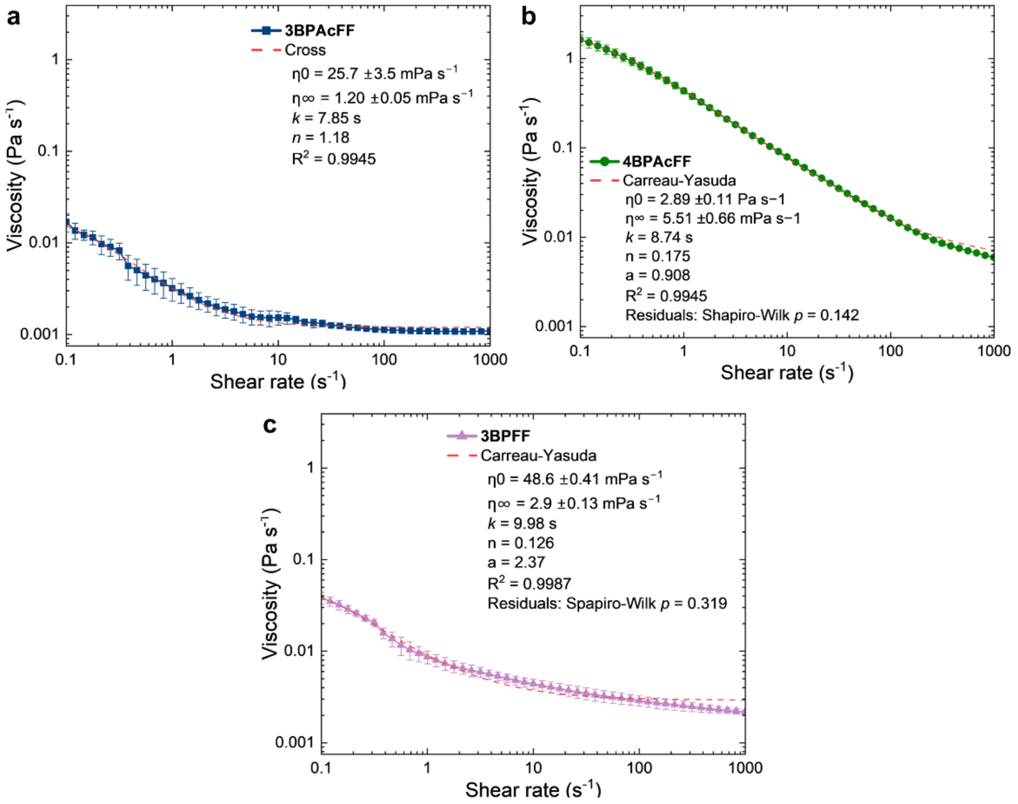

Figure S4. Viscosity fits for (a) **3BPAcFF**, (b) **4BPAcFF**, and (c) **3BPFF** with fitting parameters.

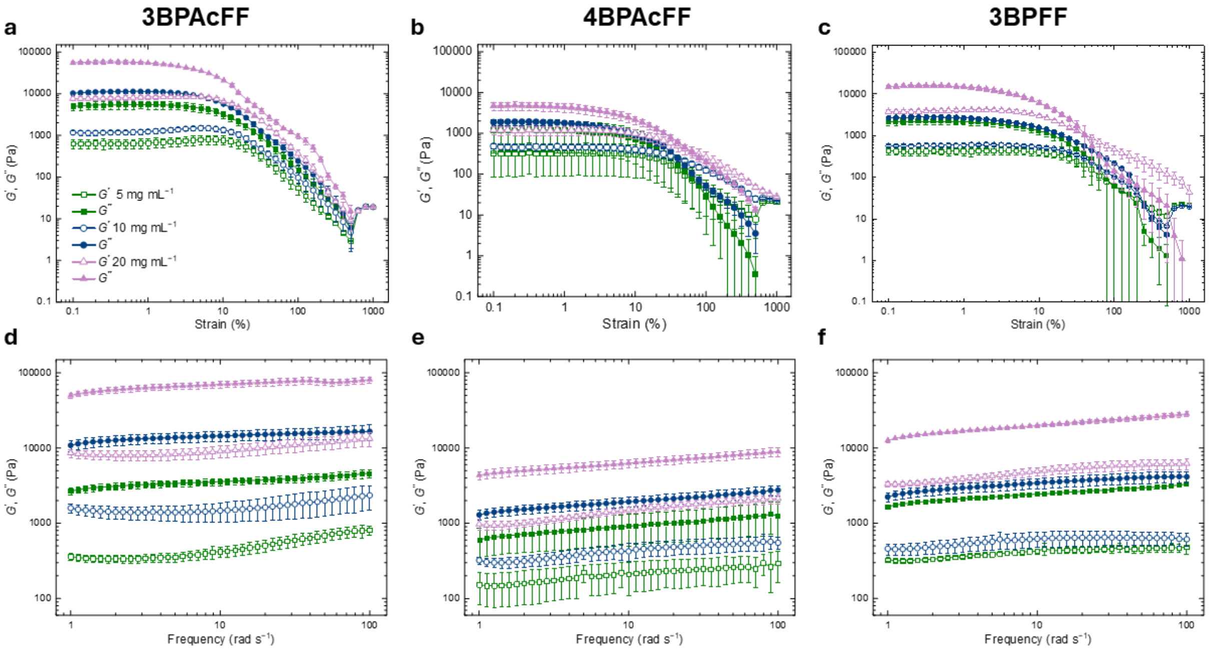

Figure S5. Typical strain (top) and frequency sweeps (bottom) for gels formed by addition of a calcium salt for **3BPAcFF** (a, d), **4BPAcFF** (b, e), and **3BPFF** (c, f), respectively. Measurements were performed in triplicates and plotted data are presented as mean ± s.d.

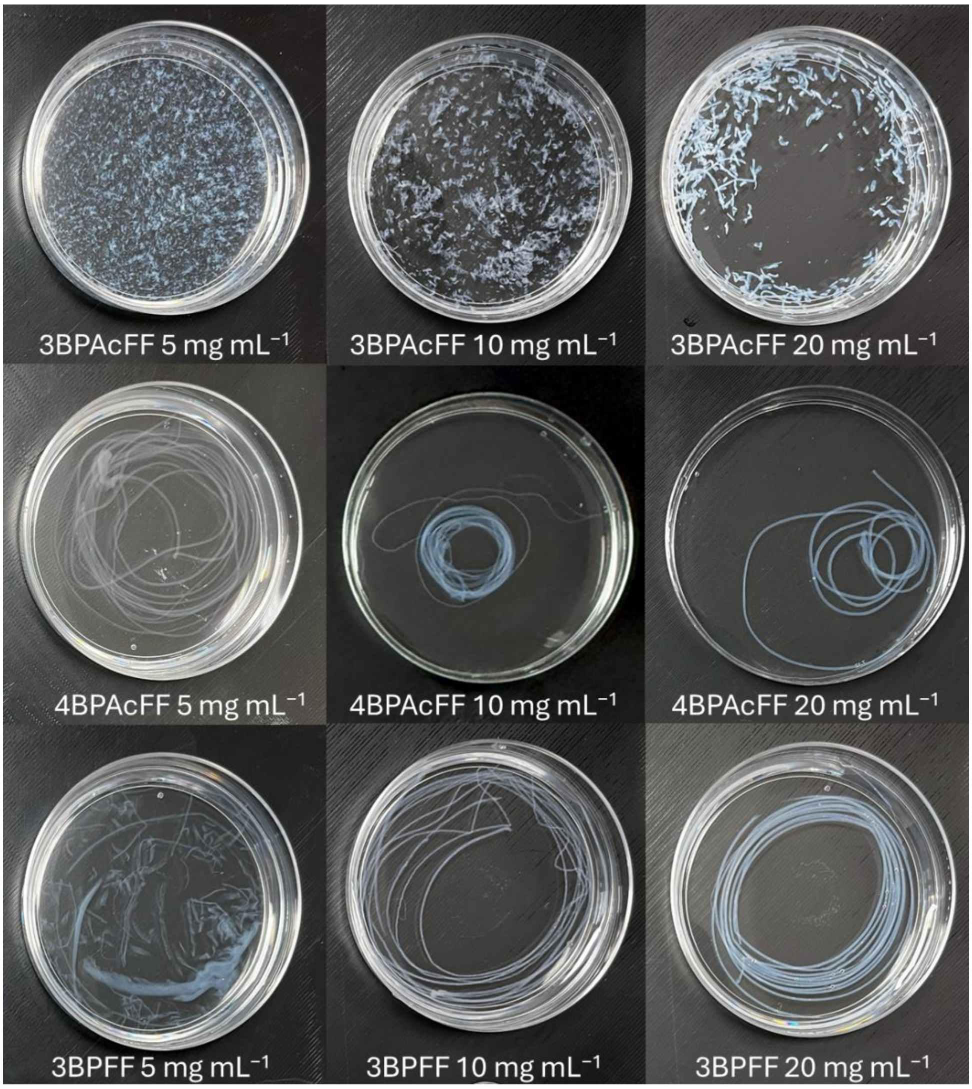

Figure S6. Photographs of attempts at forming gel noodles. The Petri dish is 90 mm in diameter. The concentration referred to is that of the precursor solution used to try and form the gel noodles.

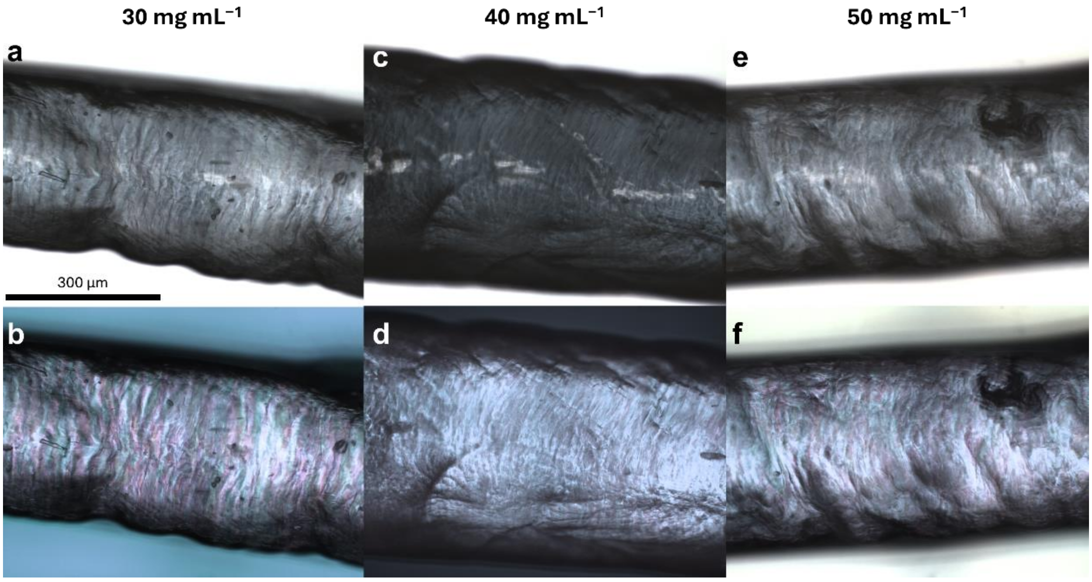

Figure S7. A section of **4BPAcFF** gel noodles made from 30, 40, and 50 mg mL^{-1} gelator solutions viewed under the microscope (a,c,d) and when using cross-polarized light (b,d,f), respectively.

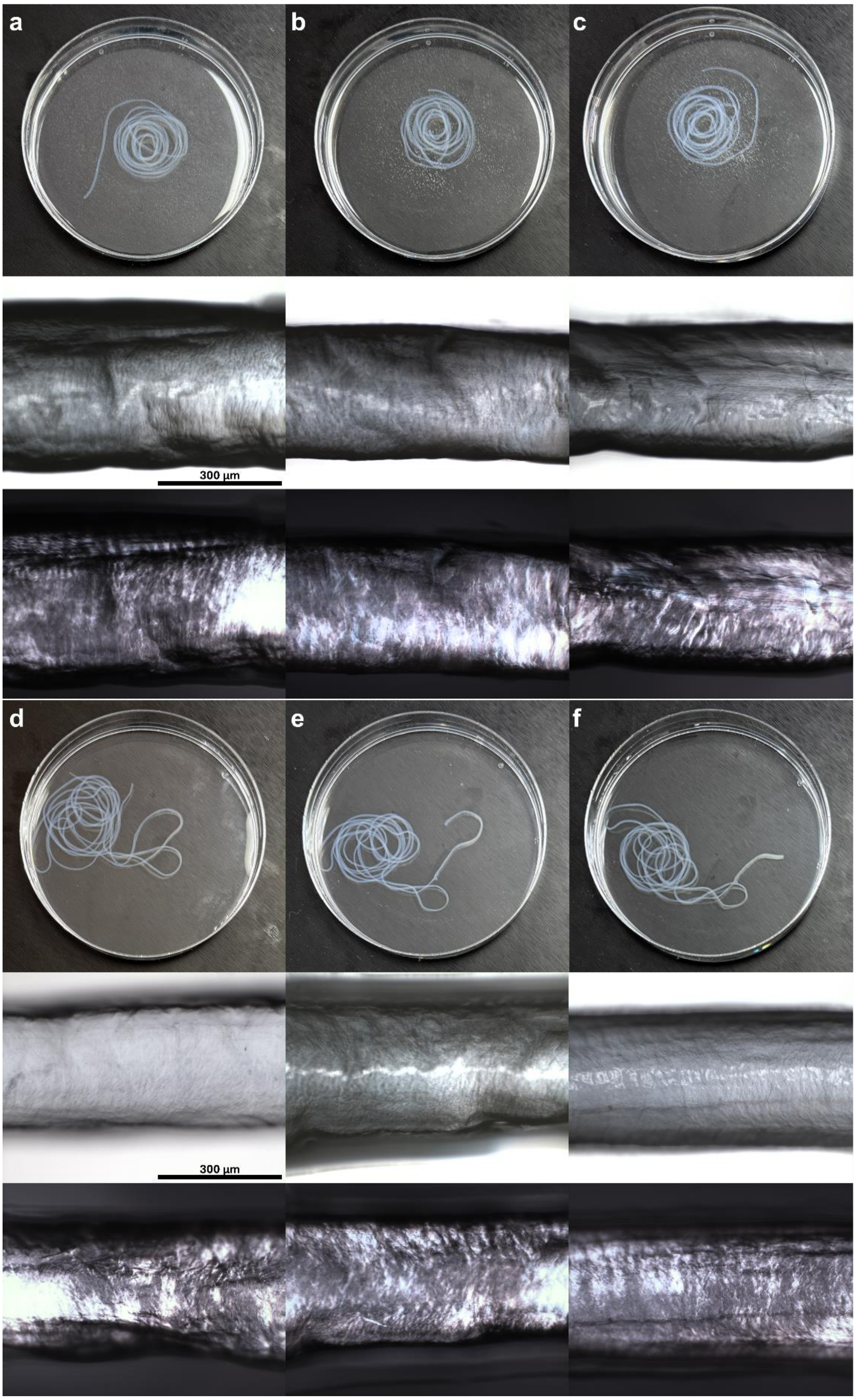

Figure S8. 4BPAcFF gel noodles in trigger medium (top), viewed under the microscope (middle), and with polarized light (bottom) after 3 (a), 5 (b), and 7 (c) days. The white solids in (b) and (c) are salt precipitating. **4BPAcFF** gel noodles that have been solvent exchanged to water (top), viewed under the microscope (middle), and with polarized light (bottom) after 3 (d), 5 (e), and 7 (f) days. The Petri dishes are 90 mm in diameter.

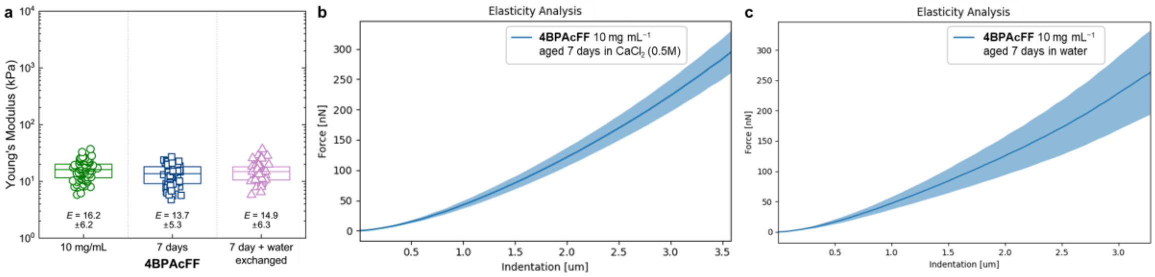

Figure S9. (a) Comparison of nanoindentation data taken from an as-prepared **4BPAcFF** gel noodle (10 mg mL^{-1}) and after aging for 7 days in either the trigger medium ($CaCl_2$, 0.5M) or water with the average and standard deviation given adjacent. (b, c) Corresponding force-indentation (F-δ) curves for samples aged in the trigger medium and water, respectively.

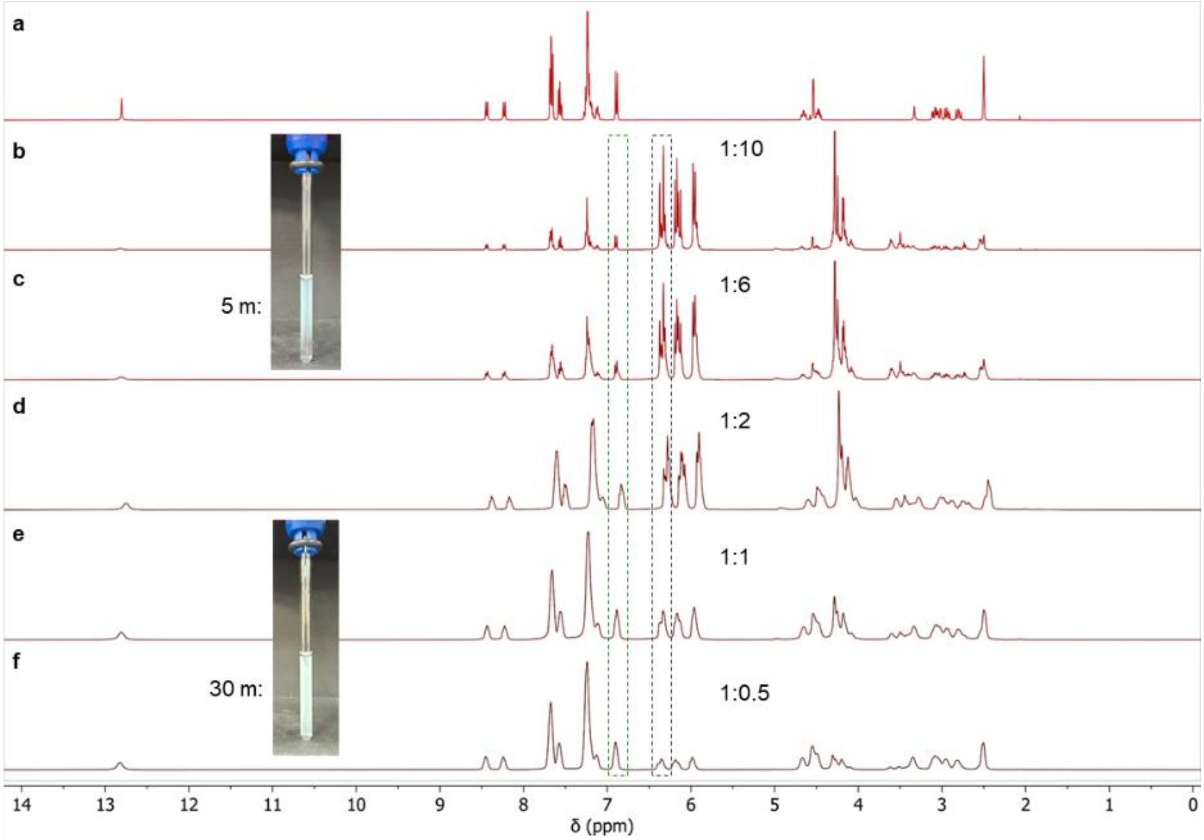

Figure S10. Partial ^{1}H NMR (400 MHz, 0.7 mL DMSO-d_6) of (a) 15 mg of **4PBAcFF**, and 1:4 mixture of **4BPAcFF** and PETA after (b) 0, (c) 5, (d) 10, (e) 20, and (f) 30 minutes of 365 nm light irradiation. The boxed regions represent the selected peak ratio of **4BPAcFF** to PETA with values obtained by integration shown next to the boxes. Inset: photographs of the NMR tube showing the progression of polymerization after 5 minutes (top) to 30 minutes (bottom).

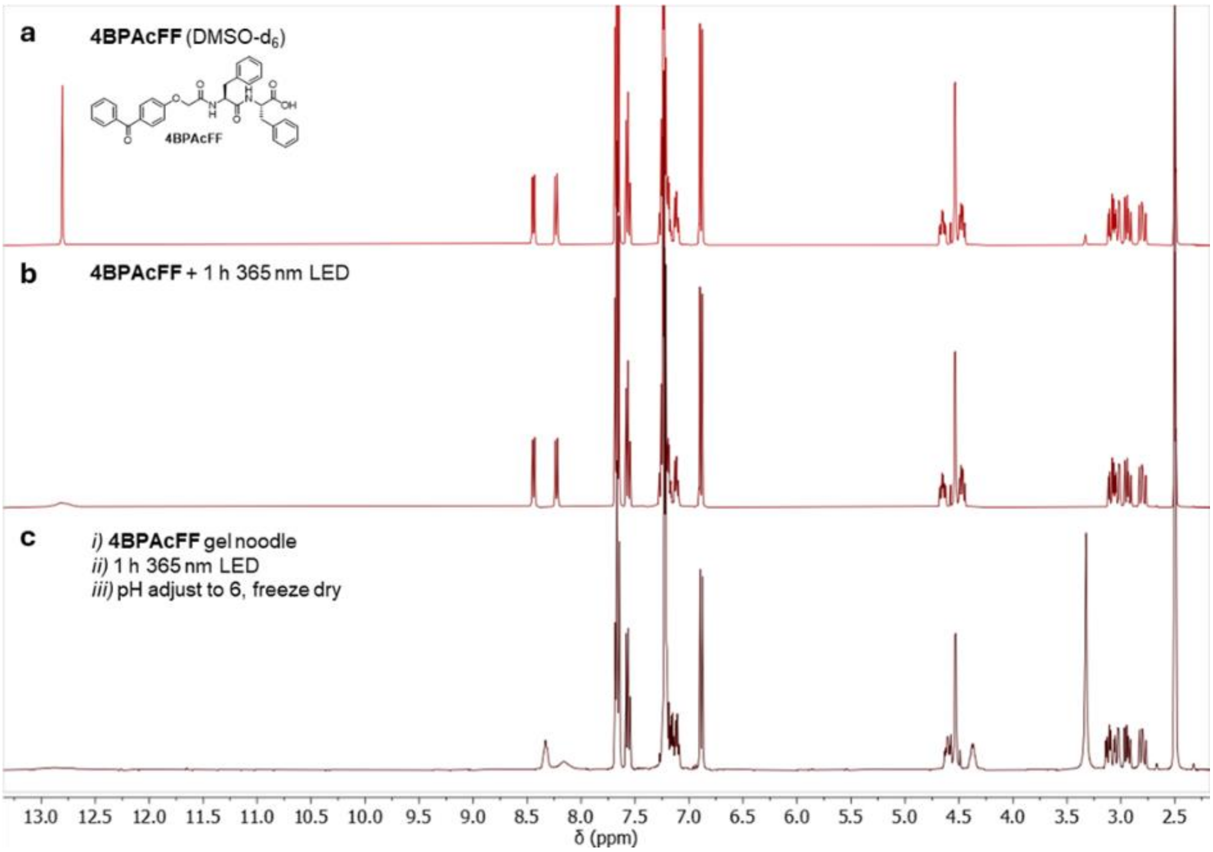

Figure S11. Partial ^{1}H NMR (400 MHz, 0.7 mL DMSO-d_6) of (a) 15 mg of **4PBAcFF**, (b) after 1 h of 365 nm LED irradiation, and (c) after a piece of gel noodle (triggered with $CaCl_2$) was removed from the trigger solution, irradiated with a 365 nm LED for 1 h, then dissolved in water, the pH adjusted to ≈6, and freeze-dried.

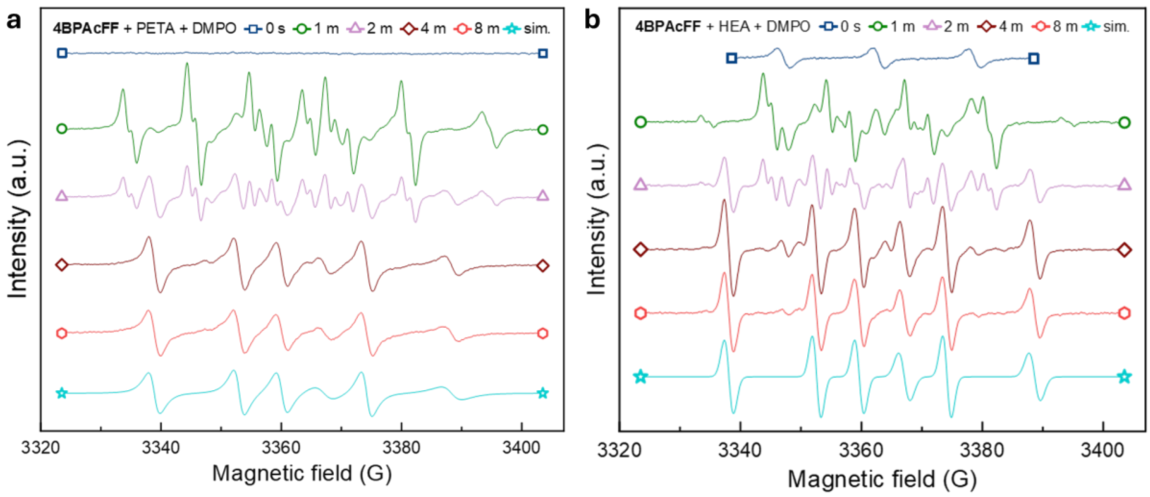

Figure S12. Electron paramagnetic resonance spectra of **4BPAcFF** (1 wt%), DMPO (1 wt%), and (a) PETA or (b) HEA with 365 nm LED irradiation; sim. = simulation. The spectra at four and eight minutes are dominated by the trapped polymer chain radicals (see main text). The spectra at one minute involve contributions from alkyl peroxyl (ROO$^{\bullet}$) radicals (a_N = 12.8 G, a_H = 10.2 G) formed by a reaction of carbon-centered radicals with dissolved oxygen. As the dissolved oxygen is consumed, these adducts are gradually replaced by the carbon-centered polymer radical adducts observed at two, four, and eight minutes. The triplet in the (b), HEA spectrum at zero minutes is due to a nitroxide contamination in HEA monomer; this triplet disappears under irradiation after one minute.

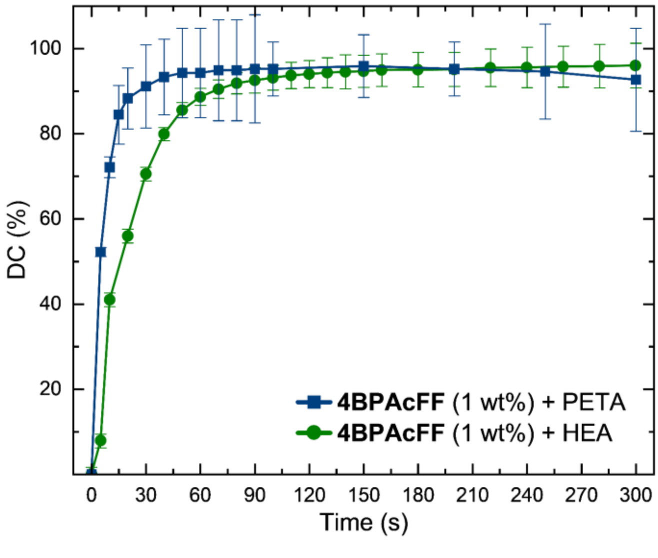

Figure S13. Reduction of the double bond peak area [double bond conversion (DC)] in FT-IR with irradiation given with peak fitting error.

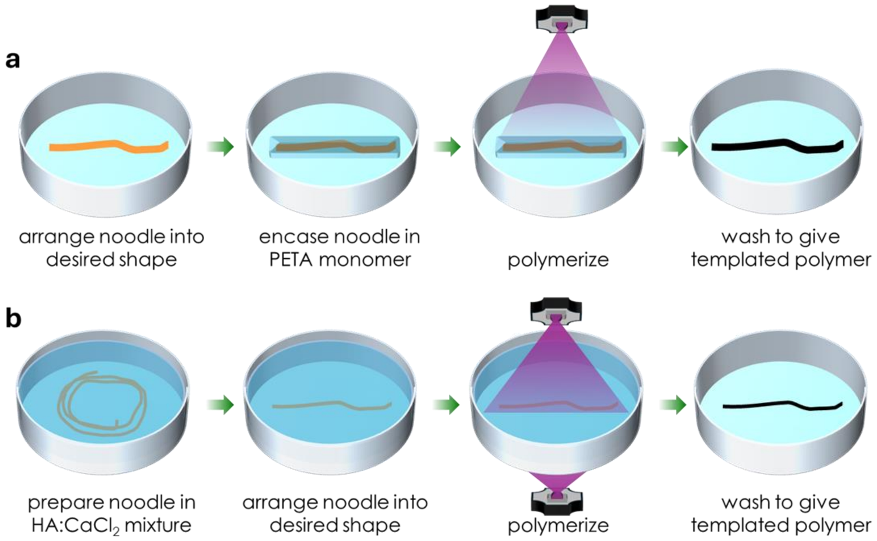

Figure S14. Schematic of how the gel noodle can be polymerized using the two techniques. (a) The gel noodle is encased in PETA monomer before polymerization, or (b) the gel noodle is prepared in a monomer:trigger mixture solution before polymerization.

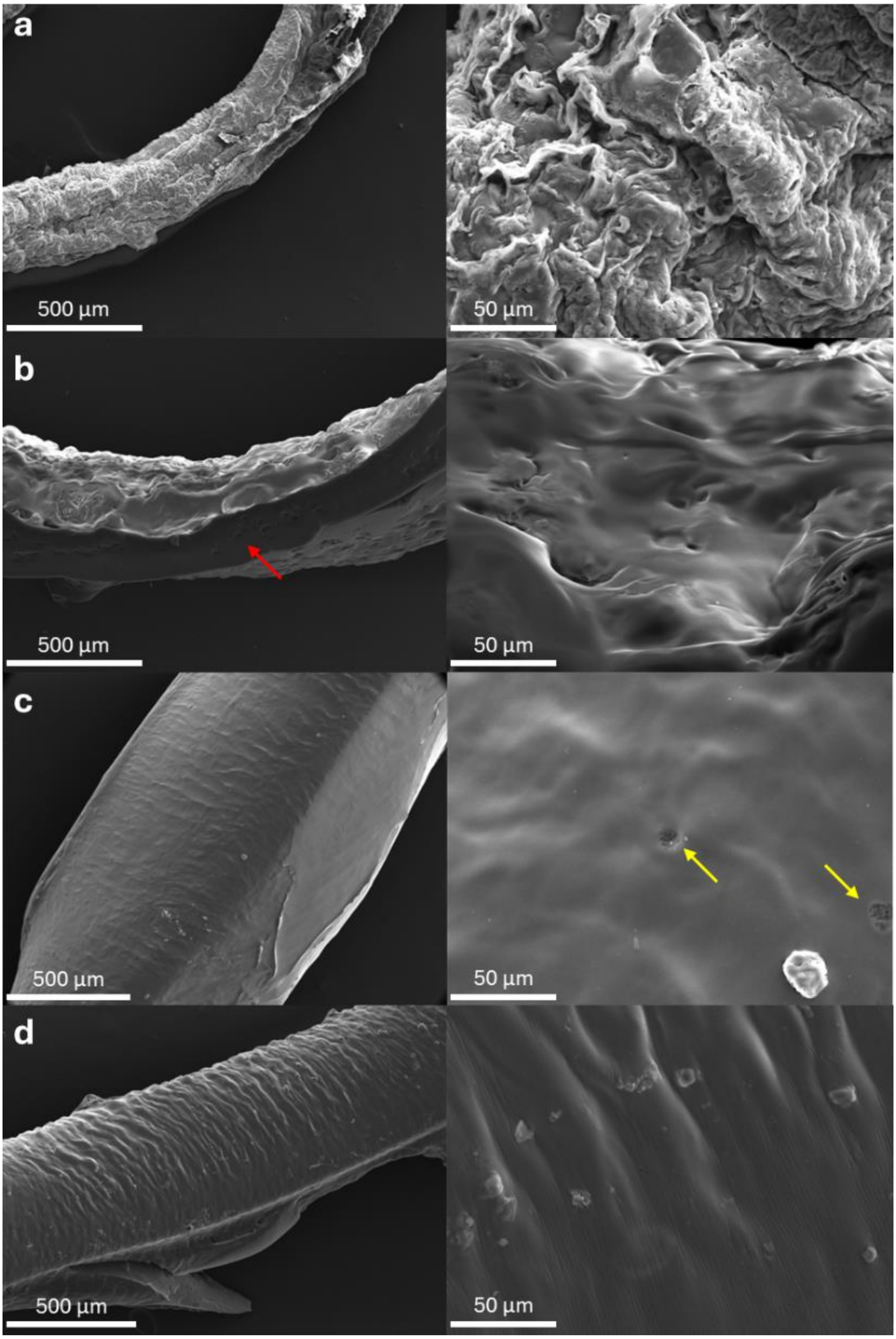

Figure S15. SEM images of polymer structures made form **4BPAcFF** gel noodles and PETA after (a) 2 hours, and (b) 4 hours, and HEA after (c) 1 hour and (d) 2 hours. Note: the flat section (red arrow) is the contact point with the Petri dish; the two spots (yellow arrows) are artifacts from beam damage.

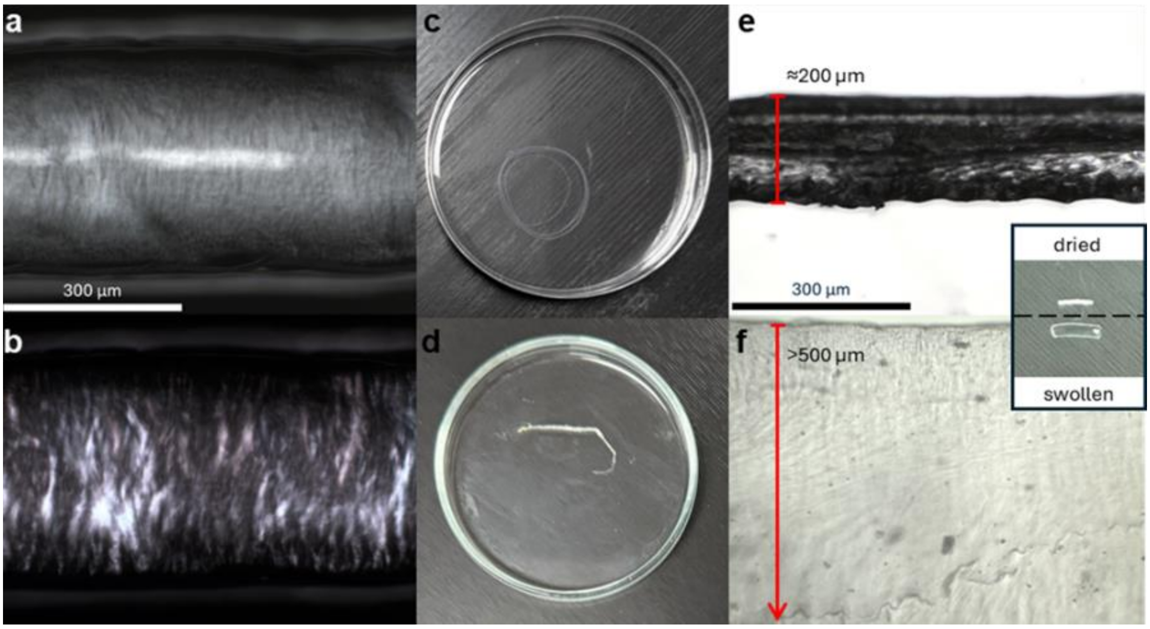

Figure S16. 4BPAcFF gel noodles prepared in a 1:4 (by volume) 2-hydroxyethyl acrylate:calcium chloride (0.5 M) trigger solution. (a) Gel noodles viewed under the microscope, and (b) with polarized light. Photographs of the gel noodle (c) as prepared, and (d) after irradiation with 2 × 365 nm LEDs for 1 h and solvent washing to give the polymer structure. (e) Solvent washed and dried HEA polymer structure viewed under the microscope (≈200 µm), and (f) after soaking in water for 1 h showing significant swelling to over 500 µm. Inset: comparison photograph of a piece of (e) and (f).

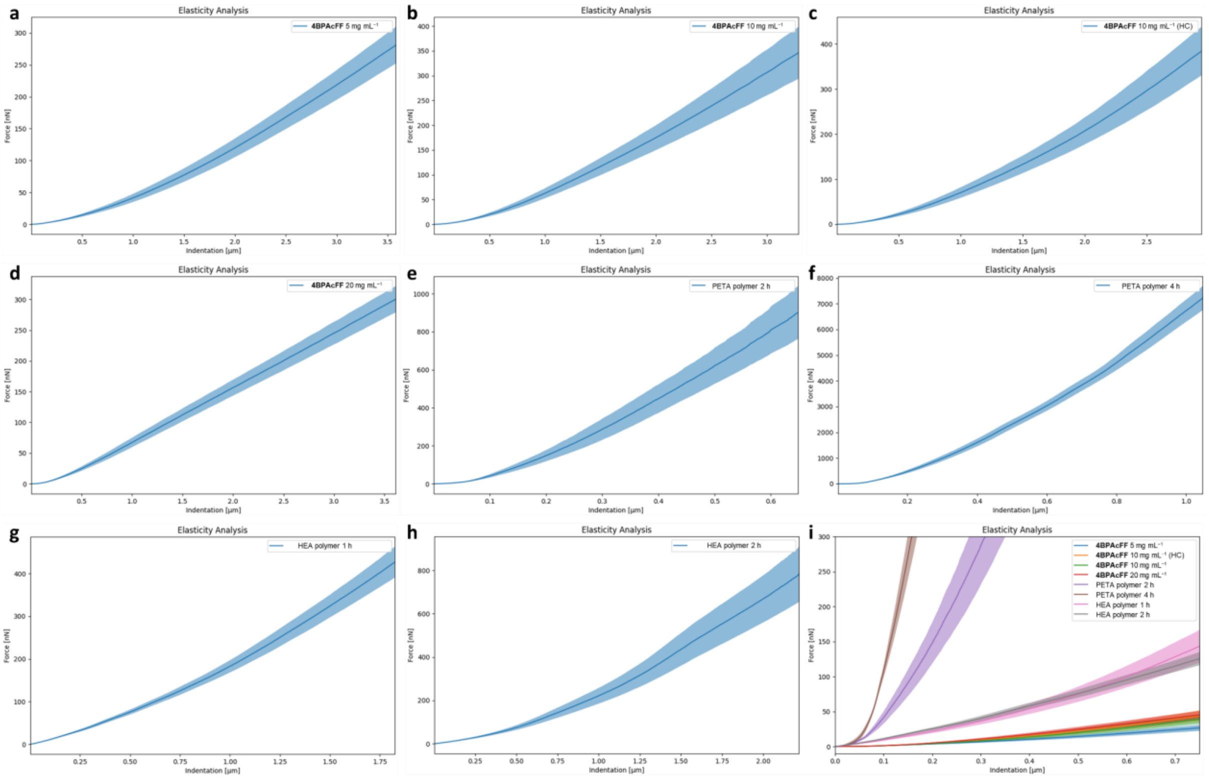

Figure S17. Force-indentation (F-δ) curves extracted from nanoindentation data of **4BPAcFF** gel noodles at (a) 5 mg mL^{-1}, (b) 10 mg mL^{-1}, (c) 10 mg mL^{-1} (heat-cool), (d) 20 mg mL^{-1}, after polymerization with PETA for (e) 2 h, (f) 4 h, or HEA for (g) 1 h, (h) 2 h, and (i) combined for comparison. The curves represent the average force-indentation response and were only analyzed up to a maximum of 0.3 µm.

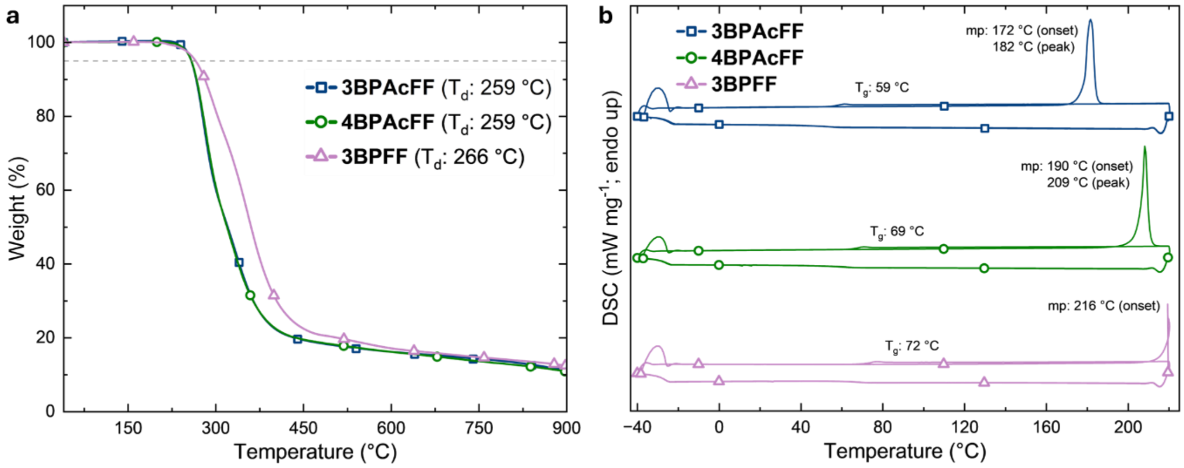

Figure S18. Thermal properties of the gelators. (a) Thermogravimetric analysis (TGA). The dashed horizontal line represents a 5% mass loss (T_d). Measurements were conducted under a nitrogen atmosphere with a heating rate of 10 °C min^{-1} and are smoothed (5 pt. Savitzky-Golay). (b) Differential scanning calorimetry (DSC) analysis. The measurements were carried out in the temperature range from −40–220 °C at 10 °C min^{-1}.

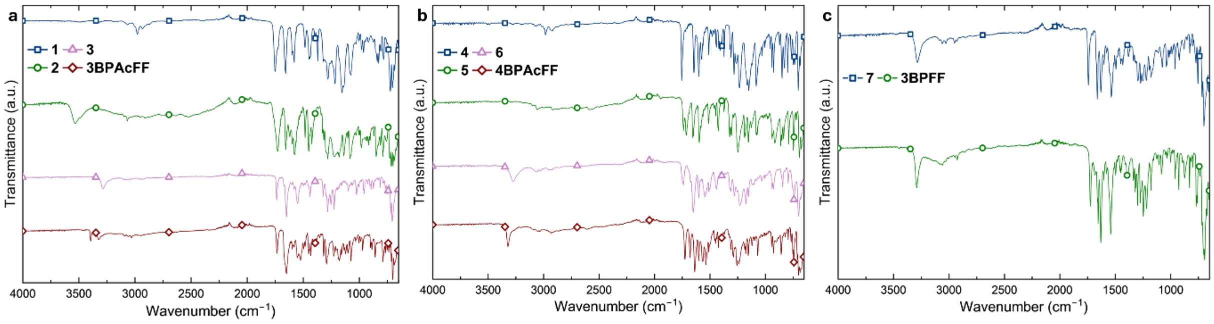

Figure S19. FT-IR spectra of each of the intermediates compared to their corresponding final gelator: (a) **3BPAcFF**; (b) **4BPAcff**; and (c) **3BPFF**.

Table S1. Fitting parameters for SAXS data in Figure 1d. The data were fitted to a cylinder or hollow cylinder model (A) combined with a power law model (B). Values that were manually fixed during the fitting process have been labelled with *.

Cylinder + PL	3BPAcFFOH 10 mg/mL pregel		Hollow Cylinder + PL	3BPFF 10 mg/mL pregel	
	Value	Error		Value	Error
Scale	1		Scale	1	
Background (cm^{-1})	0.035*		Background (cm^{-1})	0.006*	
A_scale	2.19 e-05	1.07 e-06	A_scale	0.0009	4.74 e-06
A_radius (Å)	68.43	2.52	A_radius (Å)	367.9	0.13
A_length (Å)	1000*	/	A_thickness (Å)	42.31	0.25
B_scale	3.22 e-08	2.15 e-9	A_length (Å)	10541	105.02
B_power	3.56	0.011	B_scale	1.08e-08	6.25e-10
χ^2	0.96		B_power	3.75	0.01
			χ^2	3.4	

Table S2. Fitting parameters for SAXS data in Figures 1d and 1e. The data were fitted to a cylinder model (A) combined with a power law model (B). Values that were manually fixed during the fitting process have been labelled with *. Note here that the high χ^2 values are due the presence of the Q* peak in the data, which is not captured by the fits.

Cylinder + PL	4BPAcFF 5 mg/mL pregel		4BPAcFF 10 mg/mL pregel		4BPAcFF 20 mg/mL pregel	
	Value	Error	Value	Error	Value	Error
Scale	1		1		1	
Background (cm^{-1})	0.007*		0.007*		0.009*	
A_scale	0.0005	5.94 e-06	0.0016	8.25 e-06	0.0044	1.29e-05
A_radius (Å)	29.875	0.2	26.83	0.09	23.302	0.05
A_length (Å)	1251	149.36	212.01	1.82	101.85	0.37
B_scale	8.58 e-09	7.52 e-10	2.78 e-09	1.72e-10	3.46e-10	2.06e-11
B_power	3.8	0.01	4.06	0.01	4.46	0.01
χ^2	0.74		14.3		360.89	

Table S3. Two-sample t-tests results for the Young's modulus of **4BPAcFF** gel noodles. Statistical analyses were conducted using MATLAB (version R2024b). Two-sample t-tests assuming unequal variances (Welch's t-test) were performed to compare the mechanical property distributions between different gel noodles. The **4BPAcFF** 10 mg mL^{-1} gel noodle was used as the reference sample, and p-values were calculated for each gel noodle against this reference. Significance levels were defined as follows: ns (not significant) for $p > 0.05$, * for $p \leq 0.05$, ** for $p \leq 0.01$, *** for $p \leq 0.001$, and **** for $p \leq 0.0001$.

Sample	Significance
**4BPAcFF** 5 mg mL^{-1}	**
**4BPAcFF** 10 mg mL^{-1} (heat-cooled)	ns
**4BPAcFF** 20 mg mL^{-1}	****
**4BPAcFF** 10 mg mL^{-1} $CaCl_2$ aged 7 days	**
**4BPAcFF** 10 mg mL^{-1} water aged 7 days	ns

NMR Spectra

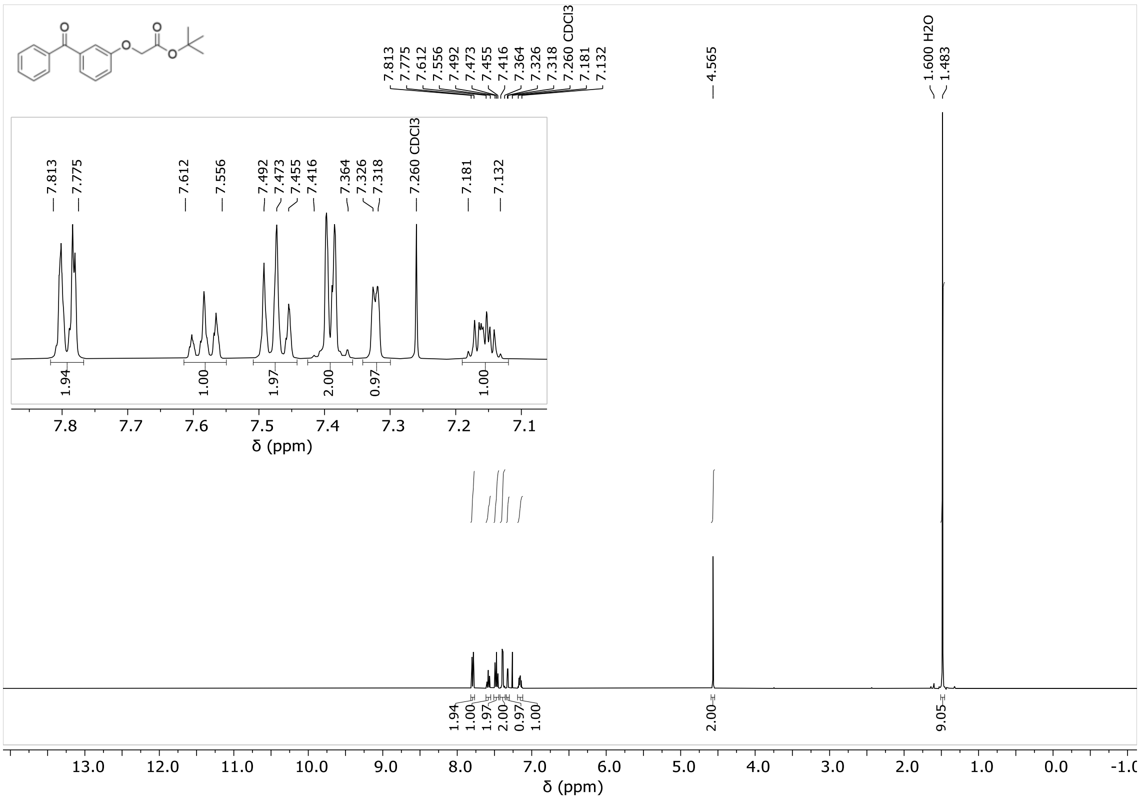

Figure S20. ^{1}H NMR (400 MHz, $CDCl_3$) of **1** with an expansion (insert) showing the region from 7.1–7.9.

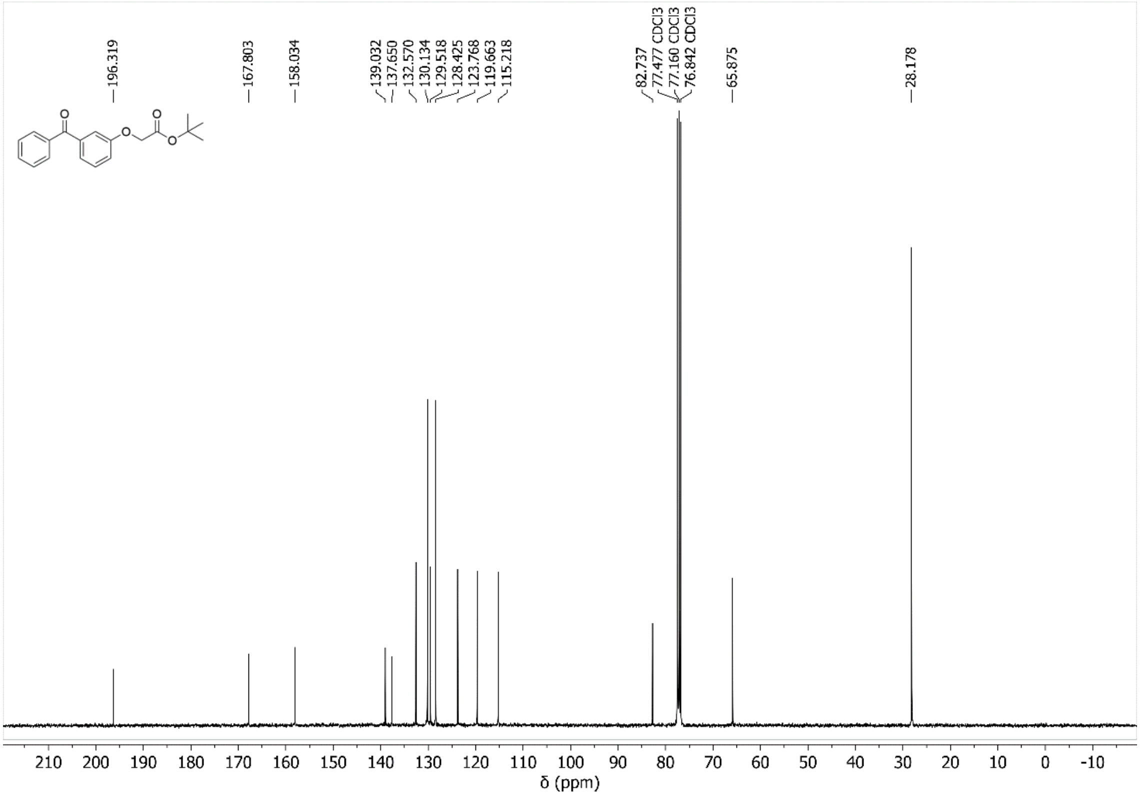

Figure S21. ^{13}C NMR (101 MHz, $CDCl_3$) of **1**.

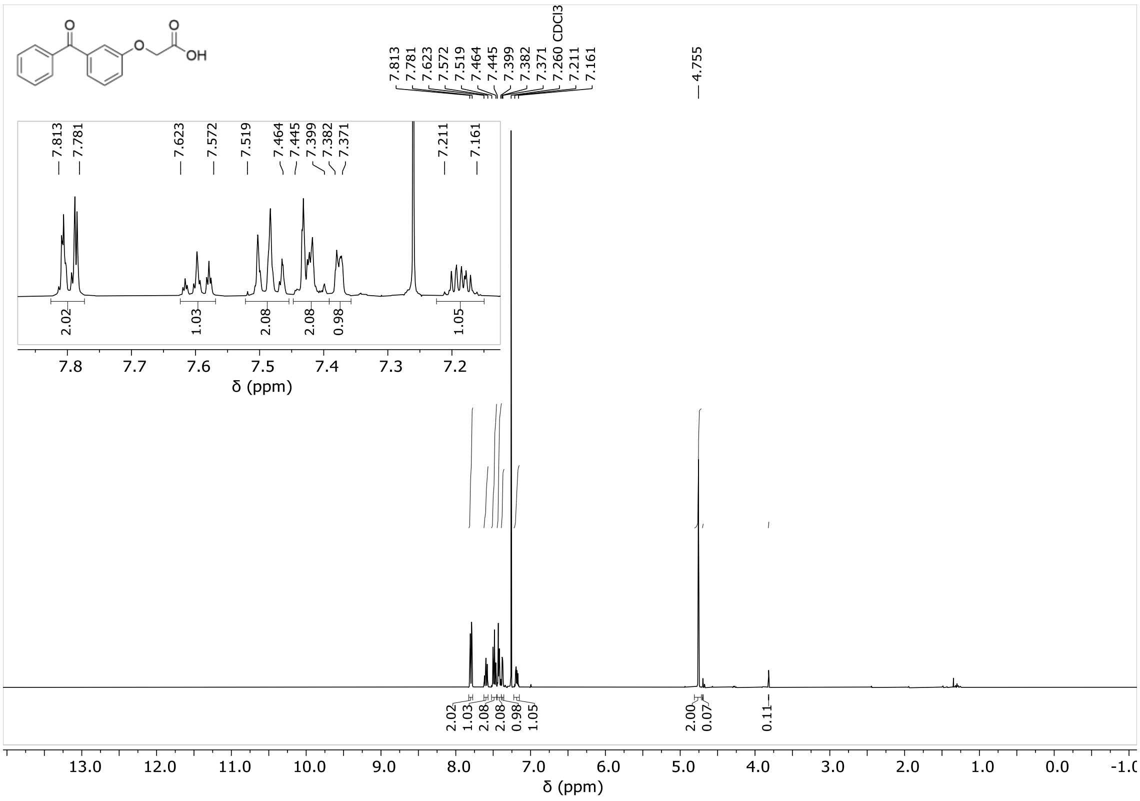

Figure S22. ^{1}H NMR (400 MHz, $CDCl_3$) of **2** with an expansion (insert) showing the region from 7.1–7.9.

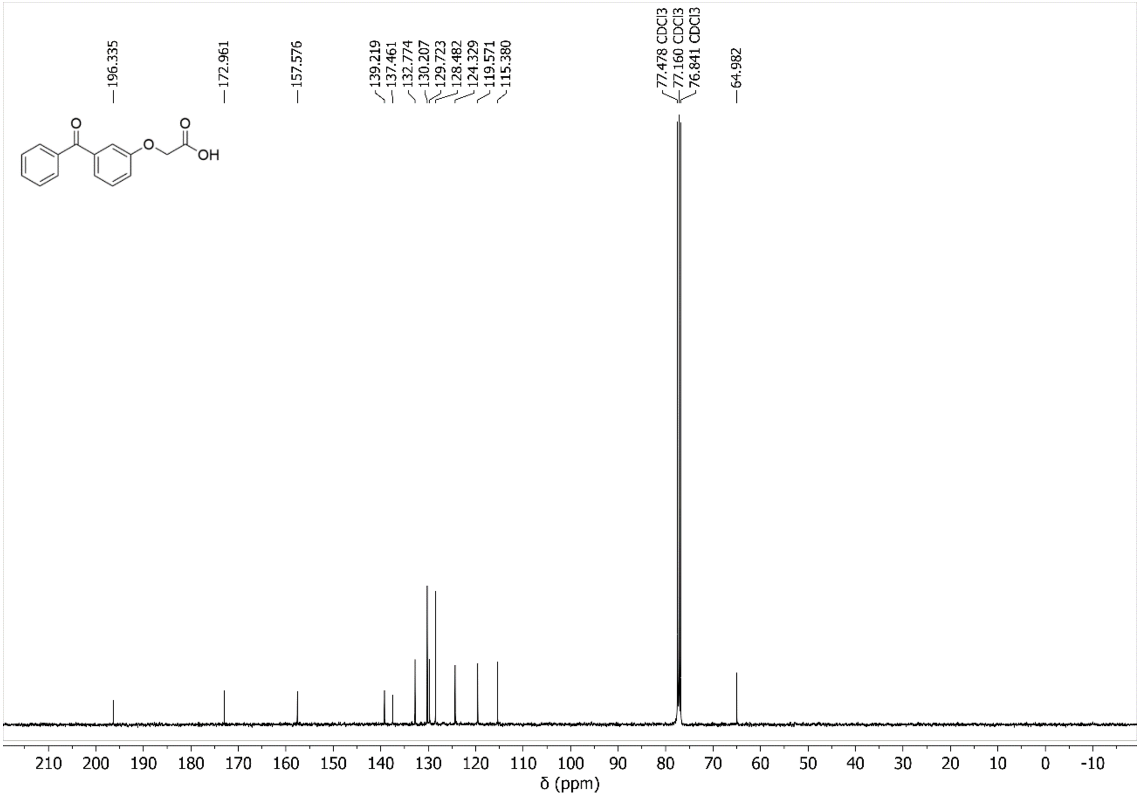

Figure S23. ^{13}C NMR (101 MHz, $CDCl_3$) of **2**.

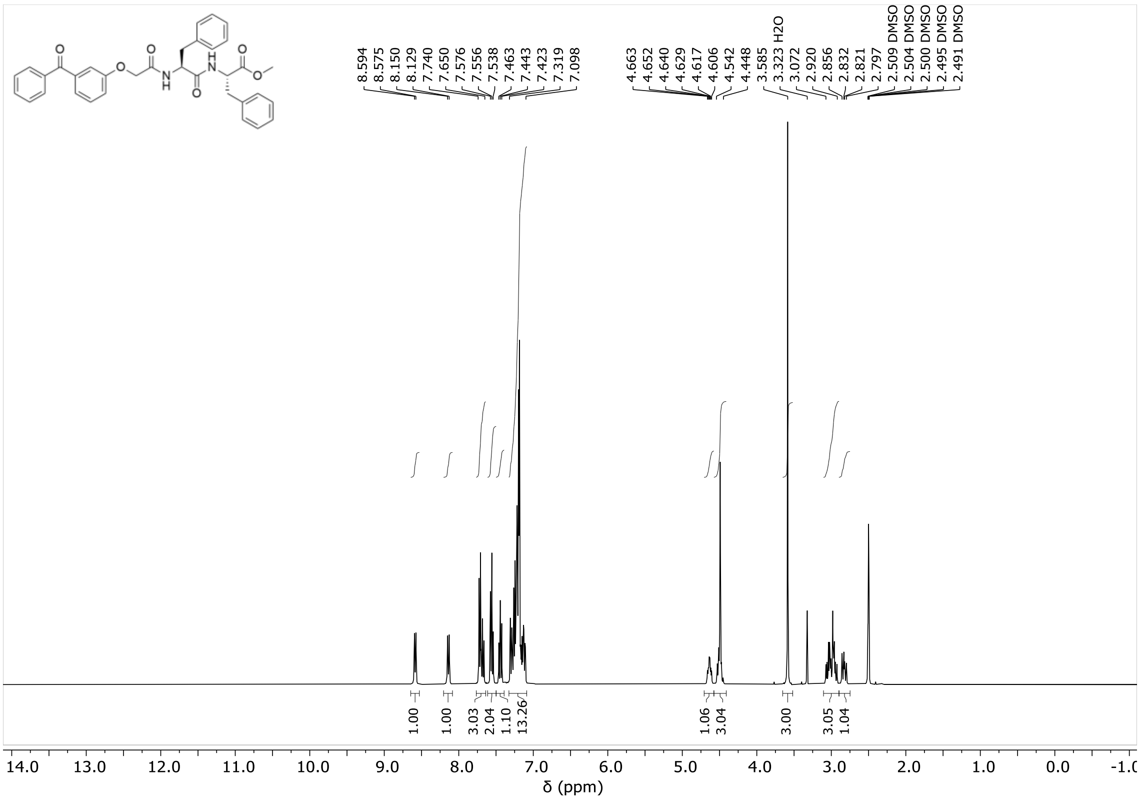

Figure S24. ^{1}H NMR (400 MHz, $CDCl_3$) of **3**.

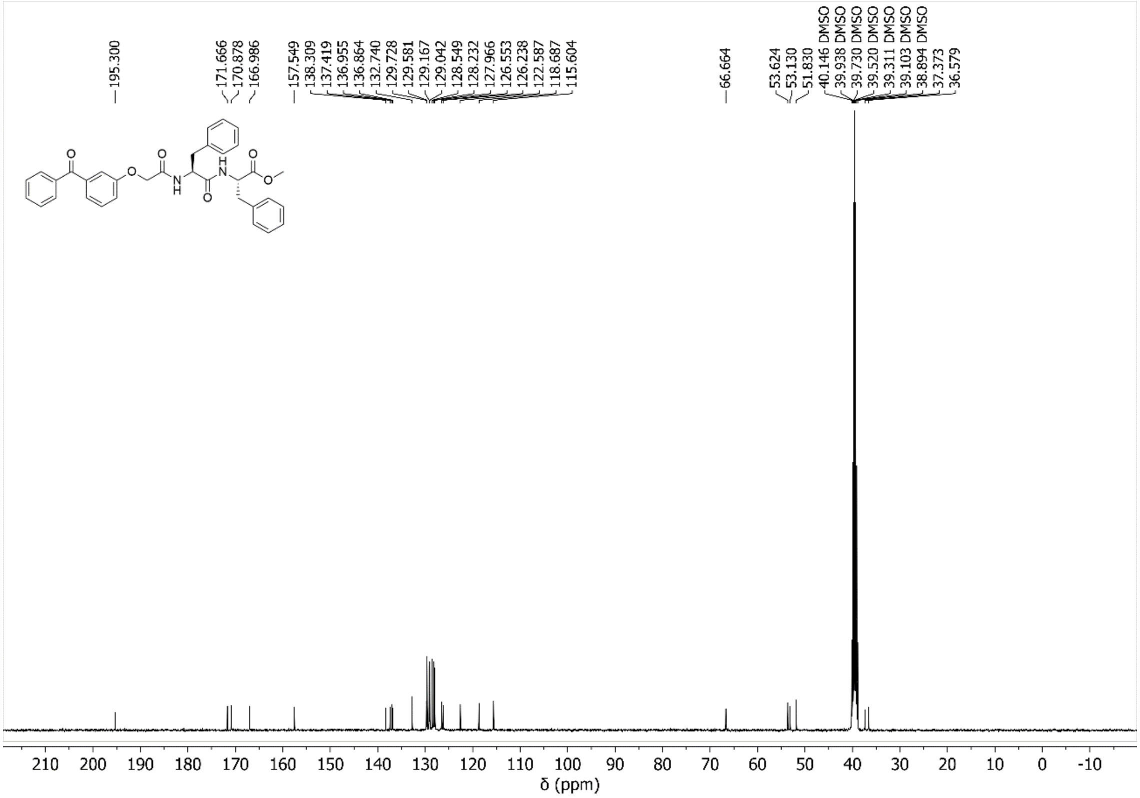

Figure S25. ^{13}C NMR (101 MHz, $CDCl_3$) of **3**.

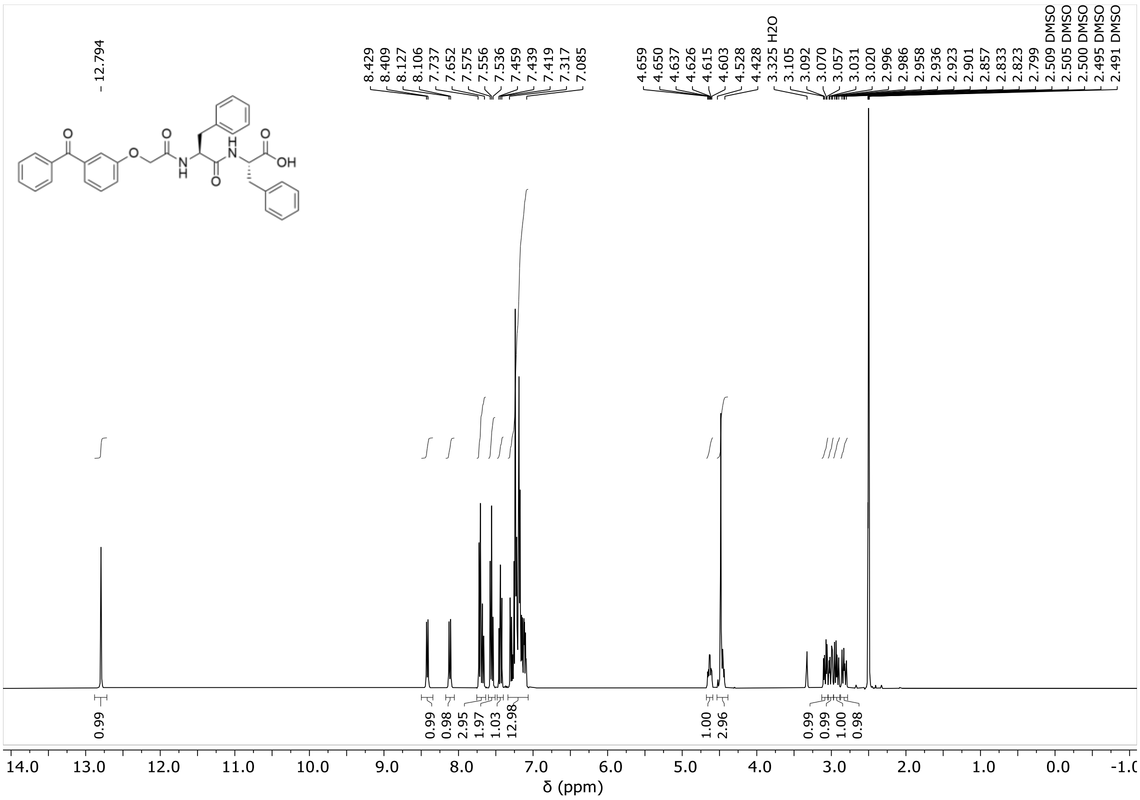

Figure S26. ^{1}H NMR (400 MHz, $CDCl_3$) of **3BPAcFF**.

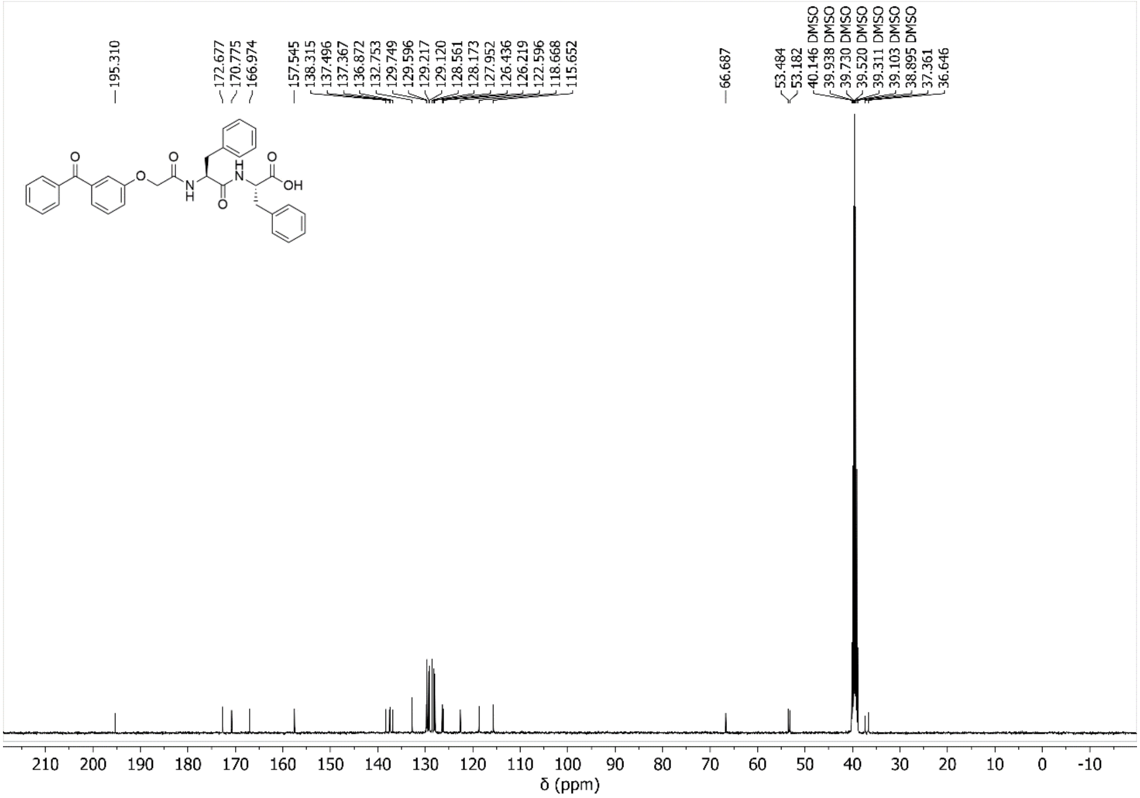

Figure S27. ^{13}C NMR (101 MHz, $CDCl_3$) of **3BPAcFF**.

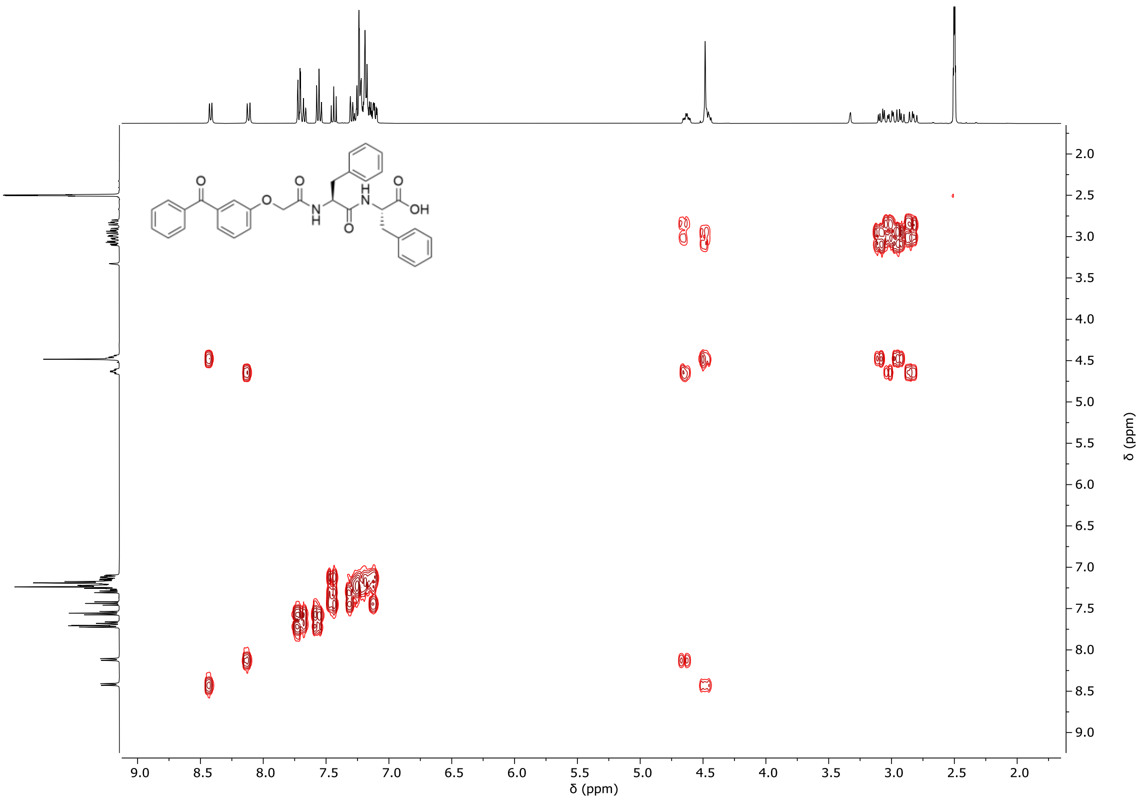

Figure S28. COSY NMR ($CDCl_3$) of **3BPAcFF**.

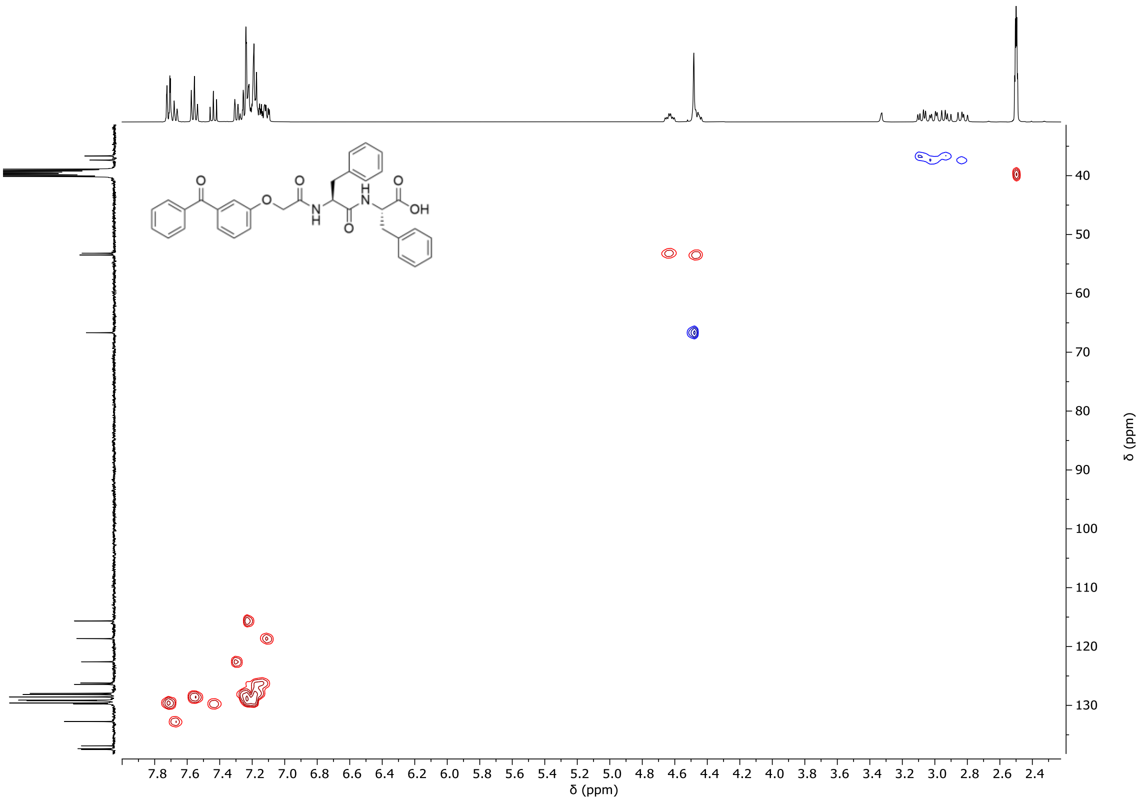

Figure S29. HSQC NMR ($CDCl_3$) of **3BPAcFF**.

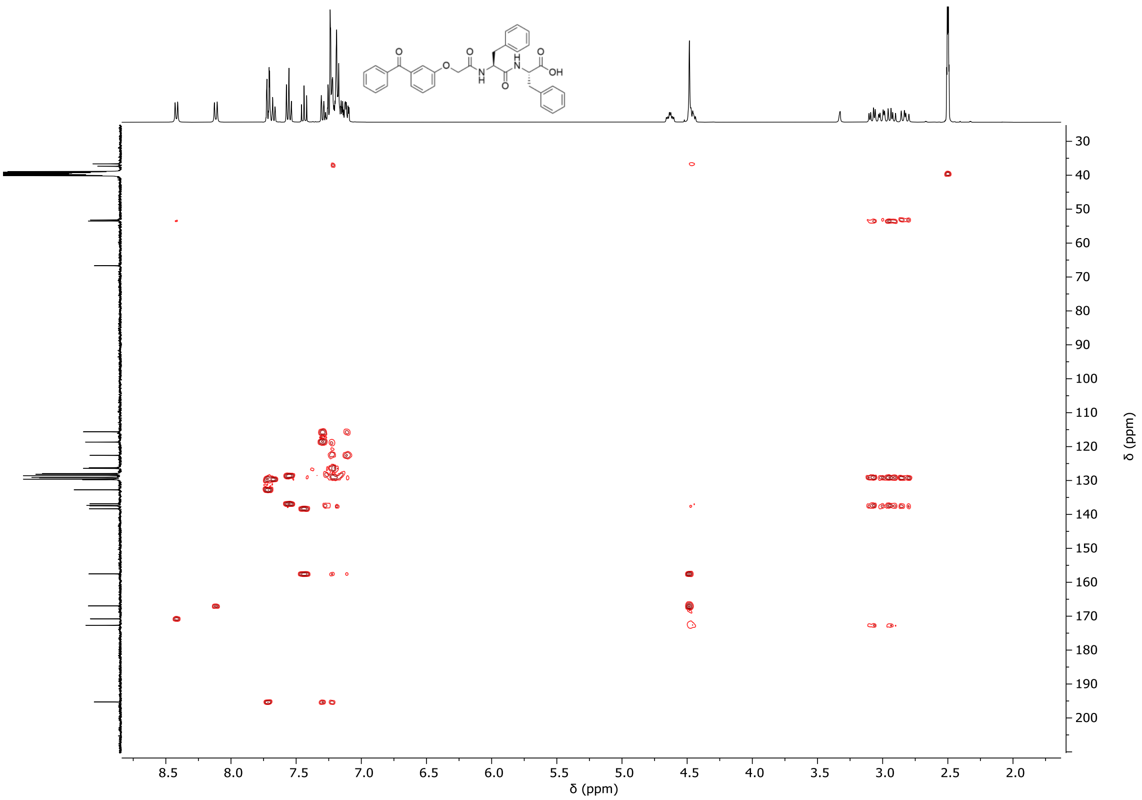

Figure S30. HMBC NMR ($CDCl_3$) of **3BPAcFF**.

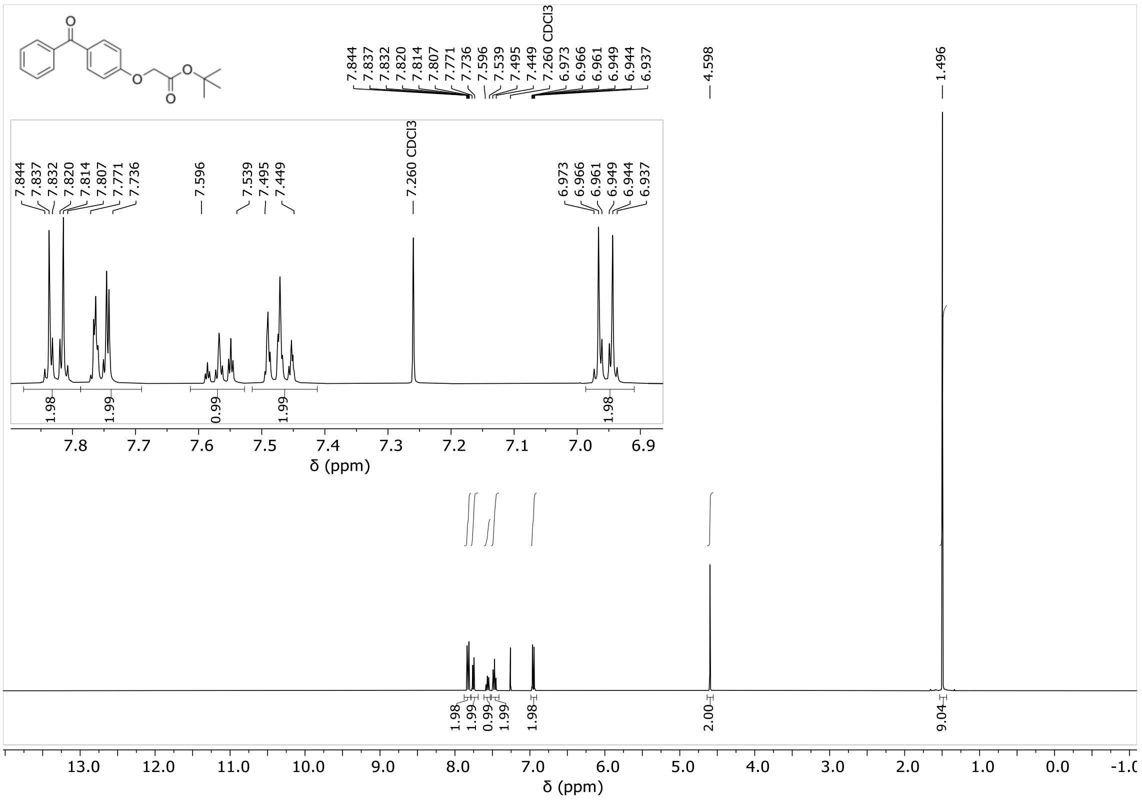

Figure S31. ^1H NMR (400 MHz, $CDCl_3$) of **4** with an expansion (insert) showing the region from 6.9–7.9.

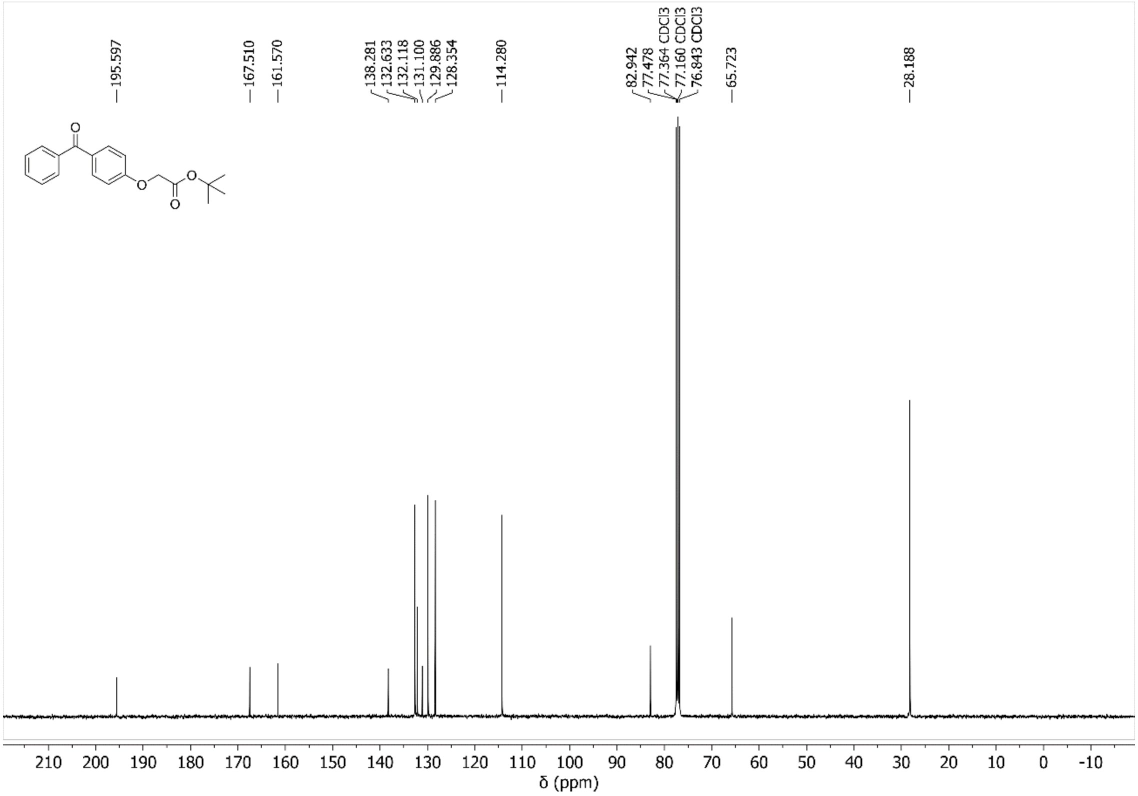

Figure S32. ^{13}C NMR (101 MHz, $CDCl_3$) of **4**.

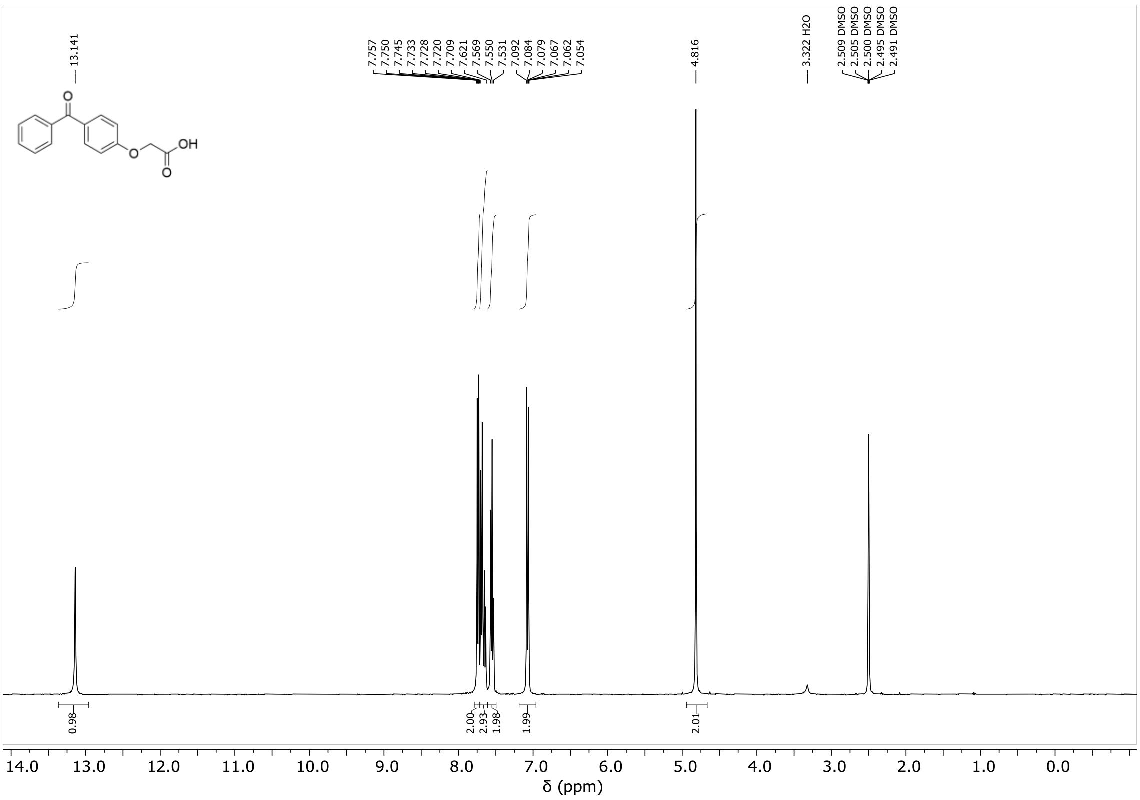

Figure S33. ^{1}H NMR (400 MHz, $CDCl_3$) of **5**.

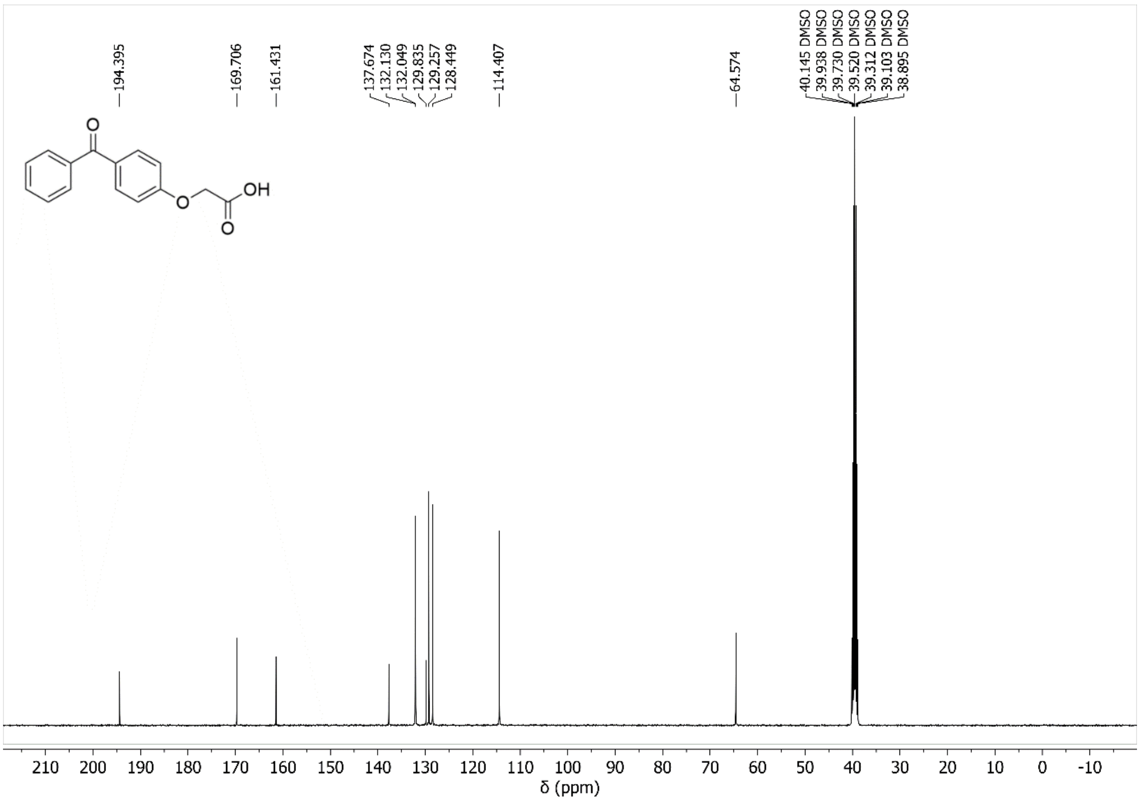

Figure S34. ^{13}C NMR (101 MHz, $CDCl_3$) of **5**.

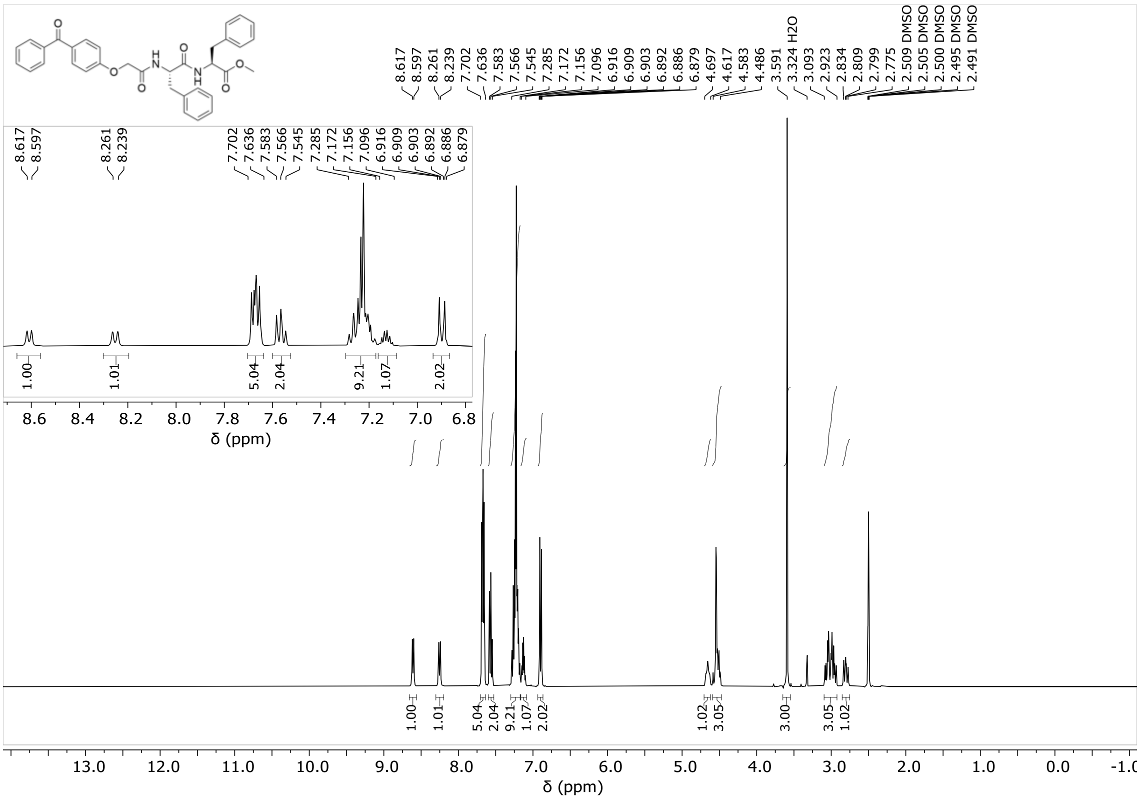

Figure S35. ^{1}H NMR (400 MHz, $CDCl_3$) of **6**.

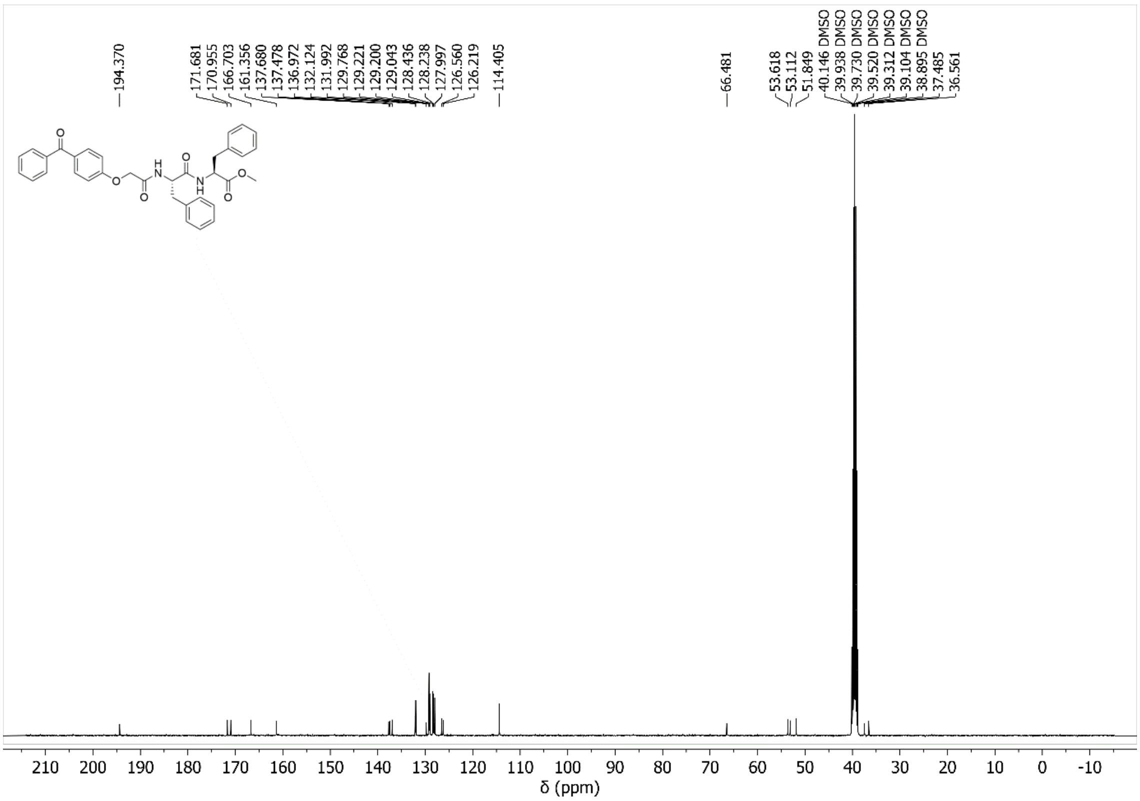

Figure S36. ^{13}C NMR (101 MHz, $CDCl_3$) of **6**.

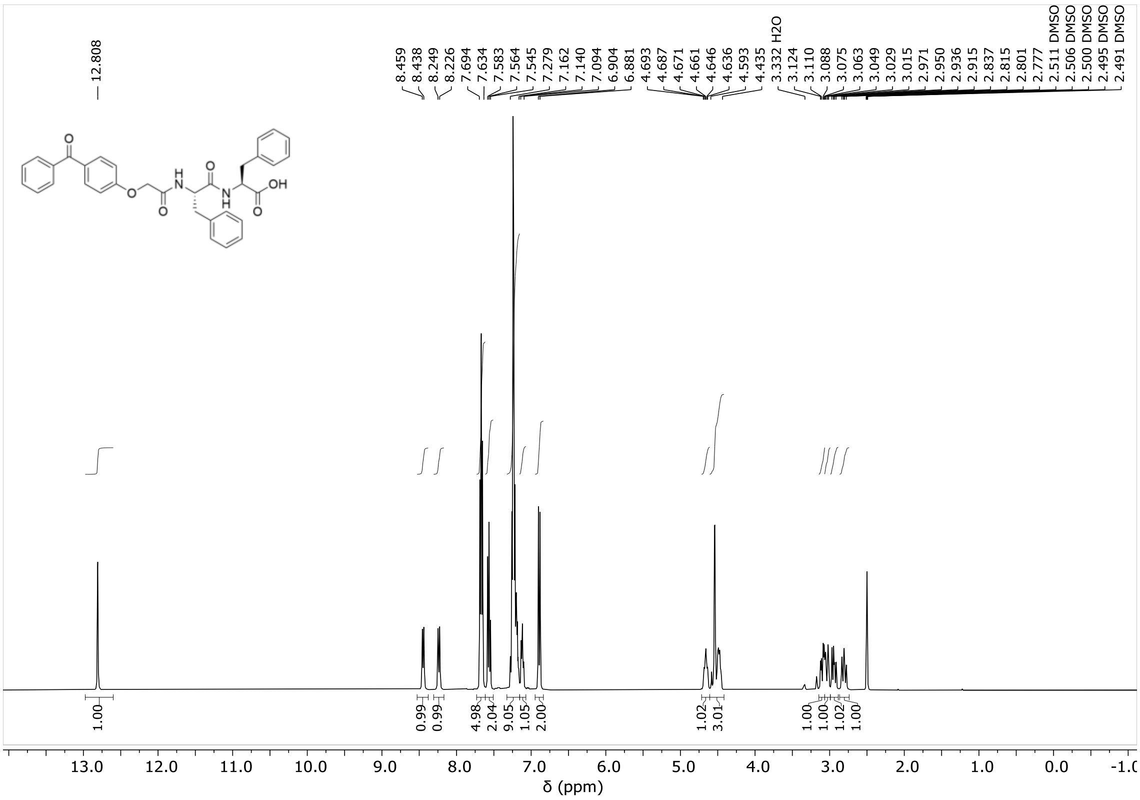

Figure S37. ^{1}H NMR (400 MHz, $CDCl_3$) of **4BPAcFF**.

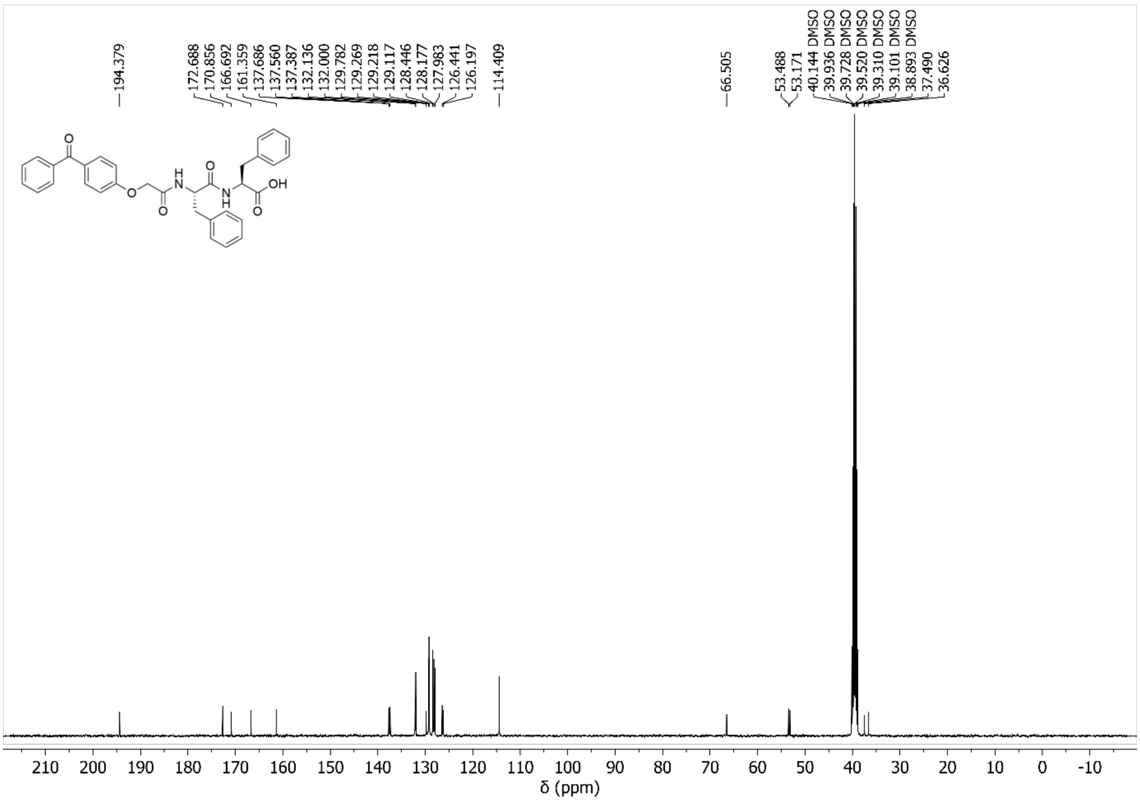

Figure S38. ^{13}C NMR (101 MHz, $CDCl_3$) of **4BPAcFF**.

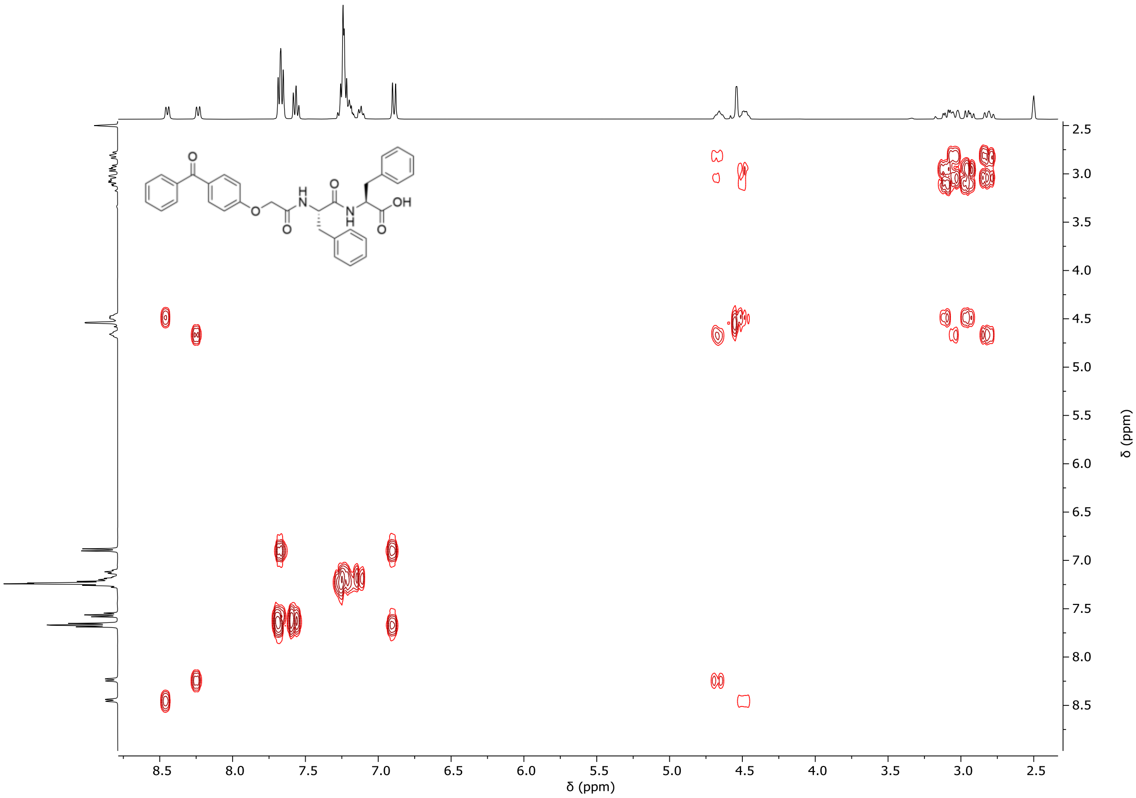

Figure S39. COSY NMR ($CDCl_3$) of **4BPAcFF**.

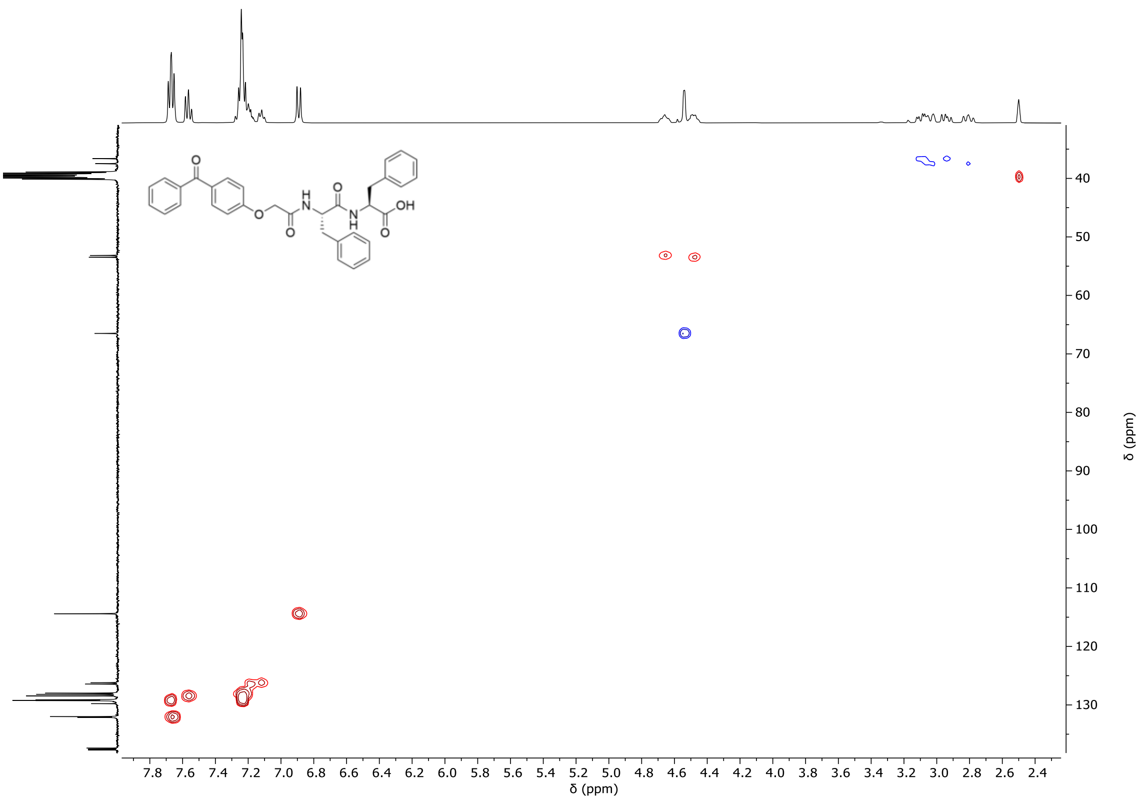

Figure S40. HSQC NMR ($CDCl_3$) of **4BPAcFF**.

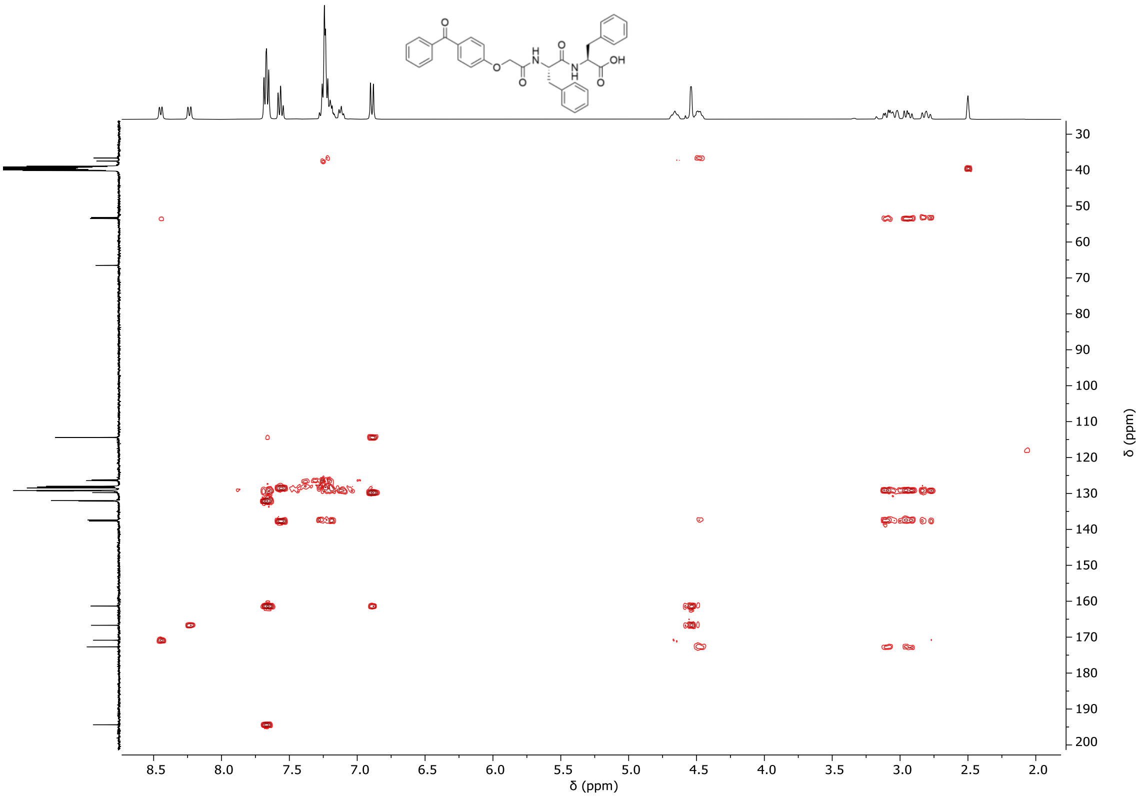

Figure S41. HMBC NMR ($CDCl_3$) of **4BPAcFF**.

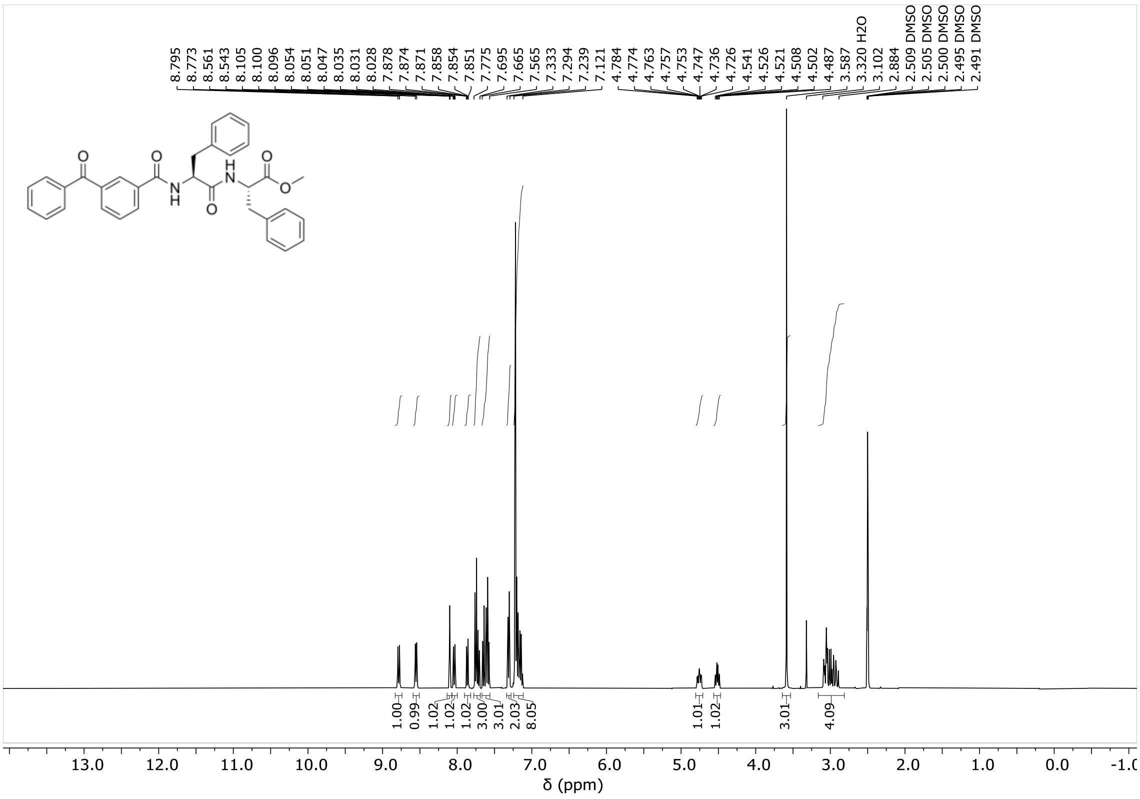

Figure S42. ^{1}H NMR (400 MHz, $CDCl_3$) of **7**.

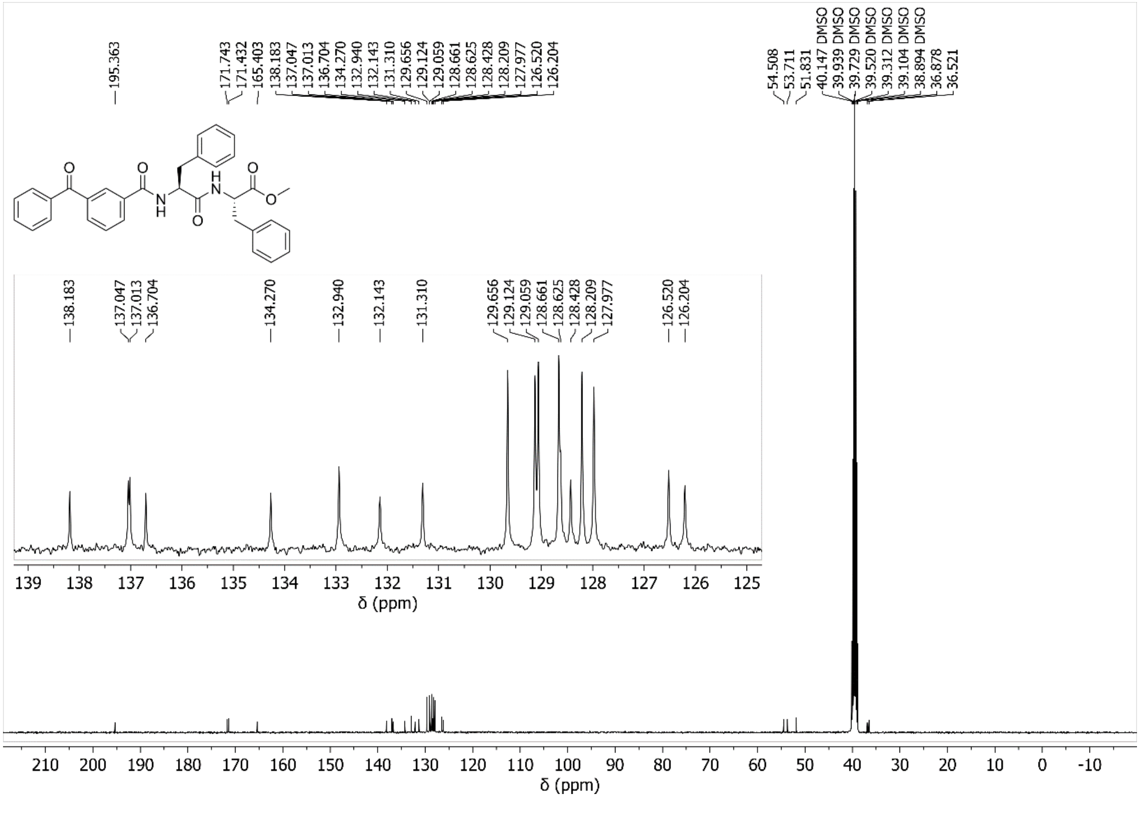

Figure S43. ^{13}C NMR (101 MHz, $CDCl_3$) of **7**.

References

1 G. Ciccone, M. Azevedo Gonzalez Oliva, N. Antonovaite, I. Lüchtefeld, M. Salmeron-Sanchez and M. Vassalli, *J. Vis. Exp.* 2022, **179**, e63401.

2 J. Filik, A. W. Ashton, P. C. Y. Chang, P. A. Chater, S. J. Day, M. Drakopoulos, M. W. Gerring, M. L. Hart, O. V. Magdysyuk, S. Michalik, A. Smith, C. C. Tang, N. J. Terrill, M. T. Wharmby and H. Wilhelm, *J. Appl. Crystallogr.*, 2017, **50**, 959–966.

3 Pauw, B. R.; Smith, A. J.; Snow, T.; Terrill, N. J.; & Thünemann, A. F. *J. Appl. Crystallogr.* 2017, **50** (6), 1800–1811.

4 S. Adorinni, G. Goti, L. Rizzo, F. Grassi, S. Kralj, F. Matroodi, M. Natali, R. De Zorzi, S. Marchesan and L. Dell'Amico, *Chem. Comm.*, 2023, **59**, 7619–7622.
